# Upper Rim-Bridged Calix[4]arenes via Cyclization of *meta* Alkynyl Intermediates with Diphenyl Diselenide

**DOI:** 10.3390/molecules29061237

**Published:** 2024-03-11

**Authors:** Anastasia Surina, Karolína Salvadori, Matěj Poupě, Jan Čejka, Ludmila Šimková, Pavel Lhoták

**Affiliations:** 1 Department of Organic Chemistry, University of Chemistry and Technology Prague, Technická 5, 166 28 Prague, Czech Republic; 2 J. Heyrovský Institute of Physical Chemistry, Czech Academy of Sciences v.v.i., Dolejškova 2155/3, 182 23 Prague, Czech Republic; 3 Department of Physical Chemistry, University of Chemistry and Technology Prague, Technická 5, 166 28 Prague, Czech Republic; 4 Department of Solid State Chemistry, University of Chemistry and Technology Prague, Technická 5, 166 28 Prague, Czech Republic; cejkaj@vscht.cz

**Keywords:** calixarene, Sonogashira coupling, *meta*-substitution, alkyne cyclization, diphenyl diselenide, *6-exo-dig* and *5-endo-dig* cyclization, regioselectivity, electrochemistry, X-ray analysis

## Abstract

A Sonogashira coupling of *meta*-iodocalix[4]arene with various terminal acetylenes confirmed that the *meta* position of calixarene is well addressable, and that both thermal and microwave protocols led to good yields of alkynylcalixarenes. Alkynes thus obtained were subjected to the ferric chloride and diphenyl diselenide-promoted electrophilic closure. It turns out that the calix[4]arenes give completely different bridging products than those described for the non-macrocyclic starting compounds. This can be demonstrated not only by the isolation of products with a six-membered ring (*6-exo-dig*), but mainly by the smooth formation of the *5-endo-dig* cyclization, which has never been observed in the aliphatic series. An attempt at electrocyclization led to a high yield of the 1,2-diketone (oxidation of the starting alkyne), again in contrast to the reaction described for the acyclic derivatives. The structures of the unexpected products were unequivocally established by X-ray analysis and clearly demonstrate how the preorganized macrocyclic skeleton favors a completely different regioselectivity of cyclization reactions compared to common aliphatic compounds.

## 1. Introduction

Calixarenes [1,2] have become an integral part of modern supramolecular chemistry, where they have found wide application due to their unique properties. Their use as building blocks or molecular scaffolds with the possibility of purposefully changing the 3D structure of the basic skeleton (e.g., immobilization of four different conformations (atropisomers) in the case of calix[4]arene) makes them an ideal choice for the design and synthesis of new receptors and similar functional molecular systems [3,4].

Calix[4]arenes show an almost unlimited possibility of derivatization of the basic skeleton, enabling the introduction of desired functional groups at virtually any place in the macrocycle. Currently, many strategies are known leading to regio- or stereo-selective derivatization of basic calix[4]arene, usually based on a combination of alkylation reactions on the lower rim (OH groups) and electrophilic substitution of the upper rim (aromatic part). In this context, however, we come across a limitation given by the directing effects of the substituents present in the macrocycle (OH, OR), where electrophilic substitution takes place preferentially in the *para* position to these groups. For this reason, in calixarene chemistry, *meta* substitution [5] represents a synthetic challenge, and the *meta* substituted derivatives appear only rarely.

**Scheme 1:**
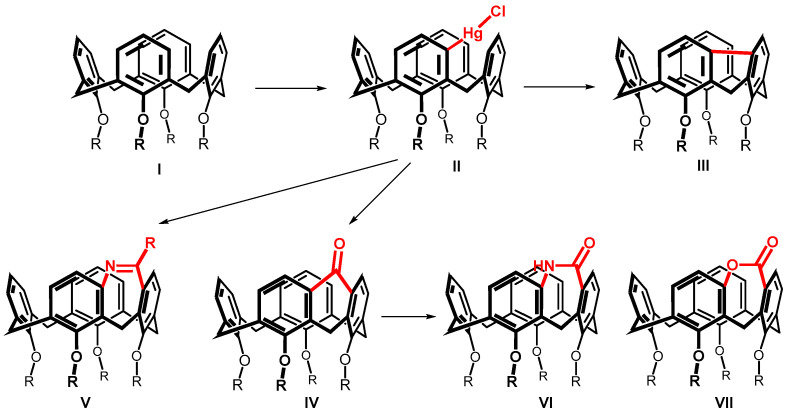
The upper rim-bridged calix[4]arenes bearing five-membered, six-membered and seven-membered rings within the molecules.

Systematic studies on the calix[4]arene **I** reactivity surprisingly revealed that the reaction with mercury trifluoroacetate (Hg(TFA)_2_) leads to a direct substitution [6] of the hitherto practically inaccessible *meta* position (Scheme 1). The formation of an organomercury compound **II** then enabled the preparation of new types of calixarenes with a *meta*-*meta* bridged upper rim [7]. These compounds are not only interesting because of their highly increased rigidity (see e.g., **III** and **IV**), but often also represent inherently chiral systems potentially suitable for the design of chiral receptors (**V**–**VII**) [8,9].

The bridging of two adjacent aromatic nuclei in the *meta* position leads to the formation of condensed tricyclic systems having a five-, six-, or seven-membered ring (Scheme 1). While a number of systems having a five- or six-membered ring have been prepared, only three seven-membered systems (**V**–**VII**) have been described so far [8,9]. Thus, compounds of type **V** were obtained by cyclization of the corresponding *meta*-amides, and compounds **VI** and **VII** were obtained from ketone **IV** using a ring extension via Baeyer-Villiger or Beckmann rearrangements.

**Scheme 2:**
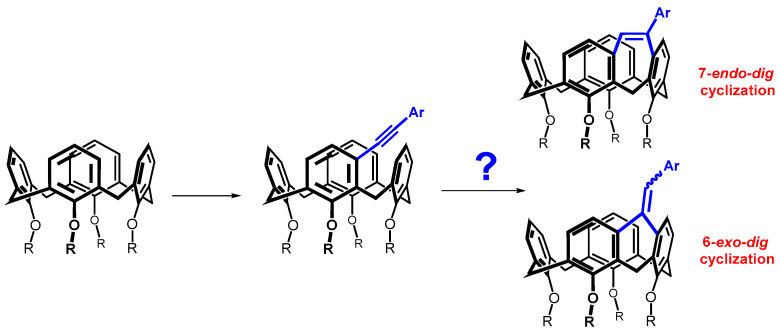
*meta*-Alkynylcalix[4]arenes and possible regioisomers formed by their cyclization.

Since different cycle sizes introduce different internal strains into the macrocyclic system, we thought it interesting to apply a bridging reaction that could provide different sizes of cyclic moieties. Such a reaction could be the cyclization of alkynes proceeding either by the *exo-dig* or *endo-dig* mechanism (Baldwin’s rules), where both processes are allowed (Scheme 2) [10]. This paper describes an effort to prepare the above systems, which show a surprising preference of calix[4]arene skeleton compared to acyclic molecules.

## 2. Results and Discussion

**Scheme 3:**
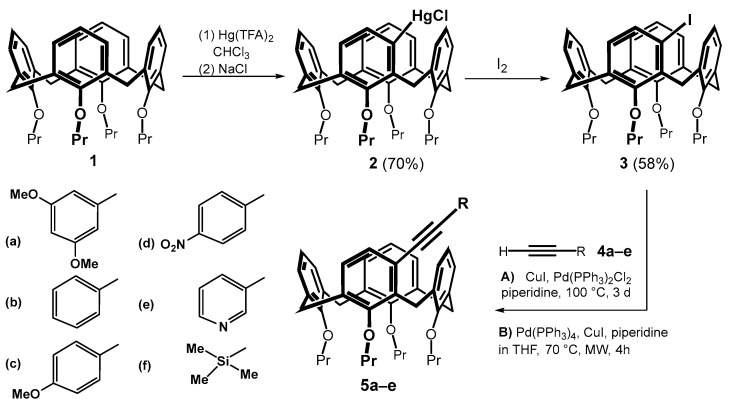
Sonogashira coupling of *meta*-substituted calixarenes.

Starting calix[4]arene **1** immobilized in the *cone* conformation was successively reacted with mercury(II) trifluoroacetate (Hg(TFA)_2_) and sodium chloride using a literature procedure [6]. The corresponding *meta*-substituted chloromercurio derivative **2** was obtained regioselectively in good yield (70%). A subsequent reaction with iodine provided the *meta*-iodo derivative **3** in 58% yield (Scheme 3) [8]. The introduction of triple bond into the calixarene skeleton was carried out by Sonogashira coupling [11,12], which is well known for terminal alkynes. This reaction has already been successfully used several times in calixarene chemistry, but it involved either the *para* position of the aromatic subunit [13,14,15] or the transformation of the lower rim of the macrocycle [16,17]. The reactivity of the halogen in the *meta* position was therefore unknown. To study this cross-coupling of compound **3**, a series of substituted aromatic acetylenes **4a**–**e** completed with a silyl- derivative **4f** was selected.

Based on the literature search [11], we applied two different implementations of the Sonogashira reaction: (i) classical heating on a magnetic stirrer and (ii) application of microwave reactor. Thus, heating compound **3** with **4a** and Pd(PPh_3_)_2_Cl_2_/CuI in piperidine at 100 °C for 3 days gave the expected product **5a** in 89% yield. The reaction carried out in a microwave reactor (Pd(PPh_3_)_4_/CuI, piperidine in THF) at 70 °C for 4 h provided the same product in 74% yield. A similar trend, i.e., a higher yield upon classical heating, was observed for all electron-donating substituents (**5a**–**c**) and the silyl-substituted derivative **5f**, see Table 1. In contrast, both derivatives bearing electron-withdrawing substituents **5d** and **5e** were obtained in a higher yield upon microwave reaction (compare 33% vs. 47% for **5e**).

**Scheme 4:**
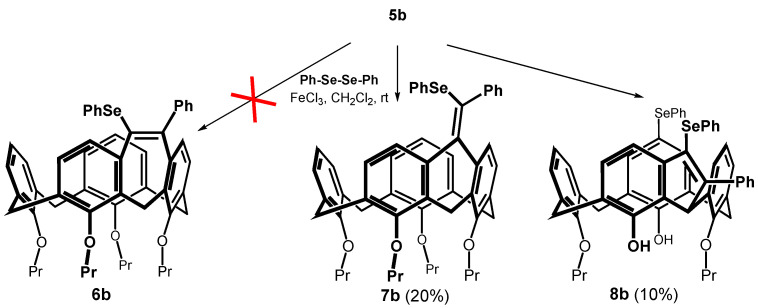
An attempted cyclization of **5b** with Ph_2_Se_2_/FeCl_3_ system.

Alkynes thus obtained were subjected to the recently described iron(III) chloride and diorganyl diselenide-promoted electrophilic closure [18,19,20], giving selectively the seven-membered cyclization products [18]. The reaction of **5b** carried out under the conditions described in the literature (alkyne + Ph_2_Se_2_ (0.55 equiv.) + FeCl_3_ (1.0 equiv.) in CH_2_Cl_2_ at RT) gave practically unreacted starting compounds. In order to achieve higher conversion of the calixarene derivatives, we increased the number of equivalents of both reagents, and the reaction was carried out at a higher temperature (Scheme 4). Thus, heating of **5b** with 2.5 equivs. of iron(III) chloride and 2 equivs. of Ph_2_Se_2_ in dichloromethane at 30 °C for 4 h gave a crude reaction mixture with 42% of unreacted starting compound. The column chromatography on silica gel provided an expected cyclized product (20% yield) as indicated by the HRMS (ESI+) peak at *m*/*z* = 871.3240 well corresponding to the theoretical value for C_54_H_56_O_4_SeNa^+^ ion (*m*/*z* = 871.3245). Other fractions contained derivatives that were apparently bridged but lacked varying numbers of propyl groups (^1^H NMR), indicating a concurrent dealkylation reaction. The ^1^H NMR spectrum of the product (CDCl_3_, 400 MHz) showed eight doublets with typical geminal coupling constants (13–15 Hz) corresponding to the signals of the methylene bridges (3.00 to 4.7 ppm) in the *cone* conformation. Unfortunately, despite our efforts, it was not possible to decide between the two possible structures **6b** and **7b** using NMR spectroscopy.

**Figure 1:**
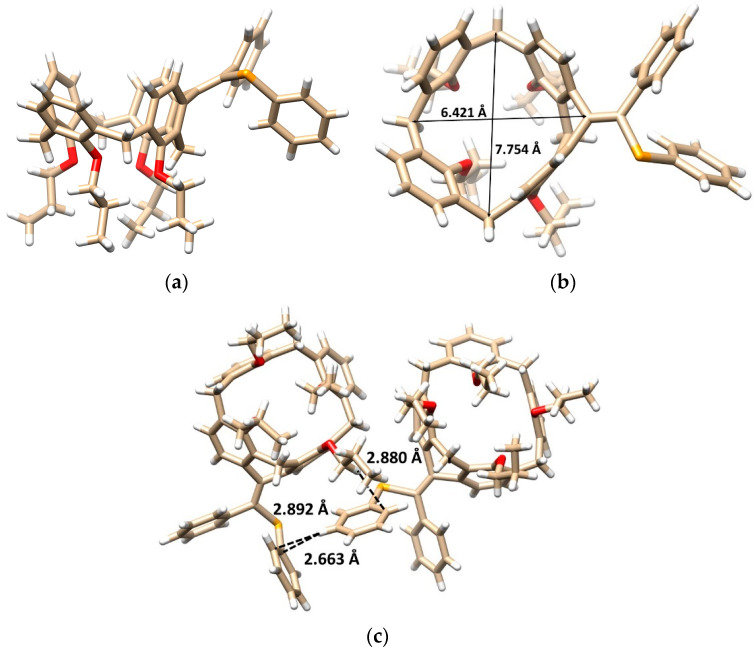
Single crystal X-ray structure of **7b**: (**a**) the side view, (**b**) top view showing the main diagonals, (**c**) dimeric motif within the crystal packing based on the CH··· π and the π-π (T-shaped) interactions (red = O, orange = Se, white = H, beige = C atoms).

The final proof of the product structure was performed using single crystal X-ray diffraction analysis. The product is in the form of a six-membered ring structure **7b** crystallized in a triclinic crystal system, space group *P*-1, and adopted the *cone* conformation (Figure 1a). The alkylidene bridge results not only in the rigidification of the calixarene skeleton, but also in the partial flattening of the originally square shape of the cavity. This can be demonstrated on different lengths of the main diagonals (the distance between two opposite Ar-CH_2_-Ar carbons, compare 7.754 vs. 6.421 Å, Figure 1b).

The presence of two different substituents on the double bond makes the molecule inherently chiral, with both enantiomers included in the unit cell as the independent molecules (racemic mixture). The opposite enantiomers are held together throughout the crystal packing of **7b** (Figure 1c) by the CH-π interactions between the O-CH_2_- group and the phenyl selenyl moiety (CH···C_Ar_ distance = 2.880 Å). At the same time, the close contacts between PhSe groups can be found, based on the T-shaped π-π interactions [21,22] between phenyl moieties (Figure 1c).

Compound **5a** also afforded the cyclization product **7a** in low yield, which, however, could not be sufficiently purified for analysis. Nevertheless, the structure was determined by comparing its ^1^H NMR spectrum with that of **7b**. At the same time, the HRMS (ESI+) showed a molecular peak at *m*/*z* = 931.3453 (compared with the theoretical value 931.3447 for the [M + Na]^+^ ion). Under the same conditions, nitro compound **5d** gave only a trace amount of product (TLC, HRMS), indicating a negative influence of the electron-withdrawing group on the course of the reaction (conversion ca 1% (^1^H NMR)). Similar conclusions can be drawn for compound **5e**.

Since all cyclization attempts exhibited low conversion of the starting compound, we tried to increase the reaction temperature by using DCE as a solvent. Reaction of **5b** with Ph_2_Se_2_ and FeCl_3_ (both 2.5 equiv) under reflux (85 °C) gave a very complex reaction mixture. As it was proved by means of model reactions, FeCl_3_ is a strong dealkylating agent under these conditions, so that a partial non-selective dealkylation of both starting substances and products took place. The isolation by preparative TLC gave the bridged product **8b** in low yield (Scheme 4). The ^1^H NMR spectra (400 MHz, CDCl_3_) measured at room temperature showed two singlets at 8.21 and 8.31 ppm, indicating the presence of free phenolic groups. The aliphatic part of the spectrum contains signals of only two propyl groups, which proves the partial dealkylation of the starting macrocycle. Interestingly, instead of 8 doublets with a geminal coupling constants, only two sets of three doublets (*J* = 12.5–13.7 Hz) were seen for the methylene linkages (3.20, 3.28 and 3.37 ppm for the equatorial and 4.06, 4.36 and 4.44 ppm for axial CH bonds). The last bridge showed only singlet at 5.42 ppm corresponding to one hydrogen. This suggests that one methylene group was transformed during the reaction, but the actual structure remained unknown.

**Figure 2:**
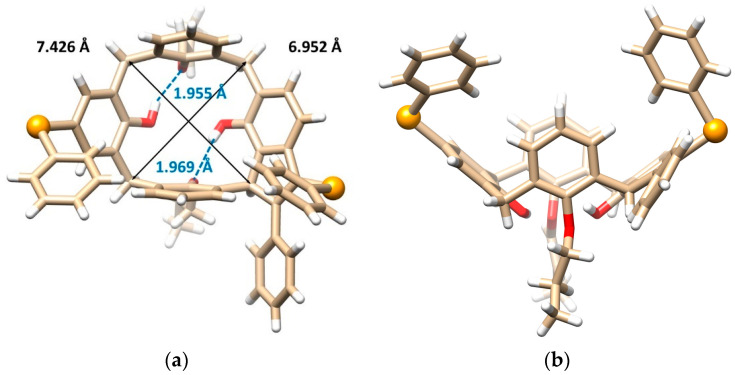

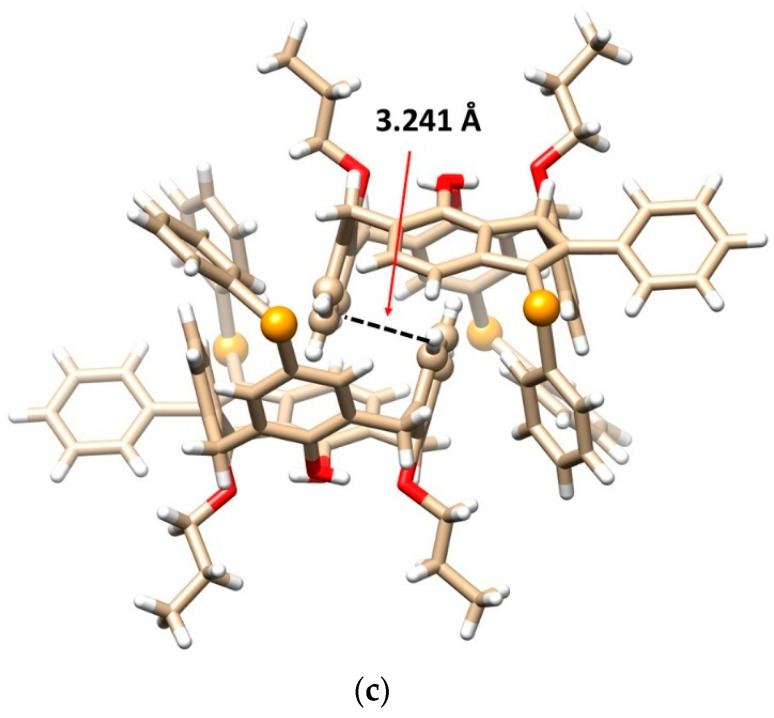
Single crystal X-ray structures of compound **8b**: (**a**) the top view and (**b**) the side view, showing the *cone* conformation with the corresponding hydrogen bods (dashed lines) and the main diagonals (solid lines), (**c**) dimeric self-inclusion motif within the crystal packing based on the π-π interactions (dashed line) between opposite enantiomers (Se atoms and interacting aromatic units shown as balls, the CH_2_Cl_2_ solvate molecules removed for better clarity), (red = O, orange = Se, white = H, beige = C atoms).

This problem was unequivocally solved by single crystal X-ray diffraction analysis. Compound **8b** crystallizes in a monoclinic crystal system, space group P2_1_/n, and forms a CH_2_Cl_2_ solvate with 1:1 stoichiometry. As shown in Figure 2a,b, calixarene adopts the *cone* conformation containing the five-membered ring formed by the addition of the methylene bridge to the alkyne moiety. The unexpected cyclization is accompanied by the introduction of a second PhSe- group into the *para* position of the opposite aromatic subunit. Both phenols bearing the substitution are dealkylated at the same time. The *cone* conformation is held by hydrogen bonds between the free OH groups and the oxygens of the neighbor propoxy functions (OH···O distance = 1.955 and 1.969 Å). The presence of the indene moiety within the backbone has surprisingly little effect on the shape of the cavity, which can be demonstrated by its approximately square shape (the length of the main diagonals is 6.952 vs. 7.426 Å), see Figure 2a.

Since molecule **8b** is inherently chiral, the crystal contains both enantiomers (racemic mixture), which form an interesting dimeric motif (Figure 2c). The aromatic subunits of opposite enantiomers bearing the propoxy groups are nested into each other’s adjacent cavity to form self-included dimers. The aromatic subunits are ideally coplanar interacting via the π-π interactions with each other in the so-called parallel stacked arrangement. The interplanar spacing of these aromatic subunits is exceptionally short (only 3.241 Å, while the usual distance is around 3.5 Å), indicating unusually strong π-π interactions.

**Scheme 5:**
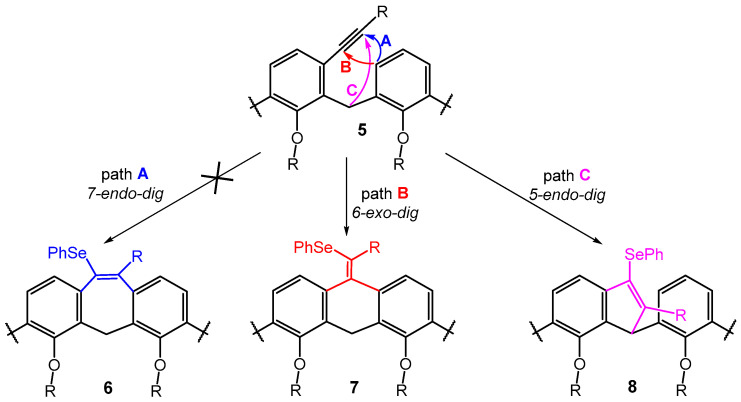
The cyclization of *meta*-alkynylcalix[4]arenes the three different arrow colors show the three possible reaction pathways leading to the corresponding products.

The formation of product **8b** is completely unexpected and to the best of our knowledge, there is no example of similar structures in the literature. The selenium-mediated electrophilic cyclization reaction is well known in the art and has been used to construct a variety of structures [19,23,24,25]. On the other hand, although the FeCl_3_/Ph_2_Se_2_ promoted cyclization of *o*-alkynyl substituted diaryl methanes (a structural fragment within a calixarene moiety) has been investigated in detail, [18] the formation of a five-membered product of type **8** has never been observed (Scheme 5). The formation of indene structures [26] from *o*-substituted aryl alkynes is known, but it is based on direct C(sp^3^)-H activation using Au(I) [27] or Pt(II) [28] catalysts. A similar reaction was also achieved by the intramolecular addition of triarylmethanes [29] to the alkynyl group in the *ortho* position induced by the presence of a base (*t*-BuOK), but it was never reported for selenium-induced cyclization.

Furthermore, it is noteworthy that the aforementioned FeCl_3_/Ph_2_Se_2_ cyclization studies describe *endo-dig* cyclization leading to products of type **6** as the sole exclusive reaction pathway (path A, Scheme 5) [18,19]. In this respect, the calix[4]arene skeleton represents a unique system, since we did not observe the formation of a seven-membered cycle in any case. A possible explanation lies in the fact that the relative position of the neighboring aromatic nuclei imposed by the macrocyclic structure favors *6-exo-dig* (type **7**) or *5-endo-dig* (type **8**) over *7-endo-dig* (type **6**) cyclization, i.e., reaction pathways that are unknown for common acyclic derivatives (Scheme 5).

**Scheme 6:**
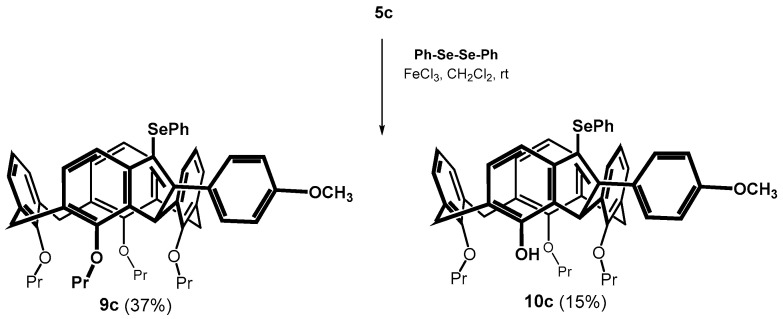
Cyclization of methoxy derivative **5c** (*5-endo dig*).

From this point of view, the reaction of the methoxy derivative **5c** was very surprising. The cyclization of the alkyne proceeded smoothly already at 30 °C (100% conversion), whereby two products were isolated from the reaction mixture (Scheme 6). The HRMS (ESI+) of the main product **9c** (37% yield) showed a molecular peak at *m*/*z* = 901.3345 which corresponded to the expected mass of the cyclized product *m*/*z* = 901.3342 ([M + Na]^+^ ion). The ^1^H NMR (CDCl_3_, 298 K, 400 MHz) comprised two sets of three doublets for the methylene bridges (*J* ≈ 13.3 Hz), together with a singlet at 5.70 ppm (1H). This splitting pattern is very similar to that of compound **8b** and refers to cyclization via a single methylene bridge. The second isolated product was assigned as the same type of structure, but lacking one propyl unit. Based on two-dimensional NMR experiments (HSQC, HMBC) it appears most likely that this derivative has the structure **10c** with the OH group in the indene fragment (Scheme 6). Thus, the result again reflects the remarkable regioselectivity of the reaction, where the cyclization proceeds only through the adjacent methylene bridge, while the expected bridging of the adjacent aromatic subunit was not observed at all.

**Scheme 7:**
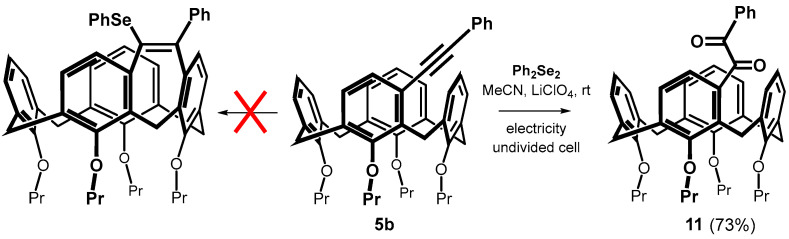
Attempted electrocyclization of phenyl derivative **5b** under aerobic conditions.

The FeCl_3_-mediated electrophilic selanylation/annulation of *meta*-substituted calixarenes showed very unexpected results; however, the use of a Lewis acid led to relatively low yields due to dealkylation of the lower rim of the calixarene. To avoid this, we decided to apply the recently described method based on the action of diphenyl diselenide under electrochemical conditions [30,31]. Thus, the reaction of **5b** with 0.6 equiv. of Ph_2_Se_2_ in MeCN in the presence of LiClO_4_ as an electrolyte was carried out in an undivided cell under constant current electrolysis (CCE) at 10 mA for 3 h under aerobic atmosphere. Although the original publications promise a virtually quantitative bridging yield, we were unable to demonstrate any cyclization. The only resulting product **11** was isolated in 73% yield (Scheme 7), and its 1,2-diketone [32] structure was confirmed by a combination of HRMS and NMR spectroscopy (see typical carbonyl signals at 197.92 and 195.89 ppm in ^13^C NMR spectrum). Altering the reaction parameters, such as using a different electrode material (Pt vs. glassy carbon), did not lead to any changes. We attempted to eliminate the unwanted oxidation by performing electrolysis at a constant potential (+1.5 V), which was chosen from appropriate cyclic voltammogram (Figure S78) as the first irreversible step of Ph_2_Se_2_. Unfortunately, compound **11** was still the only isolated product. Finally, carrying out the reaction in a degassed solvent (purged with a stream of argon) prevented oxidation but gave no reaction. It indicates that the mentioned methodology is not suitable for the electrocyclization of *meta*-alkynyl calixarenes.

## 3. Materials and Methods

### 3.1. General Experimental Procedures

All commercially obtained chemicals were purchased from commercial sources and used as received without further purification. Solvents were dried and distilled using conventional methods. NMR spectra were performed on Agilent 400-MR DDR2, JEOL-ECZL400G (^1^H: 400 MHz, ^13^C: 100 MHz) and Bruker Avance DRX 500 (^1^H: 500 MHz, ^13^C: 125 MHz) spectrometers at 298 K. Deuterated solvents used are indicated in each case. Chemical shifts (*δ*) are reported in parts per million (ppm) and were referenced to residual peak of the solvent or TMS as an internal standard; coupling constants (*J*) are expressed in Hz. NMR data were processed and displayed using MestReNova (Version: 14.3.3) and TopSpin (Version 3.6.5) softwares. The IR spectra were measured on an FT–IR spectrometer Nicolet iS50 (Thermo-Nicolet, Waltham, MA, USA) with a heatable Golden Gate Diamante ATR–Unit GladiATR (Specac, Orpington, UK). 64 scans for one spectrum were co-added at a spectral resolution of 4 cm^–1^. Electrospray ionization mass spectra (ESI-MS) were recorded using a LTQ Orbitrap Velos—hybrid ion-trap-orbitrap (Thermo Scientific, Waltham, MA, USA). The purity of the substances and courses of the reactions were monitored by thin layer chromatography (TLC) using silica gel 60 F_254_ on aluminum-backed sheets (Merck, Lowe, NJ, USA) and analyzed at 254 nm. The column chromatography was conducted on silica gel 60 with particle size 0.063–0.200 mm (Merck). For melting point studies, Heiztisch Mikroskop—Polytherm A (Wagner & Munz, Munich, Germany) was used.

### 3.2. Synthetic Procedures

#### 3.2.1. Preparation of Alkynylcalixarenes **5a**–**f**

##### General Procedure for the Synthesis of Alkynylcalixarenes **5a**–**f** (Method A)

Starting calix[4]arene **3** (1 equiv), CuI (0.1 equiv) and Pd(PPh_3_)_2_Cl_2_ (0.05 equiv) were dissolved in 3–4 mL of dry piperidine under an Ar atmosphere. Then, appropriate alkyne **4a**–**f** (1.1 equiv) was added, and the reaction mixture was stirred and heated at 100 °C for 3 days. The mixture was cooled to RT, and the solvent was evaporated under reduced pressure. The crude product was extracted (3 × 30 mL) with dichloromethane (DCM), the organic phase was washed with water, dried over MgSO_4_, filtered and evaporated to dryness. The residue was purified by column chromatography on silica gel (eluent: DCM: cyclohexane) to afford the title compounds.

##### General Procedure for the Synthesis of Alkynylcalixarenes **5a**–**f** (Method B)

A mixture of starting calix[4]arene **3** (1 equiv), alkyne **4a**–**f** (4 equiv), piperidine (4 equiv), CuI (0.2 equiv), Pd(PPh_3_)_4_ (0.1 equiv) and dry THF was stirred under argon atmosphere at 70 °C for 4 h in the microwave reactor. The mixture was then evaporated to dryness, the residue was extracted with DCM (3 × 30 mL), washed with water and dried over MgSO_4_. The purification of the crude product was done in the same way as described in Method A.

##### Alkynylcalixarene **5a**

Method A: Calixarene **3** (0.209 mmol, 150 mg), 3,5-dimethoxyphenylacetylene (0.230 mmol, 37 mg), CuI (0.0209 mmol, 4 mg) and Pd(PPh_3_)_2_Cl_2_ (0.0104 mmol, 7 mg) were used under standard conditions; the product was purified by column chromatography on silica gel (DCM/cyclohexane 1:1) to afford **5a** in 89% yield (140 mg) as a white solid.

Method B: Calixarene **3** (0.139 mmol, 104 mg), 3,5-dimethoxyphenylacetylene (0.556 mmol, 90 mg), CuI (0.028 mmol, 5 mg), piperidine (0.417 mmol, 35 µL) and Pd(PPh_3_)_4_ (0.0139 mmol, 16 mg) were used under standard conditions. The product **5a** (77 mg, 74%) was obtained by column chromatography on silica gel (DCM/cyclohexane 60:40) as a white solid.

Data for compound **5a**: M.p. = 105–107 °C. ^1^H NMR (CDCl_3_, 500 MHz, 298 K) *δ* 7.19 (d, *J* = 7.7 Hz, 1H, Ar-H), 7.08 (d, *J* = 7.7 Hz, 2H, Ar-H), 7.06 (d, *J* = 7.7 Hz, 1H, Ar-H), 6.89 (t, *J* = 7.5 Hz, 1H, Ar-H), 6.67 (d, *J* = 2.3 Hz, 2H, Ar-H), 6.43 (t, *J* = 2.3 Hz, 1H, Ar-H), 6.33 (dd, *J* = 7.5 Hz, *J* = 1.1 Hz, 1H, Ar-H), 6.26 (t, *J* = 7.5 Hz, 1H, Ar-H), 6.24 (t, *J* = 7.5 Hz, 1H, Ar-H), 6.15 (d, *J* = 7.5 Hz, 2H, Ar-H), 6.13 (d, *J* = 7.5 Hz, 1H, Ar-H), 4.47 (d, *J* = 13.2 Hz, 1H, Ar-CH_2_-Ar), 4.46 (d, *J* = 2.8 Hz, 1H, Ar-CH_2_-Ar), 4.44 (d, *J* = 2.8 Hz, 1H, Ar-CH_2_-Ar), 4.36 (d, *J* = 13.2 Hz, 1H, Ar-CH_2_-Ar), 4.01 (m, 4H, -O-CH_2_-), 3.95 (d, *J* = 13.2 Hz, 1H, Ar-CH_2_-Ar), 3.79 (s, 6H, -O-CH_3_), 3.73 (m, 2H, -O-CH_2_-), 3.69 (m, 2H, -O-CH_2_-), 3.16 (d, *J* = 13.2 Hz, 1H, Ar-CH_2_-Ar), 3.15 (d, *J* = 13.2 Hz, 2H, Ar-CH_2_-Ar), 1.98 (m, overlap, 4H, -CH_2_-CH_3_), 1.88 (m, 4H, -CH_2_-CH_3_), 1.10 (t, *J* = 7.3 Hz, 6H, -O-(CH_2_)_2_-CH_3_), 0.91 (t, *J* = 7.4 Hz, 3H, -CH_3_), 0.90 (t, *J* = 7.4 Hz, 3H, -CH_3_). ^13^C NMR (CDCl_3_, 126 MHz, 298 K) *δ* 160.47, 157.98, 157.88, 155.17, 155.16, 139.31, 137.99, 137.01, 136.89, 133.47, 133.22, 132.92, 132.67, 128.84, 128.78, 128.51, 127.57, 127.41, 127.33, 127.24, 126.21, 125.01, 122.10 (2C), 121.82, 121.78, 109.33, 101.42, 91.1, 88.83, 76.94, 76.90, 76.58, 76.44, 55.43 (2C), 31.04, 30.97, 30.96, 28.11, 23.50 (2C), 22.99, 22.97, 10.79, 10.78, 9.86, 9.84 ppm. HRMS (ESI^+^) calcd for C_50_H_56_O_6_ 775.3969 [M + Na]^+^, found *m*/*z* 775.3971 [M + Na]^+^. IR (KBr) ν 2959, 2932, 2873, 1587, 1453, 1418, 1205, 1193, 1154, 1060, 965, 756 cm^−1^.

##### Alkynylcalixarene **5b**

Method A: Calixarene **3** (0.315 mmol, 226 mg), phenylacetylene (0.3465 mmol, 40 µL), CuI (0.0315 mmol, 6 mg) and Pd(PPh_3_)_2_Cl_2_ (0.0157 mmol, 11 mg) were used under standard conditions; the product was purified by column chromatography on silica gel (DCM/cyclohexane 1:1) to afford **5b** in 96% yield (209 mg) as a yellow oil.

Method B: Calixarene **3** (0.139 mmol, 103 mg), phenylacetylene (0.556 mmol, 60 µL), CuI (0.028 mmol, 5 mg), piperidine (0.417 mmol, 40 µL) and Pd(PPh_3_)_4_ (0.0139 mmol, 16 mg) were used under standard conditions. The product **5b** (59 mg, 62%) was obtained by column chromatography on silica gel (DCM/cyclohexane 70:30) as a yellow oil.

Data for compound **5b**: ^1^H NMR (CDCl_3_, 500 MHz, 298 K) *δ* 7.51 (dd, *J* = 7.7 Hz, *J* = 1.6 Hz, 2H, Ar-H), 7.27–7.35 (m, 3H, Ar-H) 7.19 (d, *J* = 7.7 Hz, 1H, Ar-H), 7.08 (d, *J* = 7.4 Hz, 2H, Ar-H), 7.06 (d, *J* = 7.7 Hz, 1H, Ar-H), 6.90 (t, *J* = 7.7 Hz, 1H, Ar-H), 6.34 (d, *J* = 7.7 Hz, 1H, Ar-H), 6.26 (d, *J* = 7.7 Hz, 1H, Ar-H), 6.22 (d, *J* = 7.7 Hz, 1H, Ar-H), 6.14 (d, *J* = 8.2 Hz, 2H, Ar-H), 6.12 (d, *J* = 7.7 Hz, 1H, Ar-H), 4.46 (d, *J* = 13.4 Hz, 1H, Ar-CH_2_-Ar), 4.45 (d, *J* = 13.4 Hz, 1H, Ar-CH_2_-Ar), 4.44 (d, *J* = 13.4 Hz, 1H, Ar-CH_2_-Ar), 4.35 (d, *J* = 13.4 Hz, 1H, Ar-CH_2_-Ar), 3.95–4.07 (m, 1H (Ar-CH_2_-Ar) + 4H (-OCH_2_-)), 3.63–3.76 (m, 4H, -OCH_2_-), 3.16 (d, *J* = 13.4 Hz, 1H, Ar-CH_2_-Ar), 3.15 (d, *J* = 13.4 Hz, 2H, Ar-CH_2_-Ar), 1.93–2.03 (m, 4H, -CH_2_-CH_3_), 1.82–1.92 (m, 4H, -CH_2_-CH_3_), 1.10 (t, *J* = 7.3 Hz, 6H, -CH_3_), 0.91 (t, *J* = 7.3 Hz, 3H, -CH_3_), 0.89 (t, *J* = 7.3 Hz, 6H, -CH_3_). ^13^C NMR (CDCl_3_, 125 MHz, 298 K) *δ* 160.47, 157.98, 157.88, 155.17, 155.16, 139.31, 137.99, 137.01, 136.89, 133.47, 133.22, 132.92, 132.67, 128.84, 128.78, 128.51, 127.57, 127.41, 127.33, 127.24, 126.21, 125.01, 122.10 (2C), 121.82, 121.78, 109.33, 101.42, 91.1, 88.83, 76.94, 76.90, 76.58, 76.44, 55.43 (2C), 31.04, 30.97, 30.96, 28.11, 23.50 (2C), 22.99, 22.97, 10.79, 10.78, 9.86, 9.84 ppm. HRMS (ESI^+^) calcd for C_48_H_52_O_4_ 715.3757 [M + Na]^+^, found *m*/*z* 715.3764 [M + Na]^+^. IR (KBr) ν 2960, 2931, 2873, 1493, 1454, 1382, 1208, 1192, 1087, 1044, 965, 754, 689 cm^−1^.

##### Alkynylcalixarene **5c**

Method A: Calixarene **3** (0.209 mmol, 150 mg), 4-ethynylanisole (0.230 mmol, 30 µL), CuI (0.0209 mmol, 4 mg) and Pd(PPh_3_)_2_Cl_2_ (0.011 mmol, 7 mg) were used under standard conditions; the product was purified by column chromatography on silica gel (DCM/cyclohexane 1:1) to afford **5c** in 64% yield (97 mg) as a white solid.

Method B: Calixarene **3** (0.139 mmol, 106 mg), 4-ethynylanisole (0.556 mmol, 70 µL), CuI (0.028 mmol, 5 mg), piperidine (0.417 mmol, 40 µL) and Pd(PPh_3_)_4_ (0.0139 mmol, 16 mg) were used under standard conditions. The product **5c** (58 mg, 58%) was obtained by column chromatography on silica gel (DCM/cyclohexane 50:50) as a white solid.

Data for compound **5c**: M.p. = 146–149 °C. ^1^H NMR (CDCl_3_, 400 MHz, 298 K) *δ* 7.49 (d, 2H, *J* = 8.4 Hz, Ar-H) 7.22 (d, *J* = 7.7 Hz, 1H, Ar-H) 7.13 (d, *J* = 7.4 Hz, 2H, Ar-H) 7.10 (d, *J* = 7.8 Hz, 1H, Ar-H) 6.94 (t, *J* = 7.6 Hz, 1H, Ar-H) 6.88 (d, *J* = 8.4 Hz, 2H, Ar-H) 6.39 (d, *J* = 7.8 Hz, 1H, Ar-H), 6.29 (t, *J* = 7.6 Hz, 1H, Ar-H), 6.27 (t, *J* = 7.6 Hz, 1H, Ar-H), 6.18 (br d, *J* = 7.4 Hz, 2H, Ar-H), 6.17 (br d, *J* = 7.4 Hz, 1H, Ar-H), 4.50 (d, *J* = 13.4 Hz, 1H, Ar-CH_2_-Ar), 4.49 (d, *J* = 13.4 Hz, 1H, Ar-CH_2_-Ar), 4.48 (d, *J* = 13.4 Hz, 1H, Ar-CH_2_-Ar), 4.38 (d, *J* = 13.4 Hz, 1H, Ar-CH_2_-Ar), 4.12–4.02 (m, 4H, -OCH_2_-), 4.02 (d, *J* = 13.4 Hz, 1H, Ar-CH_2_-Ar), 3.83 (s, 3H, -OCH_3_), 3.79–3.64 (m, 4H, -OCH_2_-), 3.19 (d, *J* = 13.4 Hz, 3H, Ar-CH_2_-Ar), 2.09–1.96 (m, 4H, -CH_2_-CH_3_), 1.96–1.84 (m, 4H, -CH_2_-CH_3_), 1.13 (t, *J* = 7.4 Hz, 6H, -CH_3_), 0.93 (t, *J* = 7.4 Hz, 3H, -CH_3_), 0.92 (t, *J* = 7.4 Hz, 3H, -CH_3_). ^13^C NMR (CDCl_3_, 125 MHz, 298 K) *δ* 160.35, 158.97, 158.99, 156.10 (2C), 140.02, 138.43, 137.93, 137.80, 135.25, 134.01, 133.89, 133.78 (2C), 133.58, 129.69, 129.62, 129.29, 128.35, 128.19, 128.11, 128.08, 126.76, 123.12, 122.92, 122.89, 122.58, 116.60, 114.64 (2C), 91.78, 88.45, 77.49, 77.46, 77.12, 76.99, 55.70, 31.34, 31.27 (2C), 28.30, 23.79 (2C), 23.27, 23.23, 11.03, 11.01, 10.06, 10.04 ppm. HRMS (ESI^+^) calcd for C_49_H_54_O_5_ 745.3863 [M + Na]^+^, found *m*/*z* 775.3871 [M + Na]^+^. IR (KBr) ν 2960, 2932, 2920, 2874, 1511, 1454, 1249, 1208, 1192, 1105, 964, 830, 794, 775, 756 cm^−1^.

##### Alkynylcalixarene **5d**

Method A: Calixarene **3** (0.209 mmol, 150 mg), 1-ethynyl-4-nitrobenzene (0.230 mmol, 34 mg), CuI (0.0209 mmol, 4 mg) and Pd(PPh_3_)_2_Cl_2_ (0.011 mmol, 7 mg) were used under standard conditions; the product was purified by column chromatography on silica gel (DCM/cyclohexane 1:1) to afford **5d** in 27% yield (42 mg) as a yellow oil.

Method B: Calixarene **3** (0.097 mmol, 70 mg), 1-ethynyl-4-nitrobenzene (0.388 mmol, 62 mg), CuI (0.019 mmol, 4 mg), piperidine (0.291 mmol, 30 µL) and Pd(PPh_3_)_4_ (0.0097 mmol, 11 mg) were used under standard conditions. The product **5d** (28 mg, 39%) was obtained by column chromatography on silica gel (DCM/cyclohexane 50:50) as a yellow oil.

Data for compound **5d**: ^1^H NMR (CDCl_3_, 500 MHz, 298 K) *δ* 8.19 (d, *J* = 8.9 Hz, 2H, Ar-H), 7.63 (d, *J* = 8.9 Hz, 2H, Ar-H), 7.21 (d, *J* = 7.7 Hz, 1H, Ar-H), 7.08 (d, *J* = 7.7 Hz, 1H, Ar-H), 7.07 (d, *J* = 7.5 Hz, 1H, Ar-H), 6.89 (t, *J* = 7.5 Hz, 1H, Ar-H), 6.31 (dd, *J* = 7.7 Hz, *J* = 1.1 Hz, 1H, Ar-H), 6.26 (td, *J* = 7.5 Hz, *J* = 3.2 Hz, 2H, Ar-H), 6.19 (t, *J* = 6.2 Hz, 2H, Ar-H), 6.15 (d, *J* = 7.5 Hz, 1H, Ar-H), 4.49 (d, *J* = 13.3 Hz, 2H, Ar-CH_2_-Ar) 4.46 (dd, *J* = 13.3 Hz, *J* = 3.3 Hz 2H, Ar-CH_2_-Ar), 4.41 (d, *J* =13.3 Hz, 1H, Ar-CH_2_-Ar), 4.08–3.96 (m, 4H, -O-CH_2_-), 3.90 (d, 1H, *J* = 13.3 Hz, Ar-CH_2_-Ar), 3.78–3.65 (m, 4H, -O-CH_2_-), 3.19 (d, *J* = 13.2 Hz, 1H, Ar-CH_2_-Ar), 3.16 (d, *J* = 13.2 Hz, 1H, Ar-CH_2_-Ar), 3.73 (m, 2H, -O-CH_2_-), 1.99 (sextet, *J* = 7.7 Hz, 4H, -CH_2_-CH_3_), 1.89 (sextet, *J* = 7.7 Hz, 4H, -CH_2_-CH_3_), 1.11 (t, *J* = 7.5 Hz, 3H, -CH_3_), 1.10 (t, *J* = 7.5 Hz, 3H, -CH_3_), 0.92 (t, *J* = 7.5 Hz, 3H, -CH_3_), 0.91 (t, *J* = 7.5 Hz, 3H, -CH_3_).^13^C NMR (CDCl_3_, 125 MHz, 298 K) *δ* 158.02, 157.78, 155.24 (2C), 146.70, 139.56, 139.05, 136.87, 136.78, 133.62, 133.54, 132.59, 132.47, 132.10 (2C), 130.73, 128.80 (2C), 128.72, 127.71, 127.67, 127.21, 127.16, 126.46, 123.60 (2C), 122.11, 122.10, 121.85, 120.80, 95.05, 89.52, 76.97, 76.92, 76.68, 76.46, 31.10, 30.97 (2C), 28.20, 23.48, 23.47, 23.02, 22.98, 10.77, 10.76, 9.86, 9.85 ppm. HRMS (ESI^+^) calcd for C_48_H_51_NO_6_ 760.3609 [M + Na]^+^, found *m*/*z* 760.3607 [M + Na]^+^. IR (KBr) ν 2960, 2929, 2874, 1589, 1454, 1382, 1340, 1208, 1192, 1087, 1004, 966, 756 cm^−1^.

##### Alkynylcalixarene **5e**

Method A: Calixarene **3** (0.280 mmol, 205 mg), 3-ethynylpyridine (0.421 mmol, 40 µL), CuI (0.028 mmol, 5 mg) and Pd(PPh_3_)_2_Cl_2_ (0.014 mmol, 10 mg) were used under standard conditions; the product was purified by column chromatography on silica gel (DCM/cyclohexane 2:1) to afford **5e** in 33% yield (64 mg) as a yellow oil.

Method B: Calixarene **3** (0.280 mmol, 204 mg), 3-ethynylpyridine (0.88 mmol, 90 µL), CuI (0.056 mmol, 11 mg), piperidine (0.841 mmol, 80 µL) and Pd(PPh_3_)_4_ (0.028 mmol, 32 mg) were used under standard conditions. The product **5e** (91 mg, 47%) was obtained by column chromatography on silica gel (DCM/cyclohexane 2:1) as a yellow oil.

Data for compound **5e**: ^1^H NMR (CDCl_3_, 500 MHz, 298 K) *δ* 8.61 (br s, 1H, Ar-H) 7.64 (td, *J* = 7.7 Hz, *J* = 1.5 Hz, 1H, Ar-H) 7.49 (d, *J* = 7.8 Hz, 1H, Ar-H) 7.27 (d, *J* = 7.8 Hz, 1H, Ar-H) 7.20 (dd, *J* = 7.8 Hz, *J* = 4.8 Hz, 1H, Ar-H) 7.08 (d, *J* = 7.4 Hz, 3H, Ar-H) 6.89 (t, *J* = 7.4 Hz, 1H, Ar-H), 6.33 (d, *J* = 7.3 Hz, 1H, Ar-H), 6.21–6.28 (m, 2H, Ar-H), 6.10–6.19 (m, 3H, Ar-H), 4.48 (d, *J* = 13.0 Hz, 1H, Ar-CH_2_-Ar), 4.46 (d, *J* = 13.0 Hz, 1H, Ar-CH_2_-Ar), 4.45 (d, *J* = 13.0 Hz, 1H, Ar-CH_2_-Ar), 4.38 (d, *J* = 13.0 Hz, 1H, Ar-CH_2_-Ar), 3.94–4.10 (m, 1H (Ar-CH_2_-Ar) + 4H (-OCH_2_-)), 3.62–3.79 (m, 4H, -OCH_2_-), 3.18 (d, 1H, *J* = 13.0 Hz, 1H, Ar-CH_2_-Ar), 3.15 (d, 1H, *J* = 13.0 Hz, 2H, Ar-CH_2_-Ar), 1.93–2.03 (m, 4H, -CH_2_-CH_3_), 1.84–1.92 (m, 4H, -CH_2_-CH_3_), 1.10 (t, *J* = 7.3 Hz, 6H, -CH_3_), 0.91 (t, *J* = 7.3 Hz, 6H, -CH_3_), 0.90 (t, *J* = 7.3 Hz, 6H, -CH_3_).^13^C NMR (CDCl_3_, 125 MHz, 298 K) *δ* 157.98, 157.89, 155.19, 150.02, 143.88, 139.80, 138.70, 137.02, 136.90, 136.04, 133.50, 133.24, 132.79, 132.57, 128.86, 128.80, 128.61, 127.62, 127.45, 127.38, 127.26, 126.70, 122.46, 122.14, 122.12, 121.80, 121.04, 90.56, 89.46, 76.97, 76.90, 76.64, 76.47, 31.10, 31.00, 30.98, 23.51 (2C), 23.01, 23.99, 10.81, 10.79, 9.87, 9.86 ppm. HRMS (ESI^+^) calcd for C_47_H_51_NO_4_ 716.3710 [M + Na]^+^, found *m*/*z* 716.3710 [M + Na]^+^. IR (KBr) ν 2960, 2931, 2873, 1580, 1455, 1383, 1209, 1192, 1087, 1066, 1005, 956, 756 cm^−1^.

##### Alkynylcalixarene **5f**

Method A: Calixarene **3** (0.349 mmol, 250 mg), trimethylsilylacetylene (0.418 mmol, 60 µL), CuI (0.035 mmol, 7 mg) and Pd(PPh_3_)_2_Cl_2_ (0.017 mmol, 12 mg) were used under standard conditions; the product was purified by column chromatography on silica gel (DCM/cyclohexane 1:1) to afford **5f** in 83% yield (199 mg) as a yellow oil.

Method B: Calixarene **3** (0.138 mmol, 104 mg), trimethylsilylacetylene (0.552 mmol, 78 µL), CuI (0.028 mmol, 5 mg), piperidine (0.417 mmol, 40 µL) and Pd(PPh_3_)_4_ (0.014 mmol, 16 mg) were used under standard conditions. The product **5f** (71 mg, 75%) was obtained by column chromatography on silica gel (DCM/cyclohexane 1:1) as a yellow oil.

Data for compound **5f***:* ^1^H NMR (CDCl_3_, 500 MHz, 298 K) *δ* 7.14 (d, 1H, *J* = 7.8 Hz, Ar-H) 7.10 (m, 2H, Ar-H) 7.03 (d, *J* = 7.8 Hz, 2H, Ar-H) 6.91 (t, *J* = 7.4 Hz, 1H, Ar-H) 6.27 (dd, *J* = 7.7 Hz, *J* = 1.7 Hz, 1H, Ar-H) 6.25 (t, *J* = 7.4 Hz, 1H, Ar-H) 6.20 (t, *J* = 7.4 Hz, 1H, Ar-H), 6.15–6.10 (m, 2H, Ar-H), 6.07 (dd, *J* = 7.4 Hz, *J* = 1.7 Hz, 1H, Ar-H), 4.43 (d, *J* = 13.5 Hz, 1H, Ar-CH_2_-Ar), 4.44 (d, *J* = 13.5 Hz, 2H, Ar-CH_2_-Ar), 4.28 (d, *J* = 13.4 Hz, 1H, Ar-CH_2_-Ar), 4.04–3.95 (m, 4H, -OCH_2_), 4.48 (d, *J* = 13.4 Hz, 1H, Ar-CH_2_-Ar), 4.38 (d, *J* = 13.4 Hz, 1H, Ar-CH_2_-Ar), 4.12–4.02 (m, 4H, -OCH_2_-), 3.90 (d, *J* = 13.4 Hz, 1H, Ar-CH_2_-Ar), 3.76–3.60 (m, 4H, -OCH_2_-), 3.14 (d, *J* = 13.4 Hz, 2H, Ar-CH_2_-Ar), 3.13 (d, *J* = 13.4 Hz, 1H, Ar-CH_2_-Ar), 2.02–1.90 (m, 4H, -CH_2_-CH_3_), 1.91–1.80 (m, 4H, -CH_2_-CH_3_), 1.09 (t, *J* = 7.4 Hz, 3H, -CH_3_), 1.08 (t, *J* = 7.4 Hz, 3H, -CH_3_), 0.87 (t, *J* = 7.4 Hz, 6H, -CH_3_), 0.21 (s, 9H, -CH_3_) ^13^C NMR (CDCl_3_, 100 MHz, 298 K) *δ* 158.40, 158.36, 155.55, 155.50, 140.07, 138.56, 137.43, 137.35, 133.70, 133.47, 133.28, 132.93, 129.22, 129.12, 128.71, 127.84, 127.65, 127.54, 127.50, 126.79, 122.41, 122.36, 122.20, 122.06, 105.20, 96.13, 76.98 (2C), 76.57, 76.49, 30.82, 30.77, 30.74, 27.64, 23.28 (2C), 22.71 (2C), 10.52 (2C), 9.51 (2C), 0.28 (3C).ppm. HRMS (ESI^+^) calcd for C_45_H_56_SiO_4_ 711.3840 [M + Na]^+^, found *m*/*z* 711.3847 [M + Na]^+^. IR (KBr) ν 2960, 2932, 2874, 1455, 1383, 1247, 1207, 1192, 1088, 1006, 966, 841, 756 cm^−1^.

#### 3.2.2. Preparation of Bridged Calixarene **7b**

Diphenyl diselenide (0.609 mmol, 190 mg) and FeCl_3_ (0.752 mmol, 122 mg) were dissolved in 4 mL of dry dichloromethane in a Schlenk flask under an argon atmosphere. The resulting solution was stirred for 15 min at room temperature. After this time, the solution changed color to brown-red. Calixarene **5b** (0.301 mmol, 209 mg) dissolved in 2 mL of dry dichloromethane was added to the above solution, and the resulting mixture was heated at 30 °C for 4 h. Dichloromethane (20 mL) was then added to the reaction mixture, the organic layer was washed with a saturated solution of NH_4_Cl, dried with MgSO_4_ and concentrated under vacuum. The residue was purified by column chromatography on silica gel (DCM/cyclohexane 1:1) to provide the respective product **7b** in 20% yield (51 mg) as a yellow solid.

Data for compound **7b**: M.p. = 117–120 °C. ^1^H NMR (CDCl_3_, 400 MHz, 298 K) *δ* 7.24 (dd, *J* = 8.0 Hz, *J* = 1.8 Hz, 2H, Ar-H), 7.19 (dd, *J* = 8.0 Hz, *J* = 1.8 Hz, 2H, Ar-H), 7.03–6.95 (m, 7H, Ar-H) 6.93 (d, *J* = 7.3 Hz, 1H, Ar-H), 6.89 (d, *J* = 8.0 Hz, 1H, Ar-H), 6.87 (dd, *J* = 8.0 Hz, *J* = 1.5 Hz, 1H, Ar-H), 6.82 (dd, *J* = 7.8 Hz, *J* = 1.5 Hz, 1H, Ar-H), 6.75 (d, *J* = 9.0 Hz, 1H, Ar-H), 6.73 (t, *J* = 7.6 Hz, 1H, Ar-H), 6.61 (t, *J* = 7.4 Hz, 1H, Ar-H), 6.43 (d, *J* = 7.8 Hz, 1H, Ar-H), 5.98 (d, *J* = 7.8 Hz, 1H, Ar-H), 4.70 (d, *J* = 13.4 Hz, 1H, Ar-CH_2_-Ar), 4.50 (d, *J* = 12.5 Hz, 1H, Ar-CH_2_-Ar), 4.41 (d, *J* = 12.0 Hz, 1H, Ar-CH_2_-Ar), 4.33 (d, *J* = 12.5 Hz, 1H, Ar-CH_2_-Ar), 4.04–3.79 (m, 6H, -OCH_2_-), 3.64–3.54 (m, 2H, -OCH_2_-), 3.28 (d, *J* = 13.2 Hz, 1H, Ar-CH_2_-Ar), 3.27 (d, *J* = 12.5 Hz, 1H, Ar-CH_2_-Ar), 3.11 (d, *J* = 12.5 Hz, 1H, Ar-CH_2_-Ar), 3.07 (d, *J* = 12.5 Hz, 1H, Ar-CH_2_-Ar), 2.21 (sextet, *J* = 7.7 Hz, 2H, -CH_2_-CH_3_), 2.13–2.01 (m, 2H, -CH_2_-CH_3_), 2.01–1.83 (m, 4H, -CH_2_-CH_3_), 1.20 (t, *J* = 7.4 Hz, 3H, -CH_3_), 1.19 (t, *J* = 7.4 Hz, 3H, -CH_3_), 1.00 (t, *J* = 7.4 Hz, 3H, -CH_3_), 0.99 (t, *J* = 7.4 Hz, 3H, -CH_3_). ^13^C NMR (CDCl_3_, 100 MHz, 298 K) *δ* 156.32, 155.80, 154.80, 154.77, 140.82, 140.37, 140.36, 140.08, 136.54, 136.23, 135.04, 134.42, 134.18, 132.98, 132.71, 132.70, 132.21, 131.16, 130.97 (2C), 130.39, 129.27, 128.86, 128.41 (2C), 128.09, 127.40, 127.37 (2C), 126.89, 126.58, 126.48, 126.44, 123.27, 123.05, 123.01, 122.94, 77.48, 77.45, 76.99, 76.70, 31.79, 31.56, 29.19, 24.82, 23.77, 23.73, 23.12, 22.99, 11.10 (2C), 10.29, 10.15 ppm. HRMS (ESI^+^) calcd for C_54_H_56_SeO_4_ 871.3236 [M + Na]^+^, found *m*/*z* 871.3246 [M + Na]^+^. IR (KBr) ν 2959, 2924, 2872, 1455, 1250, 1208, 1066, 1007, 960, 734, 690 cm^−1^.

#### 3.2.3. Preparation of Bridged Calixarene **8b**

Diphenyl diselenide (0.28 mmol, 90 mg) and FeCl_3_ (0.351 mmol, 57 mg) were dissolved in 3 mL of dry DCE in a Schlenk flask under an argon atmosphere, and the resulting solution was stirred for 15 min at room temperature. After this time, the color of the solution turned to brown-red. Calixarene **5b** (0.144 mmol, 100 mg) dissolved in 1 mL of dry DCE was added, and the reaction mixture was heated to 85 °C for 4 h. The eluent was then evaporated under reduced pressure, dichloromethane (20 mL) was added to the crude product, washed with a saturated solution of NH_4_Cl, dried with MgSO_4_ and then concentrated under vacuum. The residue was purified by preparative thin layer chromatography on silica gel (DCM/cyclohexane 1:1) to provide the methylene bridged product **8b** in 10% yield (12 mg) as a white solid.

Data for compound **8b**: M.p. = 101–104 °C. ^1^H NMR (CDCl_3_, 400 MHz, 298 K) *δ* 8.38 (s, 1H, -OH), 8.25 (s, 1H, -OH), 7.45 (d, *J* = 7.9 Hz, 2H, Ar-H), 7.39 (d, *J* = 1.4 Hz, 1H, Ar-H), 7.34 (t, *J* = 7.0 Hz, 3H, Ar-H), 7.30–7.11 (m, 10H, Ar-H) 7.07 (d, *J* = 7.3 Hz, 1H, Ar-H), 6.87 (d, *J* = 7.3 Hz, 1H, Ar-H), 6.83 (d, *J* = 7.3 Hz, 2H, Ar-H), 6.74 (d, *J* = 7.5 Hz, 1H, Ar-H), 6.73 (t, *J* = 7.4 Hz, 1H, Ar-H), 6.59 (t, *J* = 7.7 Hz, 1H, Ar-H), 6.50 (d, *J* = 7.2 Hz, 1H, Ar-H), 5.49 (s, 1H, Ar-CH(R)-Ar), 4.51 (d, *J* = 12.4 Hz, 1H, Ar-CH_2_-Ar), 4.43 (d, *J* = 12.5 Hz, 1H, Ar-CH_2_-Ar), 4.18–4.04 (m, 2H, -OCH_2_-), 4.03–3.90 (m, 2H, -OCH_2_-), 3.43 (d, *J* = 13.0 Hz, 1H, Ar-CH_2_-Ar), 3.35 (d, *J* = 13.0 Hz, 1H, Ar-CH_2_-Ar), 3.26 (d, *J* = 12.4 Hz, 1H, Ar-CH_2_-Ar), 2.19–1.96 (m, 4H, -CH_2_-CH_3_), 1.37 (t, *J* = 7.3 Hz, 3H, -CH_3_), 1.30 (t, *J* = 7.4 Hz, 3H, -CH_3_). ^13^C NMR (CDCl_3_, 125 MHz, 298 K) *δ* 154.14, 153.59, 153.13, 152.32, 151.24, 144.49, 135.99, 134.72, 134.00, 133.82, 132.04, 131.70, 130.81, 130.35, 130.24 (2C), 130.24, 130.06, 130.02, 129.72 (2C), 129.46 (2C), 129.32 (2C), 129.21, 129.17, 128.68, 128.56, 128.23 (2C), 128.01, 127.57, 126.25, 125.94, 125.76, 125.50, 125.22, 116.72, 113.61, 78.29, 78.21, 50.51, 32.11, 31.45, 30.09, 23.88, 23.69, 11.18, 11.06 ppm. HRMS (ESI^+^) calcd for C_54_H_48_Se_2_O_4_ 943.1775 [M + Na]^+^, found *m*/*z* 943.1785 [M + Na]^+^.

#### 3.2.4. Preparation of Bridged Calixarenes **9c** and **10c**

Diphenyl diselenide (0.170 mmol, 53 mg) and FeCl_3_ (0.213 mmol, 34 mg) were dissolved in 3 mL of dry dichloromethane in a Schlenk flask under an argon atmosphere. The resulting solution was stirred for 15 min at room temperature. After this time, the solution changed color to brown-red. Calixarene **5c** (0.065 mmol, 85 mg) dissolved in 1 mL of dry dichloromethane was added, and the reaction mixture was heated at 30 °C for 4 h. Dichloromethane (20 mL) was added to the mixture, the organic layer was washed with a saturated solution of NH_4_Cl, dried over MgSO_4_, and concentrated under vacuum. The residue was purified by column chromatography on a silica gel (DCM/cyclohexane 1:1) to provide the respective products **9c** in 37% yield (27 mg) and **10c** in 15% yield (11 mg) as yellow oils.

Data for compound **9c**: ^1^H NMR (CDCl_3_, 400 MHz, 298 K) *δ* 7.41 (dd, *J* = 7.4 Hz, *J* = 1.5 Hz, 2H, Ar-H), 7.31 (d, *J* = 8.4 Hz, 2H, Ar-H), 7.23–7.15 (m, 4H, Ar-H) 7.13 (dd, *J* = 7.5 Hz, *J* = 1.4 Hz, 1H, Ar-H), 7.07 (d, *J* = 7.5 Hz, 1H, Ar-H), 6.95 (t, *J* = 7.3 Hz, 1H, Ar-H), 6.87 (d, *J* = 7.4 Hz, 1H, Ar-H), 6.77 (d, *J* = 8.9 Hz, 2H, Ar-H), 6.20 (t, *J* = 7.4 Hz, 1H, Ar-H), 6.14–6.07 (m, 3H, Ar-H), 6.02 (dd, *J* = 7.5 Hz, *J* = 1.3 Hz, 1H, Ar-H), 5.80 (dd, *J* = 7.5 Hz, *J* = 1.5 Hz, 1H, Ar-H), 5.70 (s, 1H, Ar-CH(R)-Ar), 4.49 (d, *J* = 13.3 Hz, 1H, Ar-CH_2_-Ar), 4.44 (d, *J* = 13.3 Hz, 2H, Ar-CH_2_-Ar), 4.34 (td, *J* = 11.0 Hz, *J* = 5.3 Hz, 1H, -OCH_2_-), 4.23–4.10 (m, 2H, -OCH_2_-), 4.04–3.94 (m, 1H, -OCH_2_-), 3.74 (s, 3H, -OCH_3_), 3.71–3.64 (m, 3H, -OCH_2_-), 3.54–3.46 (m, 1H, -OCH_2_-), 3.19 (d, *J* = 13.3 Hz, 1H, Ar-CH_2_-Ar), 3.15 (d, *J* = 13.3 Hz, 1H, Ar-CH_2_-Ar), 3.13 (d, *J* = 13.3 Hz, 1H, Ar-CH_2_-Ar), 2.04–1.81 (m, 8H, -CH_2_-CH_3_), 1.14 (t, *J* = 7.5 Hz, 3H, -CH_3_), 1.10 (t, *J* = 7.4 Hz, 3H, -CH_3_), 0.91 (t, *J* = 7.5 Hz, 3H, -CH_3_), 0.89 (t, *J* = 7.4 Hz, 3H, -CH_3_). We cannot acquire reliable ^13^C NMR spectra due to degradation of compound. HRMS (ESI^+^) calcd for C_55_H_58_SeO_5_ 901.3341 [M + Na]^+^, found *m*/*z* 901.3345 [M + Na]^+^.

Data for compound **10c**: ^1^H NMR (CDCl_3_, 400 MHz, 298 K) *δ* 7.40 (dd, *J* = 7.4 Hz, *J* = 1.5 Hz, 2H, Ar-H), 7.37 (d, *J* = 8.6 Hz, 2H, Ar-H), 7.22–7.14 (m, 3H, Ar-H), 7.10 (d, *J* = 7.6 Hz, 3H, Ar-H), 6.93 (d, *J* = 7.6 Hz, 1H, Ar-H), 6.81 (d, *J* = 7.6 Hz, 1H, Ar-H), 6.77 (d, *J* = 8.6 Hz, 2H, Ar-H), 6.46–6.34 (m, 3H, Ar-H), 6.27 (~dd, *J* = 7.6 Hz, *J* = 1.5 Hz, 1H, Ar-H), 6.22 (t, *J* = 7.6 Hz, 1H, Ar-H), 5.98 (dd, *J* = 7.6 Hz, *J* = 1.5 Hz, 1H, Ar-H), 5.74 (s, 1H, Ar-CH(R)-Ar), 4.42 (d, *J* = 13.3 Hz, 1H, Ar-CH_2_-Ar), 4.40 (d, *J* = 13.3 Hz, 1H, Ar-CH_2_-Ar), 4.37 (d, *J* = 13.3 Hz, 1H, Ar-CH_2_-Ar), 4.28–4.19 (m, 1H, -OCH_2_-), 4.00–3.92 (m, 1H, -OCH_2_-), 3.77–3.72 (m, 3H + 3H, -OCH_2_-, -OCH_3_), 3.65–3.59 (m, 1H, -OCH_2_-), 3.33 (d, *J* = 13.3 Hz, 1H, Ar-CH_2_-Ar), 3.23 (d, *J* = 13.3 Hz, 1H, Ar-CH_2_-Ar), 3.22 (d, *J* = 13.3 Hz, 1H, Ar-CH_2_-Ar), 2.26–2.20 (m, 1H, -CH_2_-CH_3_), 2.15–2.07 (m, 1H, -CH_2_-CH_3_), 1.97–1.84 (m, 4H, -CH_2_-CH_3_), 1.15 (t, *J* = 7.3 Hz, 3H, -CH_3_), 1.10 (t, *J* = 7.3 Hz, 3H, -CH_3_), 0.93 (t, *J* = 7.4 Hz, 3H, -CH_3_). We cannot acquire ^13^C NMR spectra due to degradation of compound. HRMS (ESI^+^) calcd for C_52_H_52_SeO_5_ 859.2881 [M + Na]^+^, found *m*/*z* 859.2881 [M + Na]^+^.

#### 3.2.5. Electrochemical Experiments

##### Batch Electrolysis Setup

The electrolysis was performed in a 20 mL undivided cell filled with 15 mL of MeCN (HPLC gradient grade ≥99.9%, Merck), containing 0.02 M LiClO_4_·H_2_O (BDH Chemicals Ltd., Poole, England) used as a supporting electrolyte. Additionally, the solution contained compound **5b** with 0.6 equiv. of Ph_2_Se_2_. As working electrodes, two different materials were tested: Pt sheet (5 mm × 30 mm), or glassy carbon (GC, diameter 3 mm). In the case of using GC as the working electrode, GC was conducted by Motorcontroller for Rotator, and 100 RPM rotation speed was used. As the reference electrode, a saturated calomel electrode (SCE) separated from the investigated sample by a salt-bridge filled by the blank (MeCN electrolyte solution) was used, and as the counter (auxiliary) electrode, a Pt sheet (5 mm × 30 mm) or GC (diameter 3 mm) were applied. The reaction mixture was stirred and electrolyzed at room temperature during (a) constant current conditions (10 mA was applied) (b) controlled-potential electrolysis (potential was set to +1.5 V). In the cases when electrolysis was performed under inert atmosphere, oxygen was removed from the solution by passing a stream of argon (99.998%, Messer, Bad Soden, Germany). Measurements were carried out using the computer-driven digital potentiostat PGSTAT204 (Autolab-Metrohm, Herisau, Switzerland) controlled by software NOVA 1.11.

##### Attempted Electrolytic Cyclization of **5b**

Calixarene **5b** (25 mg, 0.036 mmol) with 0.6 equiv. of diphenyl diselenide (6.76 mg, 0.022 mmol) was dissolved in 15 mL of solution of a supporting electrolyte (0.02 M LiClO_4_·H_2_O). The solution was poured into an undivided cell, and then the Pt plate (used as working electrode), SCE (reference electrode), and Pt plate (auxiliary electrode) were placed in solution (Figure S79). The reaction mixture was stirred by a magnetic bar at room temperature during constant current (set to 10 mA) under aerobic condition. The progress of the reaction was monitored by TLC until the full conversion of **5b** was detected (3.5 h).

After completion, the crude reaction mixture was collected and evaporated to dryness. The solid was partitioned between DCM and water; the organic layer was washed with water and dried over MgSO_4_. Crude evaporated product was purified using preparative TLC on silica gel (DCM/cyclohexane 1:1) to provide product 11 (19 mg, 73%) as a white solid.

Data for compound **11**: M.p. = 137–141 °C. ^1^H NMR (CDCl_3_, 400 MHz, 298 K) *δ* 7.92 (d, *J* = 7.4 Hz, 2H, Ar-H), 7.65 (t, *J* = 7.8 Hz, 1H, Ar-H), 7.51 (t, *J* = 7.1 Hz, 2H, Ar-H), 7.15 (d, *J* = 8.2 Hz, 1H, Ar-H), 6.97 (d, *J* = 7.5 Hz, 1H, Ar-H), 6.91 (t, *J* = 7.1 Hz, 2H, Ar-H), 6.70 (t, *J* = 7.0 Hz, 1H, Ar-H), 6.39–6.27 (m, 5H, Ar-H), 6.23 (d, *J* = 7.4 Hz, 1H, Ar-H), 4.55 (d, *J* = 13.4 Hz, 1H, Ar-CH_2_-Ar), 4.52 (d, *J* = 13.4 Hz, 1H, Ar-CH_2_-Ar), 4.45 (d, *J* = 13.4 Hz, 1H, Ar-CH_2_-Ar), 4.44 (d, *J* = 13.4 Hz, 1H, Ar-CH_2_-Ar), 4.41 (d, *J* = 13.4 Hz, 1H, Ar-CH_2_-Ar), 4.04–3.91 (m, 4H, -OCH_2_-), 3.82–3.65 (m, 4H, -OCH_2_-), 3.20 (d, *J* = 13.4 Hz, 1H, Ar-CH_2_-Ar), 3.17 (d, *J* = 13.4 Hz, 1H, Ar-CH_2_-Ar), 3.15 (d, *J* = 13.4 Hz, 1H, Ar-CH_2_-Ar), 2.06–1.82 (m, 8H, -CH_2_-CH_3_), 1.06 (t, *J* = 7.5 Hz, 3H, -CH_3_), 1.05 (t, *J* = 7.4 Hz, 3H, -CH_3_), 0.95 (t, *J* = 7.5 Hz, 3H, -CH_3_), 0.93 (t, *J* = 7.4 Hz, 3H, -CH_3_). ^13^C NMR (CDCl_3_, 100 MHz, 298 K) *δ* 197.92, 195.89, 160.23, 158.43, 156.69, 156.58, 144.17, 140.08, 137.42, 137.02, 135.35, 135.17, 134.68, 134.09, 134.01, 132.94, 132.51, 130.79 (2C), 129.71 (2C), 129.56, 129.49, 129.42, 129.28, 129.07, 129.02, 128.92, 128.83, 128.72, 128.39, 128.22, 128.12, 122.97, 122.76, 122.59, 77.51, 77.49, 77.09, 77.05, 31.47, 31.37, 31.29, 25.94, 23.64, 23.48, 23.33, 23.30, 10.81, 10.76, 10.18, 10.12 ppm. HRMS (ESI^+^) calcd for C_48_H_52_O_6_ 747.3656 [M + Na]^+^, found *m*/*z* 747.3662 [M + Na]^+^. IR (KBr) ν 2961, 2924, 2872, 1674, 1665, 1586, 1454, 1384, 1290, 1245, 1228, 1105, 964, 785, 756, 719 cm^−1^.

##### Cyclic Voltammetry

Cyclic voltammetry measurements (with scan rates 100, 200, and 500 mV·s^−1^) were performed in MeCN (HPLC gradient grade ≥99.9%, Merck) using 0.02 M LiClO_4_·H_2_O (BDH Chemicals Ltd., Poole, England) as the supporting electrolyte (Figures S77 and S78). Due to low conductivity, the three-electrode systems were applied for all measurements. As the working electrode, a glassy carbon electrode (diameter 3 mm) or Pt disk electrode (diameter 1 mm) was utilized. As the reference electrode, a saturated calomel electrode (SCE) separated from the investigated sample by a bridge filled by the blank (MeCN-electrolyte solution) was applied, and as the auxiliary electrode, a Pt sheet was used. All those experiments were standardly carried out in an undivided 20 mL cell (using 10 mL of the corresponding solution), and before measurements, the solutions were de-aerated by argon (99.998%, Messer). Measurements were carried out using the computer-driven digital potentiostat PGSTAT101 (Autolab-Metrohm) controlled by software NOVA 1.11.

### 3.3. X-ray Measurements

#### 3.3.1. Crystallographic Data for **7b**

M = 848 g·mol^−1^, triclinic system, space group *P-1*, *a* = 11.6678 (4) Å, *b* = 17.7287(5) Å, *c* = 21.7145(6) Å, *α* = 82.4177(12)°, *β* = 86.9096(13)°, *γ* = 88.6006(13)°, *Z* = 4, *V* = 4445.3(2) Å^3^, *D_c_* = 1.267 g·cm^−3^, *μ*(Cu-Kα) = 1.494 mm^−1^, crystal dimensions of 0.077 × 0.077 × 0.158 mm. Data were collected at 180 (2) K on a Bruker D8 Venture Photon II 7 diffractometer with Incoatec microfocus sealed tube Cu-Kα radiation. The data were integrated, scaled and corrected for absorption using Apex4 [33]. The structure was solved by SIR92 [34] and anisotropically refined by full matrix least squares on *F* squared using the CRYSTALS [35] to final value *R* = 0.048 and *wR* = 0.124 using 1486 independent reflections (*θ_max_* = 72.2°), 1082 parameters and 42 restrains. The hydrogen atoms bonded to carbon atoms were placed in calculated positions and refined with riding constraints, while hydrogen atoms bonded to oxygen were refined using soft restraints. The disordered functional groups positions were found in difference electron density maps and refined with restrained geometry. MCE [36] was used for the visualization of electron density maps. The occupancy of disordered functional groups was constrained to full. The structure was deposited into Cambridge Structural Database under number CCDC 2332078. The full numbering scheme can be found in Supporting information Figures S74 and S75.

#### 3.3.2. Crystallographic Data for **8b**

M = 1003.82 g·mol^−1^, monoclinic system, space group *P*2_1_/*n*, *a* = 13.3272(4) Å, *b* = 13.0362(4) Å, *c* = 27.1526(7) Å, *β* = 94.7510(10)°, *Z* = 4, *V* = 4701.2(2) Å^3^, *D_c_* = 1.418 g·cm^−3^, *μ*(Cu-Kα) = 3.38 mm^−1^, crystal dimensions of 0.048 × 0.126 × 0.426 mm. Data were collected at 180 (2) K on a Bruker D8 Venture Photon II 7 diffractometer with Incoatec microfocus sealed tube Cu-Kα radiation. The data were integrated, scaled and corrected for absorption using Apex4 [33]. The structure was solved by SIR92 [34] and anisotropically refined by full matrix least squares on *F* squared using the CRYSTALS [35] to final value *R* = 0.060 and *wR* = 0.178 using 9241 independent reflections (*θ_max_* = 72.1°), 577 parameters and 0 restrains. The hydrogen atoms bonded to carbon atoms were placed in calculated positions and refined with riding constraints, while hydrogen atoms bonded to oxygen were first refined using soft restraints, then refined with riding constraints. The disordered solvent positions were found in difference electron density maps. MCE [36] was used for the visualization of electron density maps. The occupancy of disordered functional groups was first constrained to full, later fixed at values of 2/3 and 1/3, respectively. The structure was deposited into Cambridge Structural Database under number CCDC 2332079. The full numbering scheme can be found in Supporting information Figure S76.

## 4. Conclusions

A Sonogashira coupling of *meta*-iodocalix[4]arene with various terminal acetylenes confirmed that the *meta* position of calixarene is well addressable, and that both thermal and microwave protocols lead to good yields of the corresponding alkynylcalixarenes. Alkynes thus obtained were subjected to the FeCl_3_/Ph_2_Se_2_-promoted electrophilic closure. It turns out that the calix[4]arenes give completely different bridging products than those described for the non-macrocyclic starting compounds. This can be demonstrated not only by the isolation of products with a six-membered ring (*6-exo-dig*), but mainly by the smooth formation of the *5-endo-dig* cyclization, which has never been observed in the aliphatic series. An attempt at electrocyclization led to a high yield of the 1,2-diketone, formed by oxidation of the starting alkyne. The structures of the unexpected products were unequivocally established by X-ray analysis, and clearly demonstrate how the preorganized macrocyclic skeleton favors a completely different regioselectivity of cyclization reactions compared to common aliphatic derivatives.

## Figures and Tables

**Table 1:** The yields of the Sonogashira cross-coupling of calixarene **3** with acetylenes **4a**–**4f**.

Product	Method A ^(a)^	Method B ^(b)^
**5a**	89%	74%
**5b**	96%	62%
**5c**	64%	58%
**5d**	27%	39%
**5e**	33%	47%
**5f**	83%	75%

^(a)^ Method A: Pd(PPh_3_)_2_Cl_2_/CuI in piperidine at 100 °C, stirring for 3 days. ^(b)^ Method B: Pd(PPh_3_)_4_/CuI, piperidine in THF at 70 °C for 4 h, microwave oven.

## Data Availability

All experimental data are provided in the Supplementary Materials.

## Supplementary Materials

The following supporting information can be downloaded at: https://www.mdpi.com/article/10.3390/molecules29061237/s1, Spectral characterization of all new compounds (^1^H NMR, ^13^C NMR, HRMS, IR), VT NMR experiments, X-ray structures and electrochemical experiments.
