# Supplementary material for: Upper Rim-Bridged Calix[4]arenes via Cyclization of meta Alkynyl Intermediates with Diphenyl Diselenide
Source: Molecules. 2024 Mar 11;29(6):1237. doi: 10.3390/molecules29061237 (PMC10975271; doi:10.3390/molecules29061237)
Supplement: Supplementary file 1 [file molecules-29-01237-s001.zip › molecules-2901732-supplementary.pdf]

Supporting Information

Upper Rim-Bridged Calix[4]arenes via Cyclization of *meta*- Alkynyl Intermediates with Diphenyl Diselenide

Anastasia Surina,[1] Karolína Salvadori,[2,3] Matěj Poupě,[1] Jan Čejka,[4] Ludmila Šimková[2] and Pavel Lhoták[1,*]

[1] Department of Organic Chemistry, University of Chemistry and Technology, Prague (UCTP), Technická 5, 166 28 Prague 6, Czech Republic.
[2] J. Heyrovský Institute of Physical Chemistry of Czech Academy of Sciences v.v.i., Dolejškova 2155/3, 182 23 Prague 8, Czech Republic.
[3] Department of Physical Chemistry, UCTP, Technická 5, 166 28 Prague 6, Czech Republic.
[4] Department of Solid State Chemistry, UCTP, Technická 5, 166 28 Prague 6, Czech Republic.

* Correspondence: lhotakp@vscht.cz; Tel.: +420-440225055

Table of Contents

1. Spectral characterization of compounds 2
2. Crystallographic data 37
3. Electrochemistry 39

1. Spectral characterization of compounds

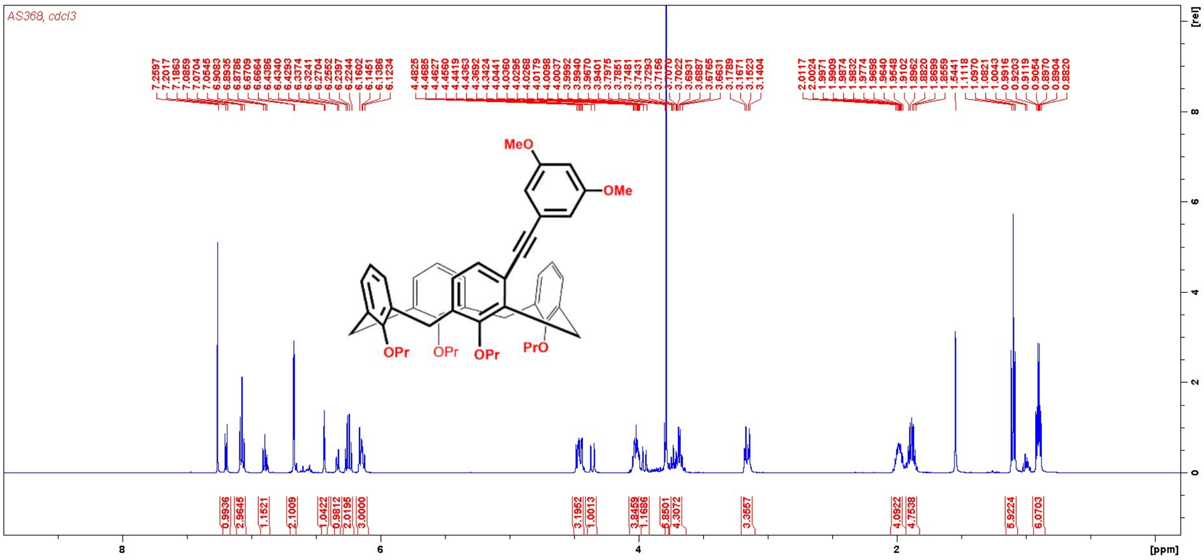

Figure S1: ^{1}H NMR of compound **5a** ($CDCl_3$, 500 MHz, 298 K)

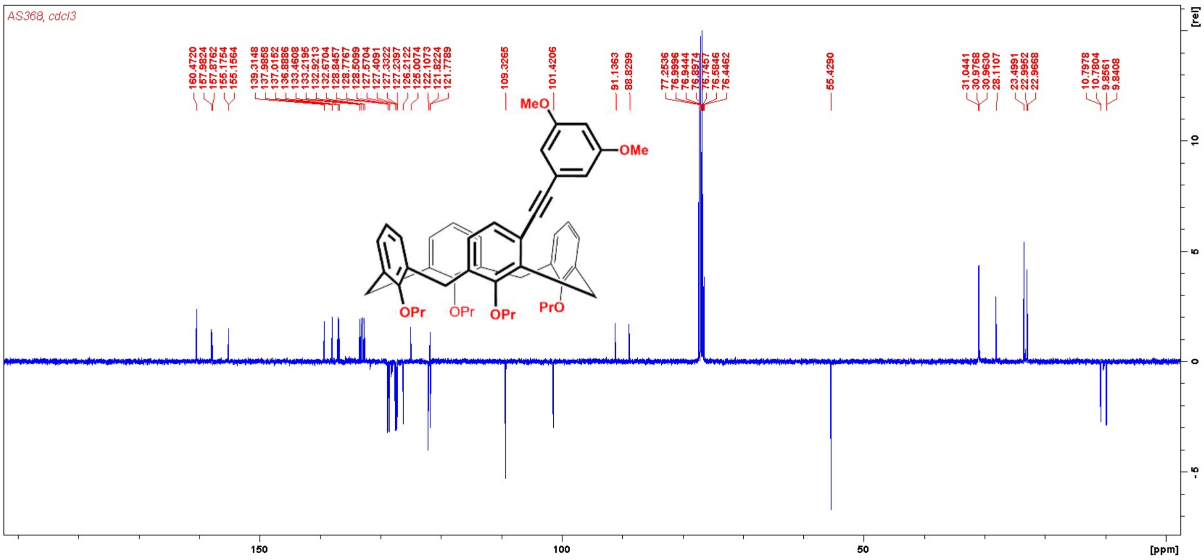

Figure S2: ^{13}C(APT) NMR of compound **5a** ($CDCl_3$, 125 MHz, 298 K)

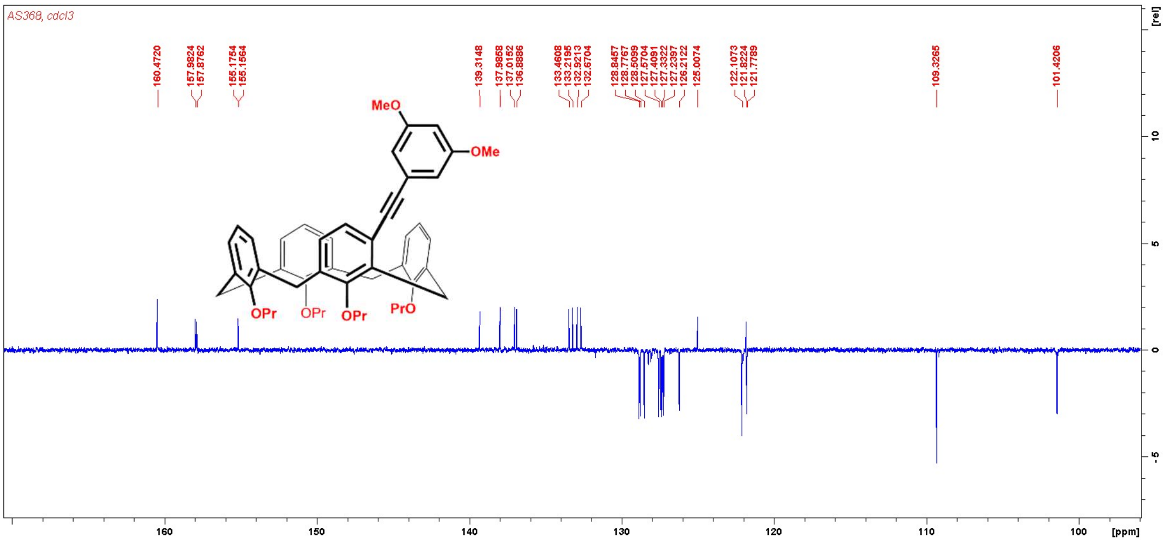

Figure S3: ^{13}C(APT) NMR of compound **5a,** aromatic region (CDCl$_3$, 125 MHz, 298 K)

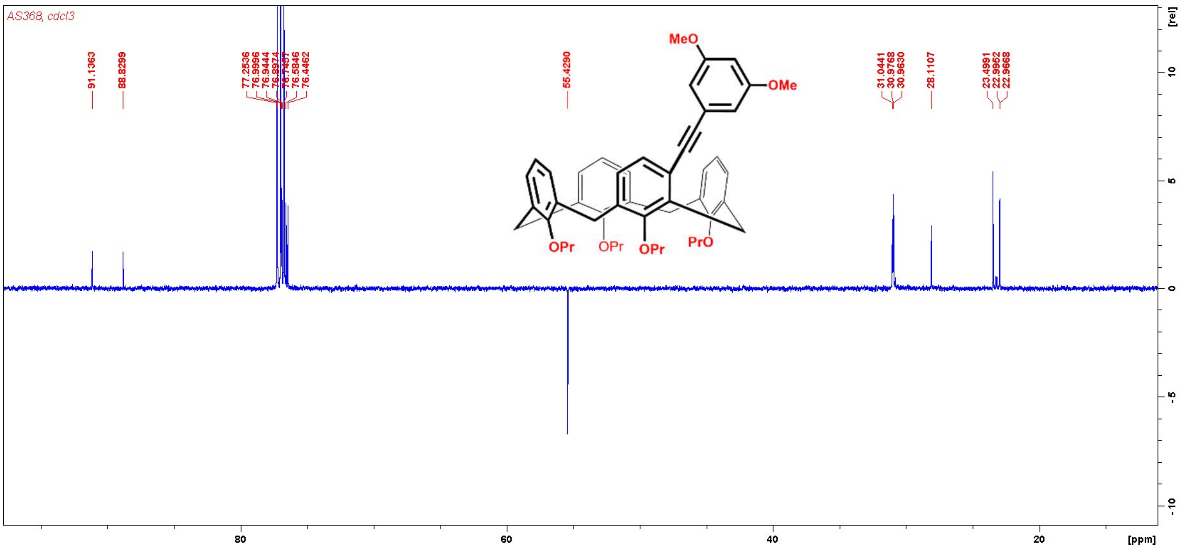

Figure S4: ^{13}C(APT) NMR of compound **5a,** aliphatic region (CDCl$_3$, 125 MHz, 298 K)

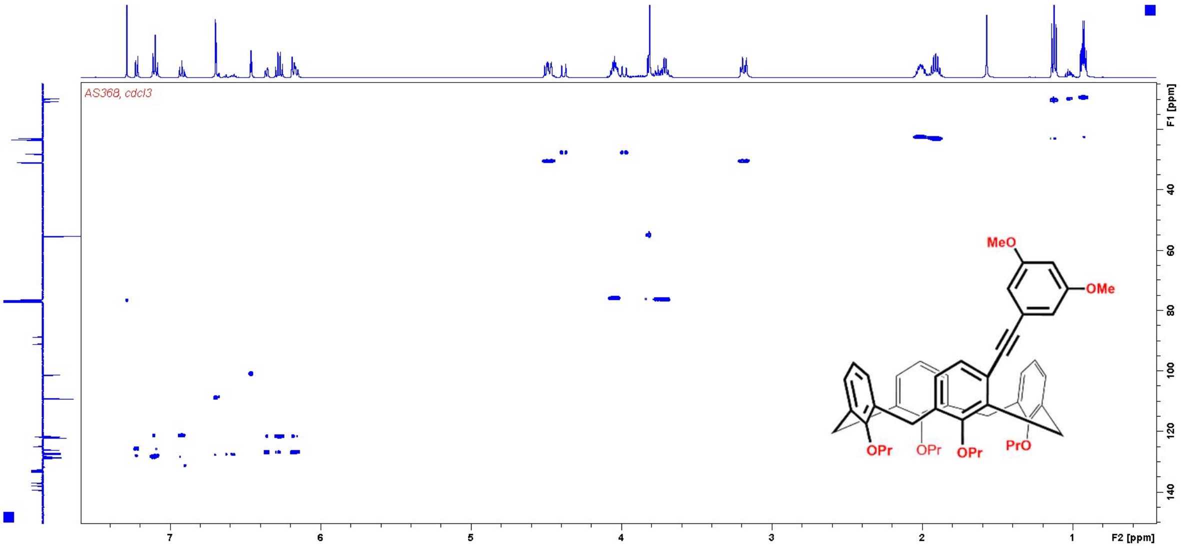

Figure S5: HSQC NMR of compound **5a** (CDCl$_3$, 125 MHz, 298 K)

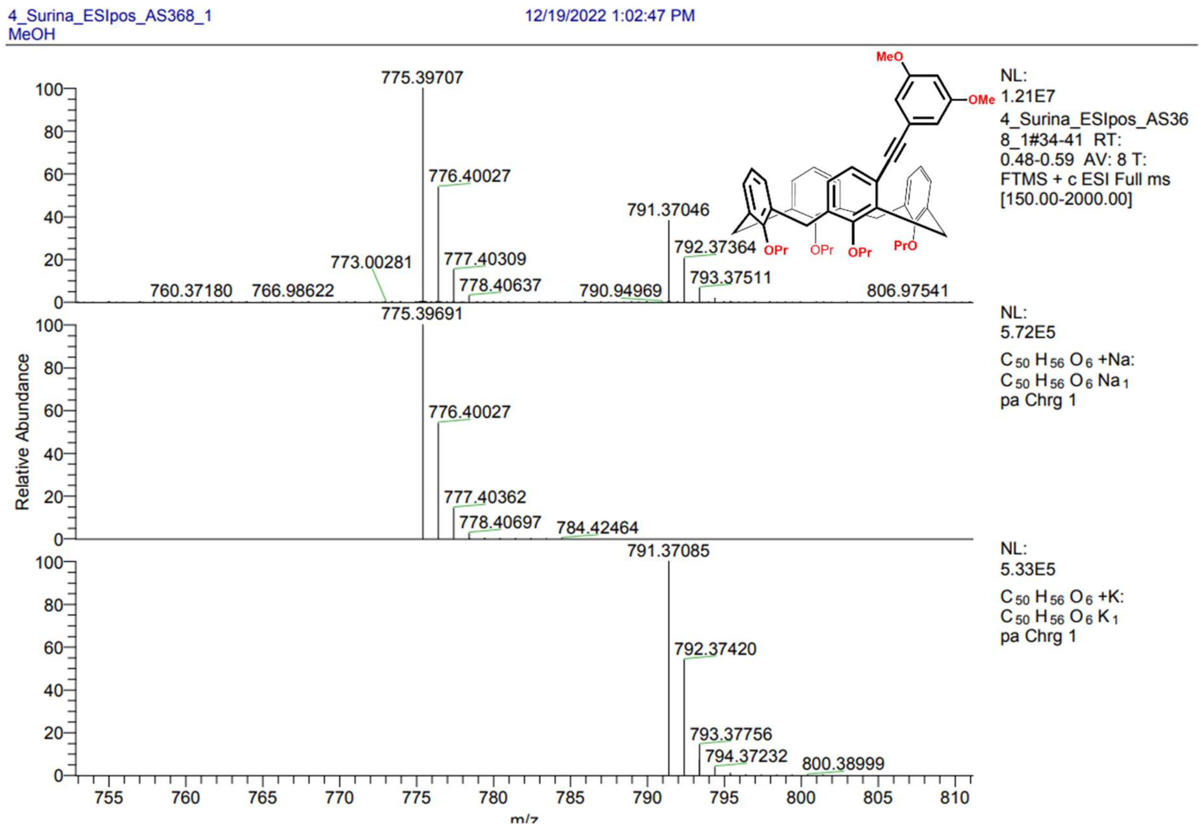

Figure S6: HRMS of compound **5a** (ESI$^+$)

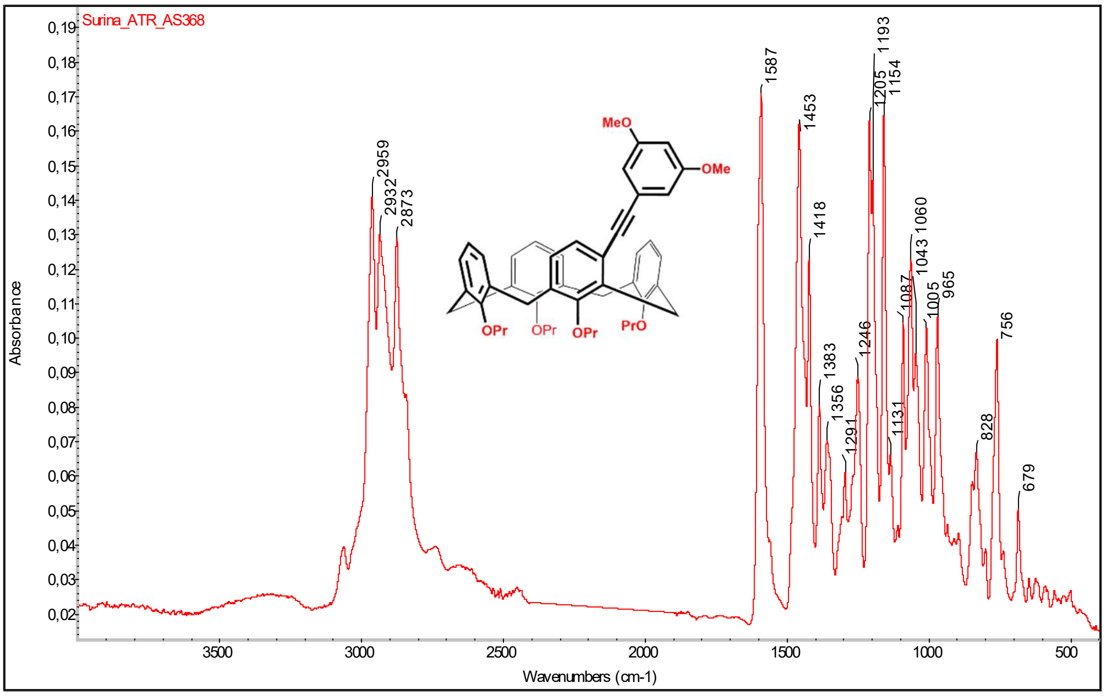

Figure S7: IR of compound **5a** (KBr)

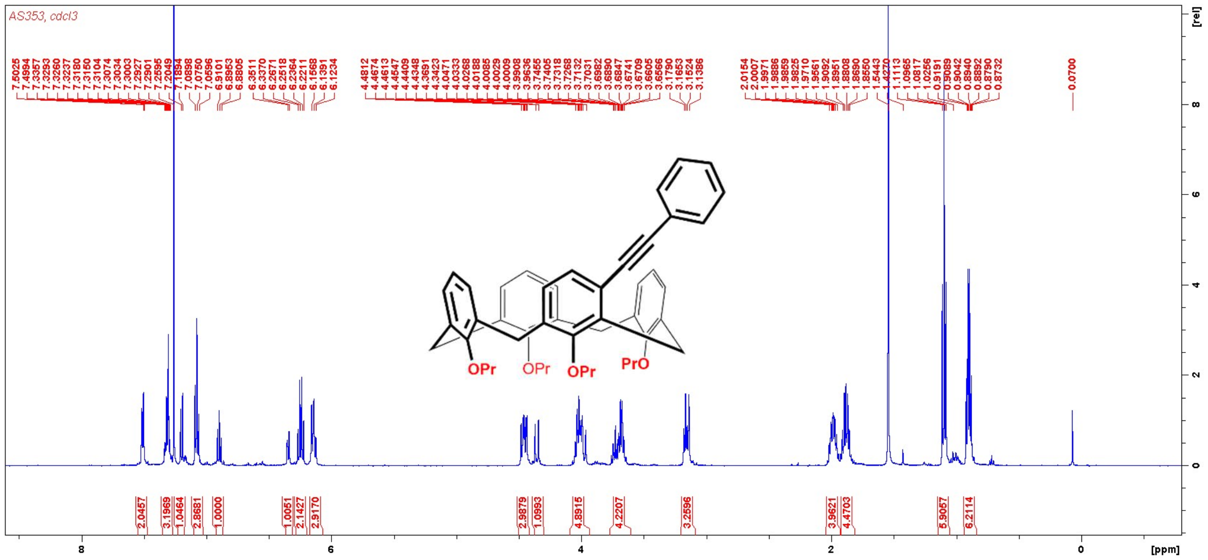

Figure S8: ^{1}H NMR of compound **5b** ($CDCl_3$, 500 MHz, 298 K)

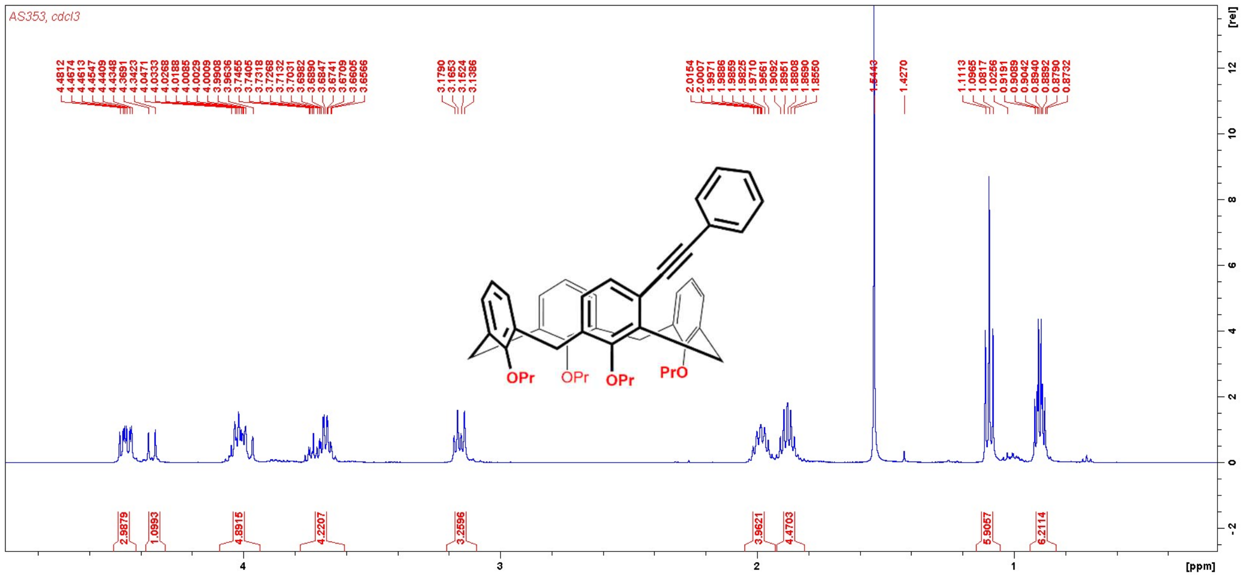

Figure S9: ^{1}H NMR of compound **5b,** aliphatic region ($CDCl_3$, 500 MHz, 298 K)

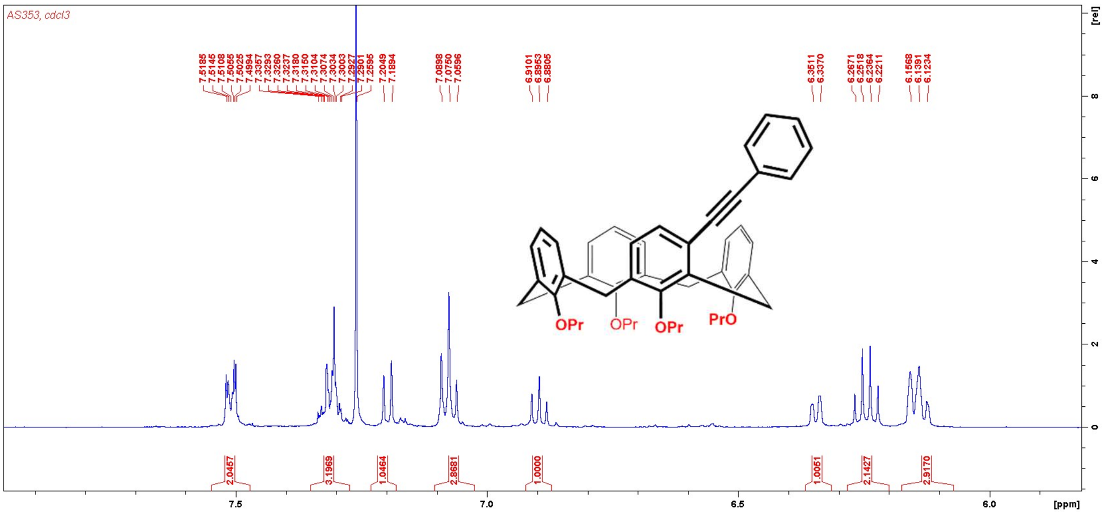

Figure S10: ^{1}H NMR of compound **5b,** aromatic region ($CDCl_3$, 500 MHz, 298 K)

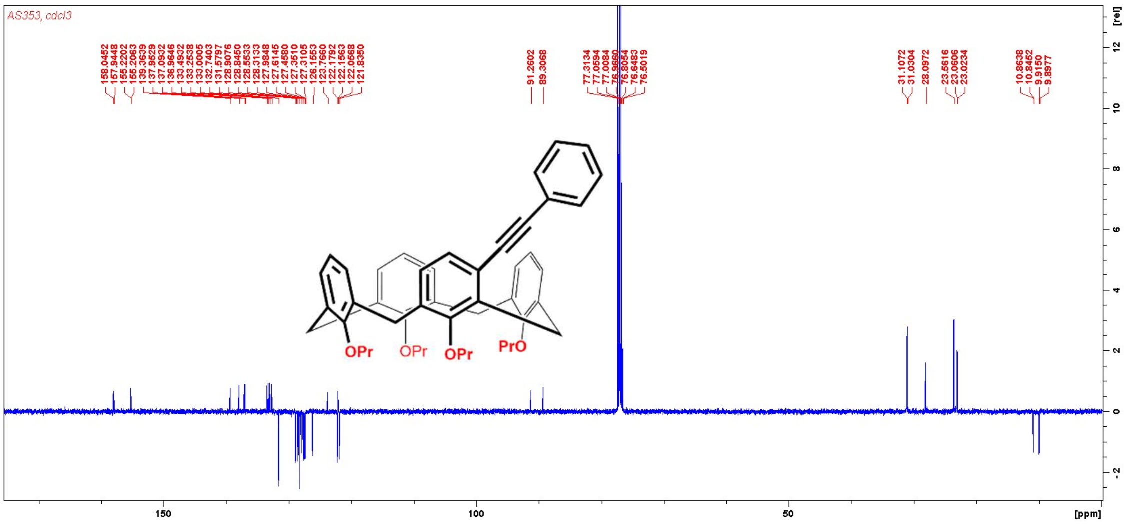

Figure S11: ^{13}C(APT) NMR of compound **5b** ($CDCl_3$, 125 MHz, 298 K)

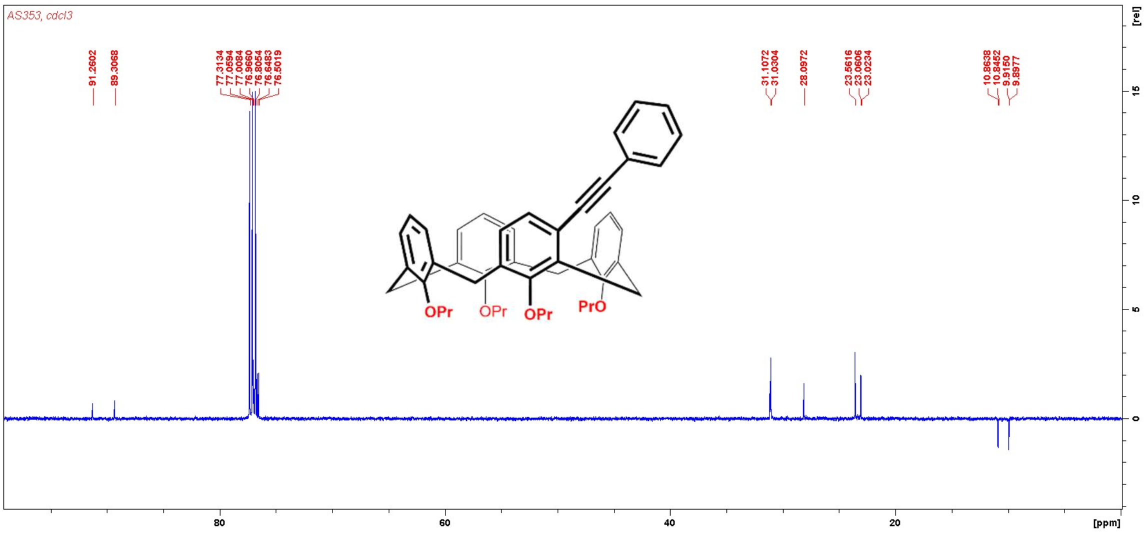

Figure S12: ^{13}C(APT) NMR of compound **5b,** aliphatic region ($CDCl_3$, 125 MHz, 298 K)

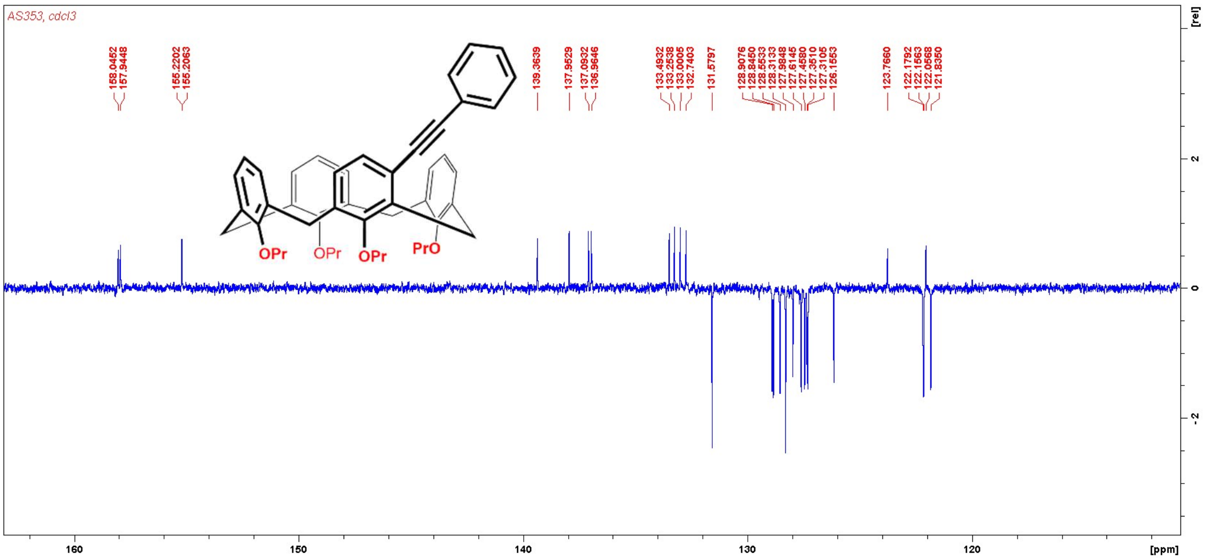

Figure S13: ^{13}C(APT) NMR of compound **5b,** aromatic region ($CDCl_3$, 125 MHz, 298 K)

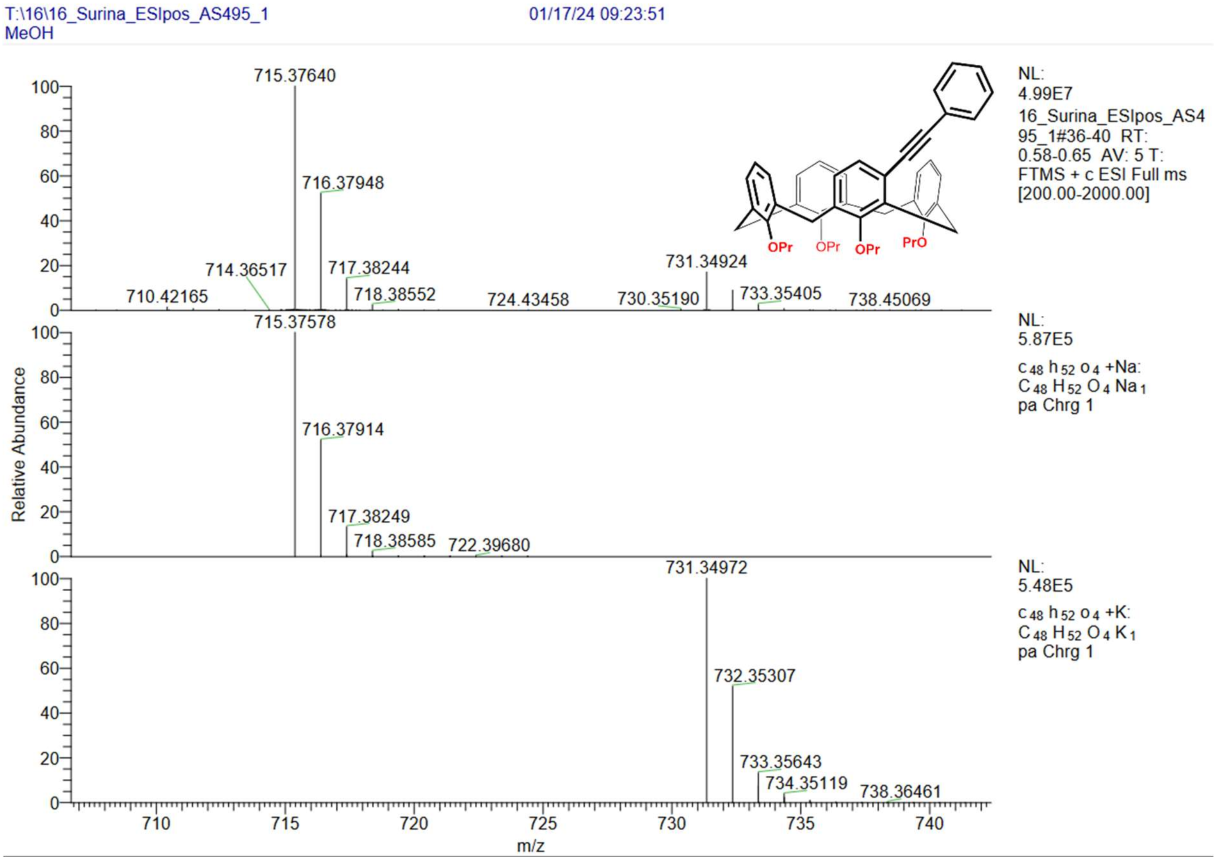

Figure 14: HRMS of compound **5b** (ESI^+)

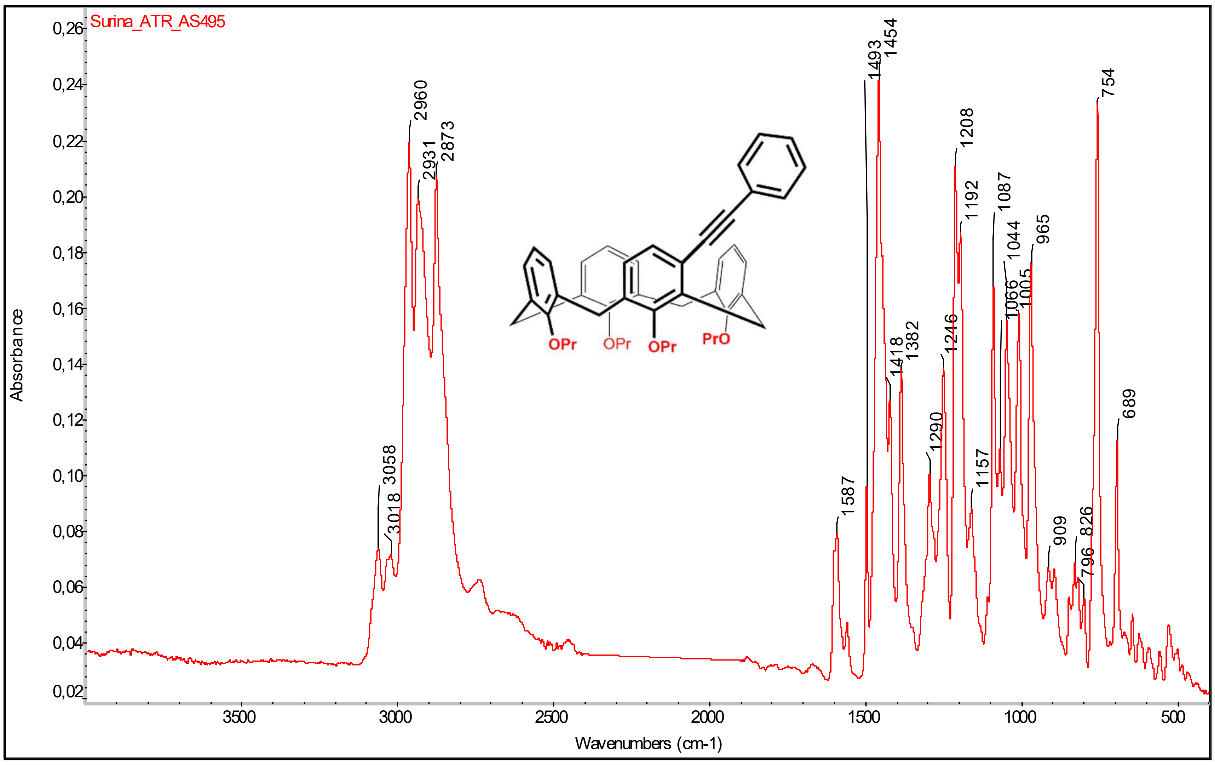

Figure S15: IR of compound **5b** (KBr)

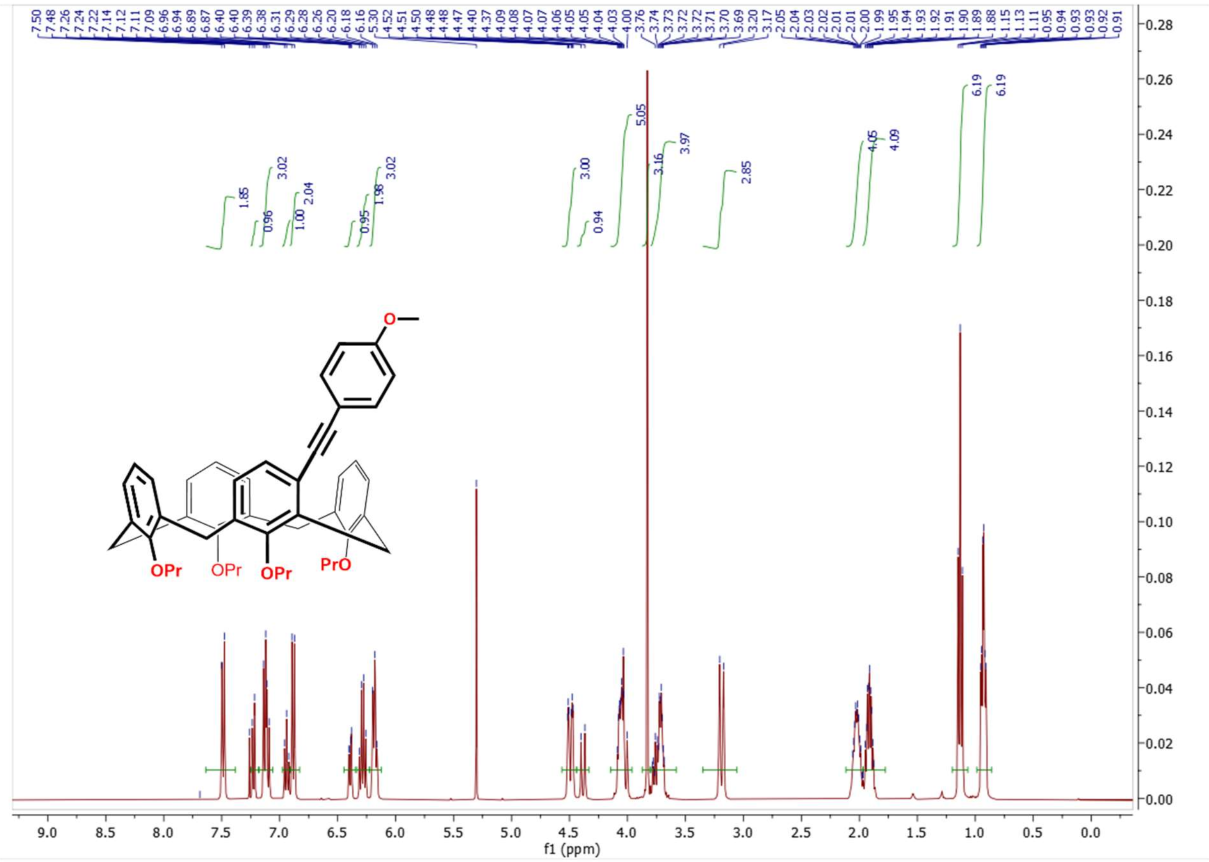

Figure S16: ^{1}H NMR of compound **5c** ($CDCl_3$, 400 MHz, 298 K)

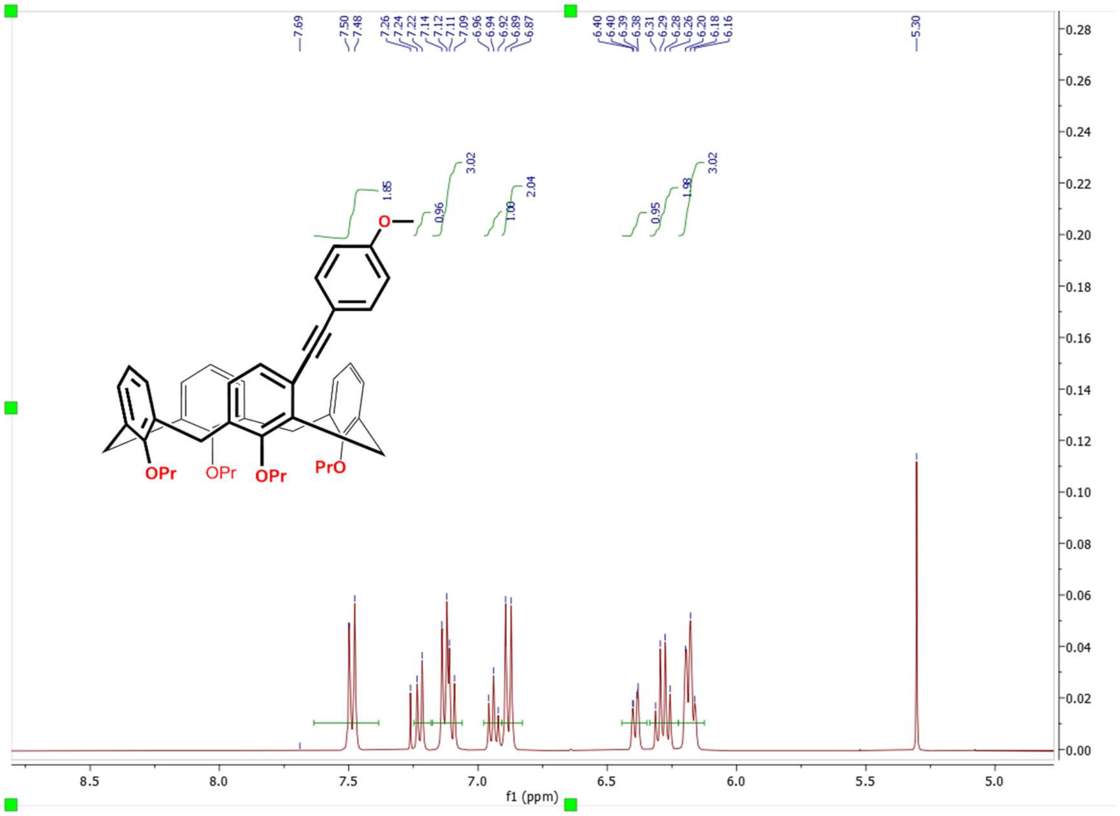

Figure S17: ^{1}H NMR of compound **5c**, aromatic region ($CDCl_3$, 400 MHz, 298 K)

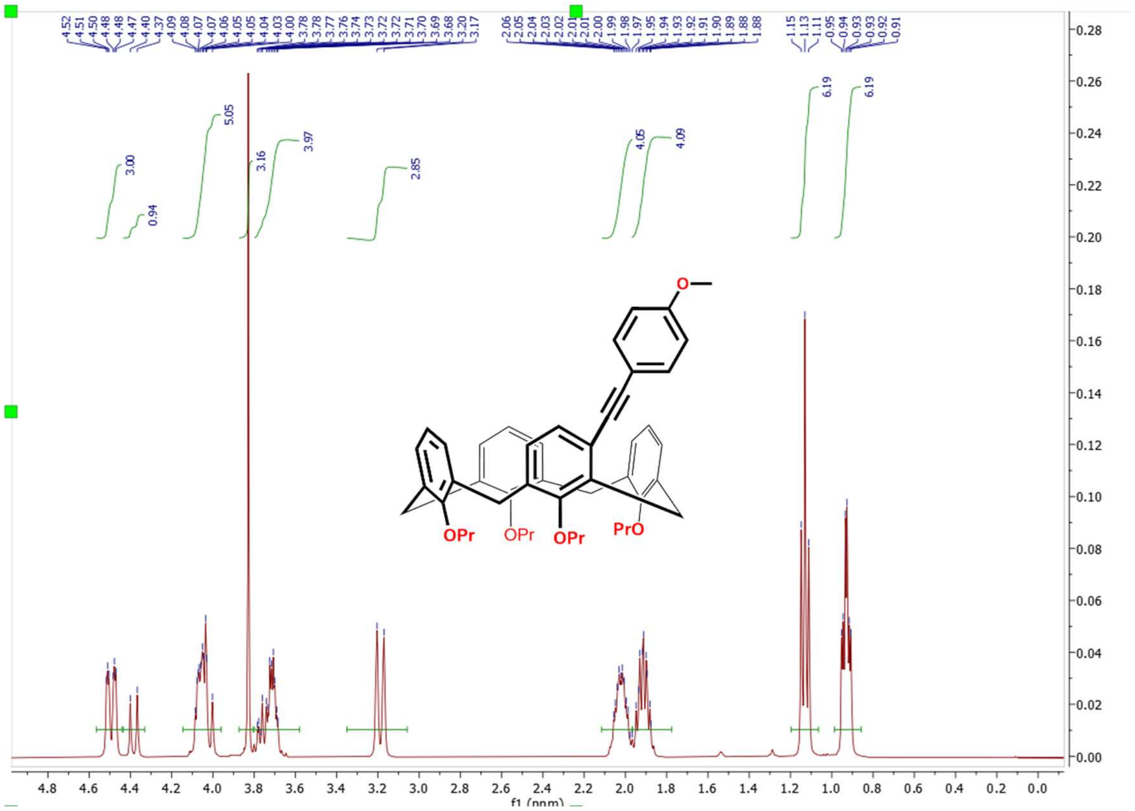

Figure S18: ^{1}H NMR of compound **5c**, aliphatic region ($CDCl_3$, 400 MHz, 298 K)

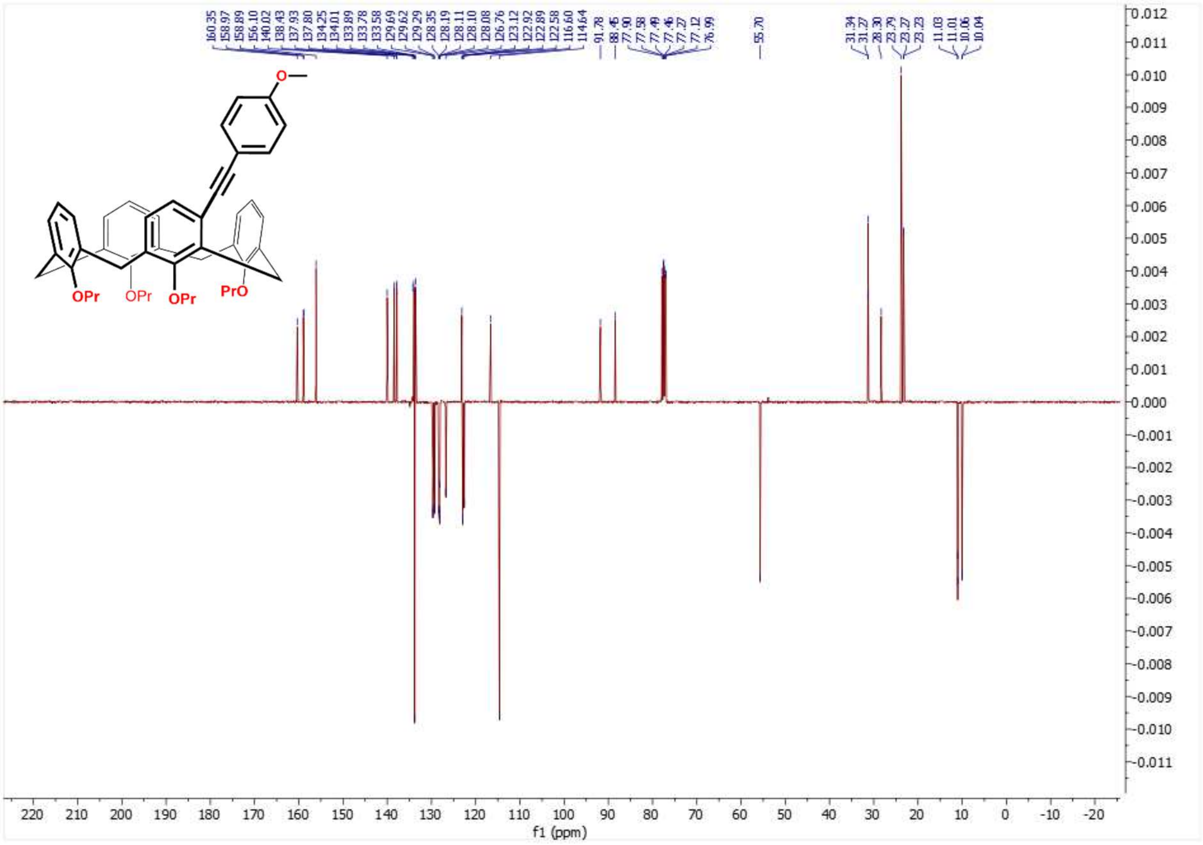

Figure S19: ^{13}C(APT) NMR of compound **5c** (CDCl$_3$, 100 MHz, 298 K)

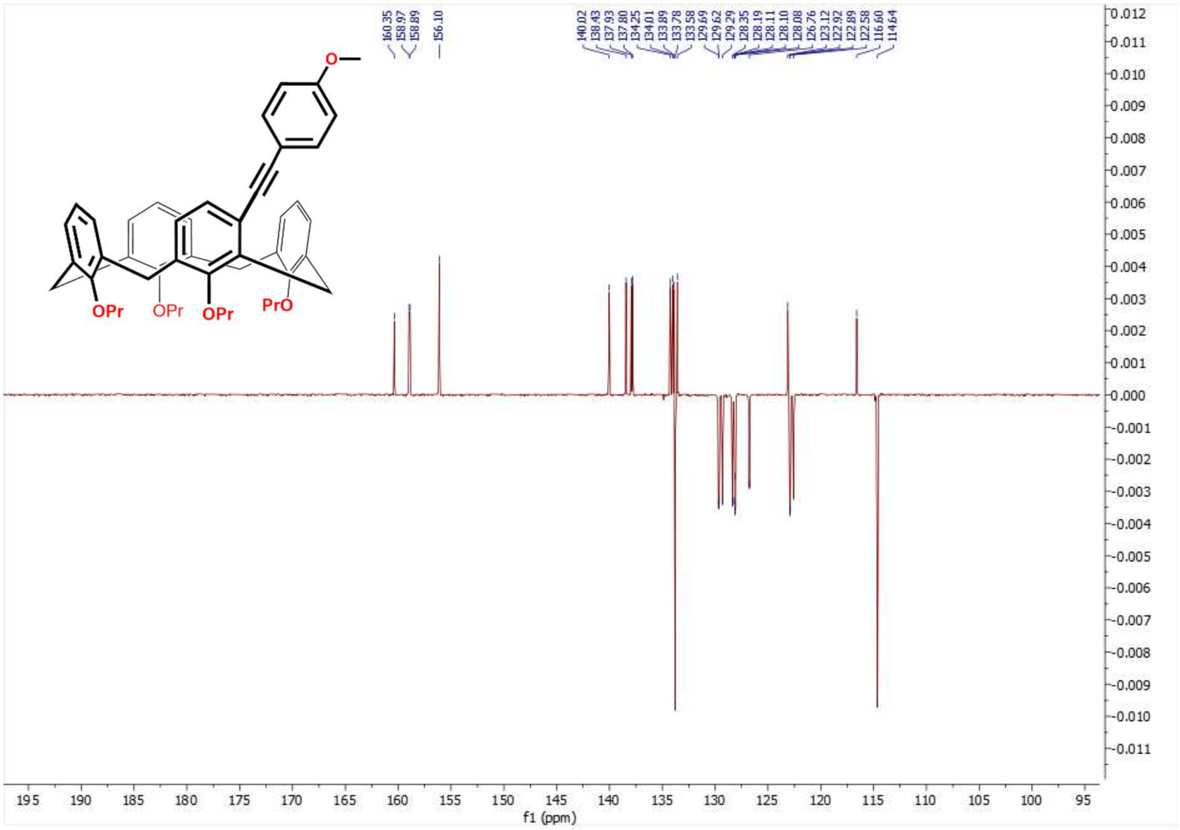

Figure S20: ^{13}C(APT) NMR of compound **5c,** aromatic region (CDCl$_3$, 100 MHz, 298 K)

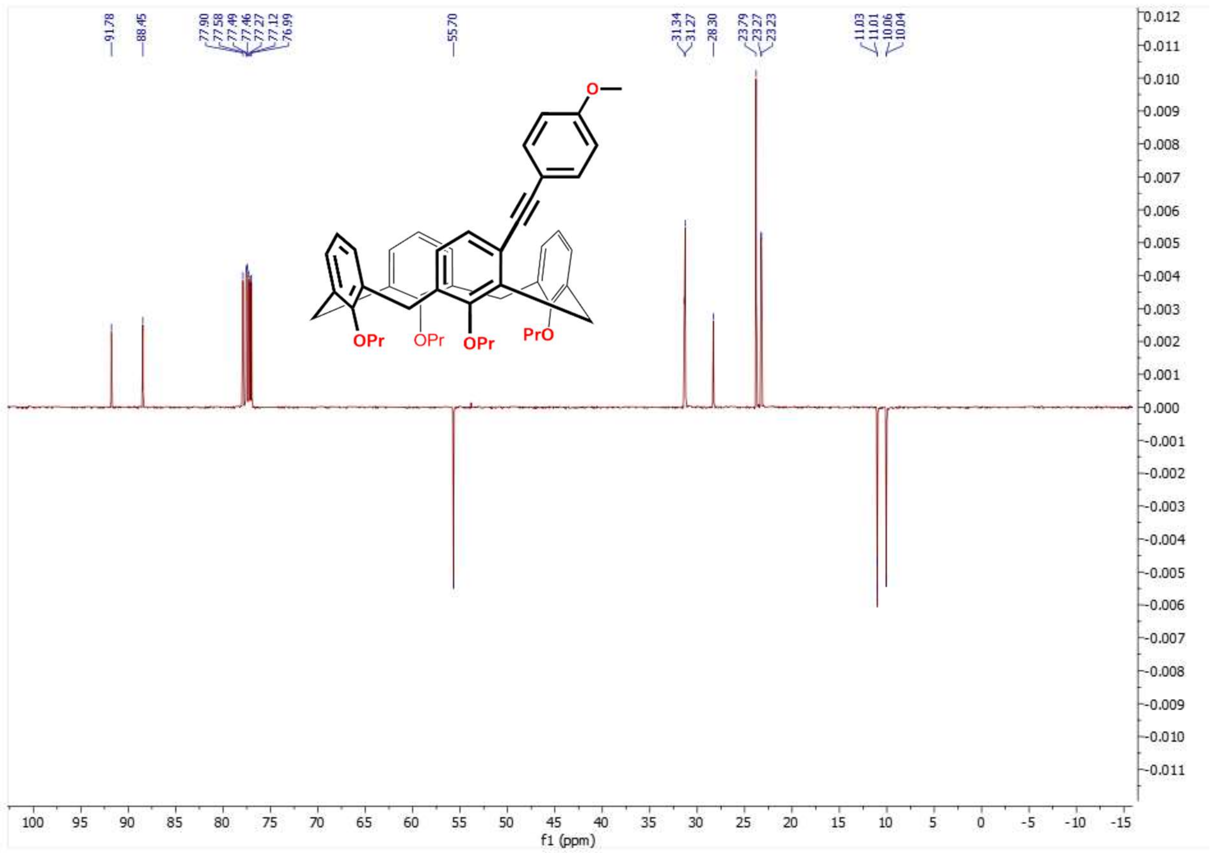

Figure S21: ^{13}C(APT) NMR of compound **5c,** aliphatic region ($CDCl_3$, 100 MHz, 298 K)

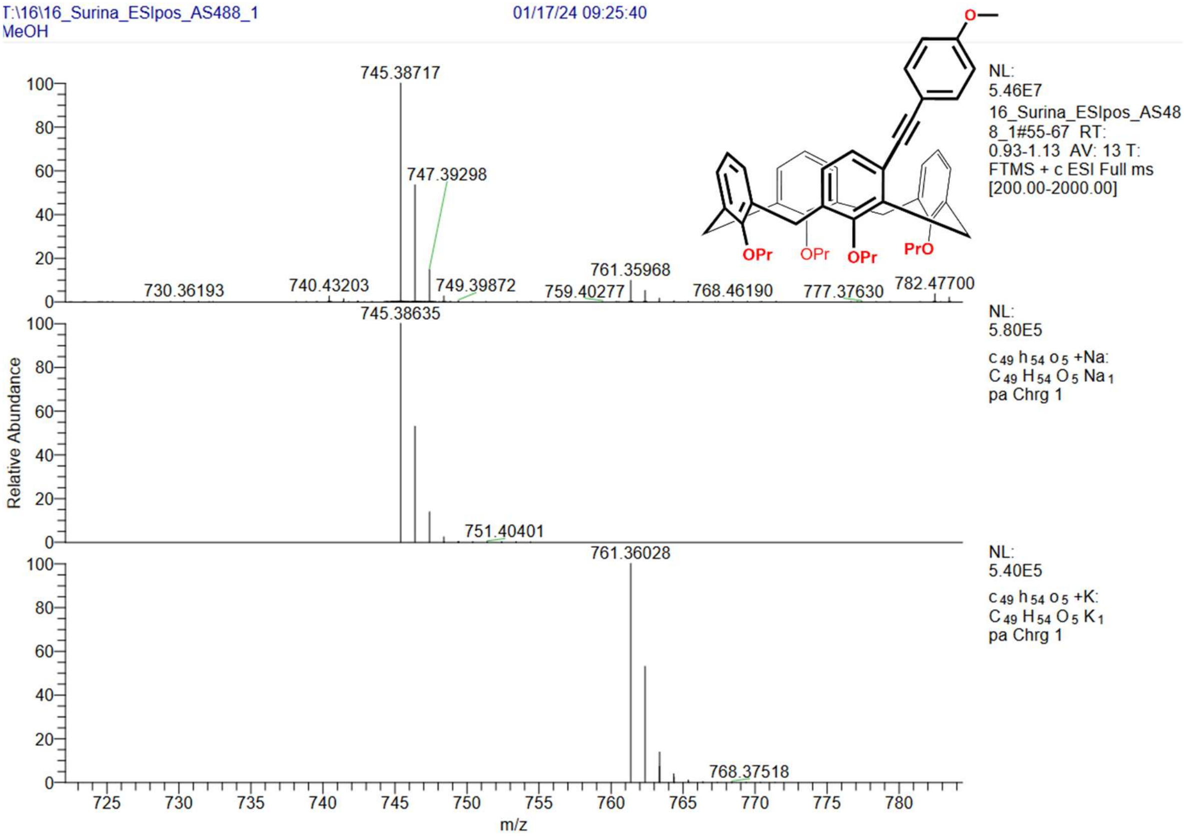

Figure S22: HRMS of compound **5c** (ESI+)

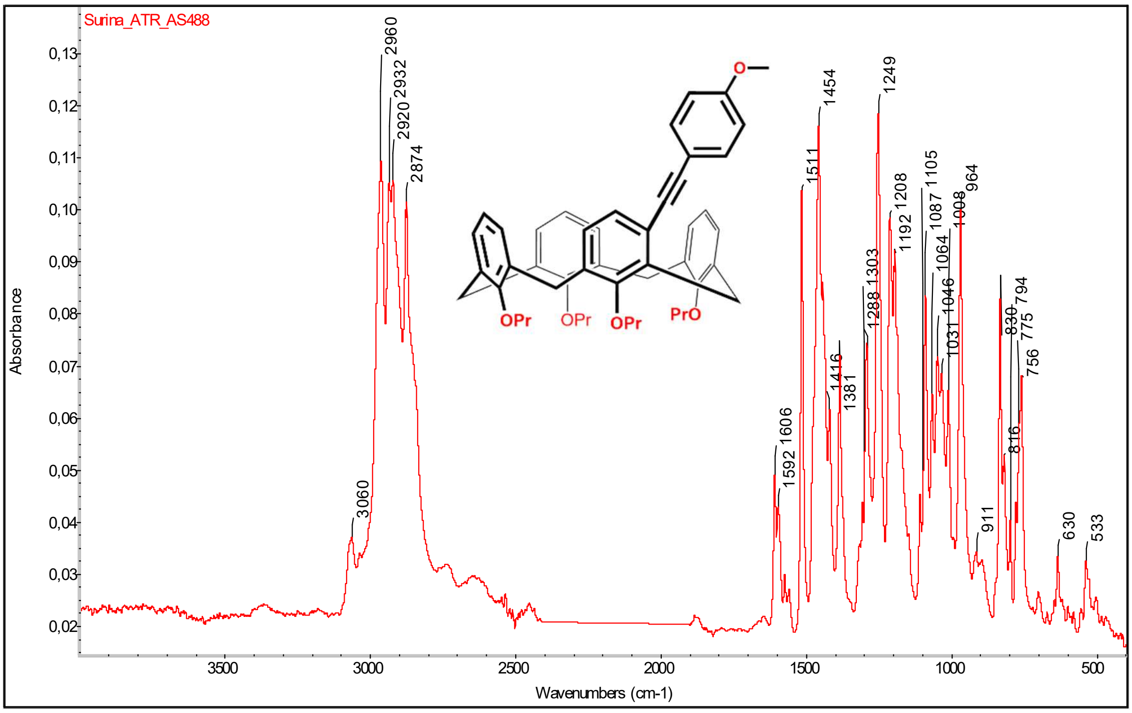

Figure S23: IR of compound **5c** (KBr)

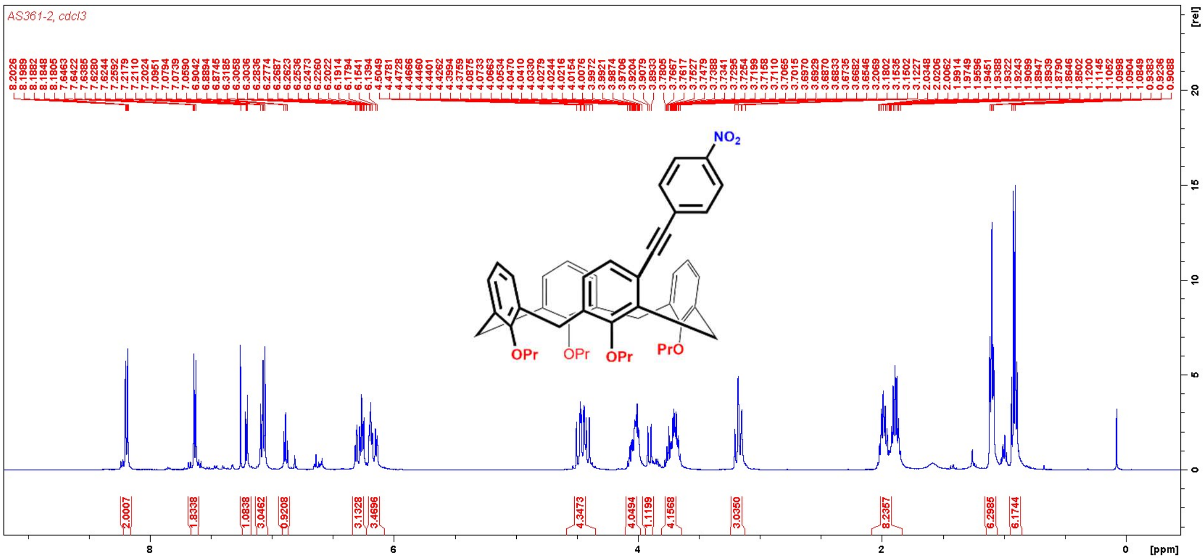

Figure S24: ^{1}H NMR of compound **5d** ($CDCl_3$, 500 MHz, 298 K)

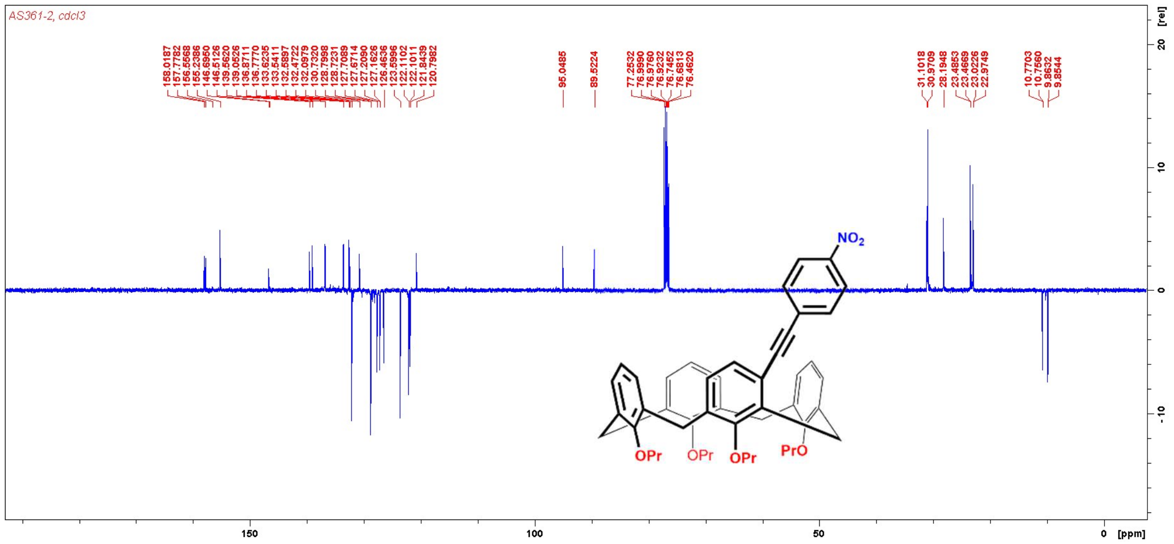

Figure S25: ^{13}C(APT) NMR of compound **5d** ($CDCl_3$, 125 MHz, 298 K)

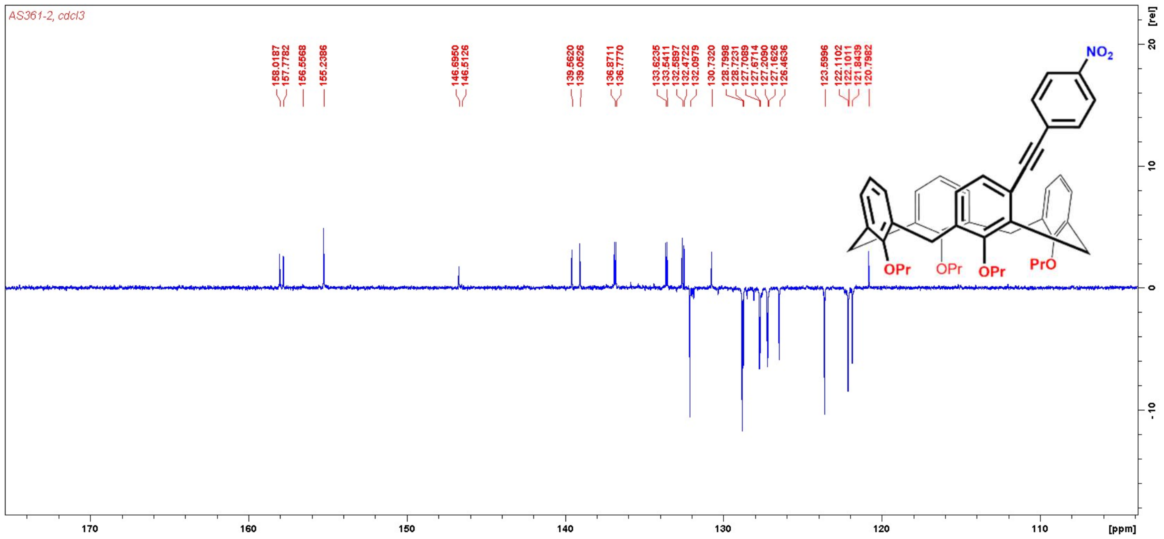

Figure S26: ^{13}C(APT) NMR of compound **5d,** aromatic region ($CDCl_3$, 125 MHz, 298 K)

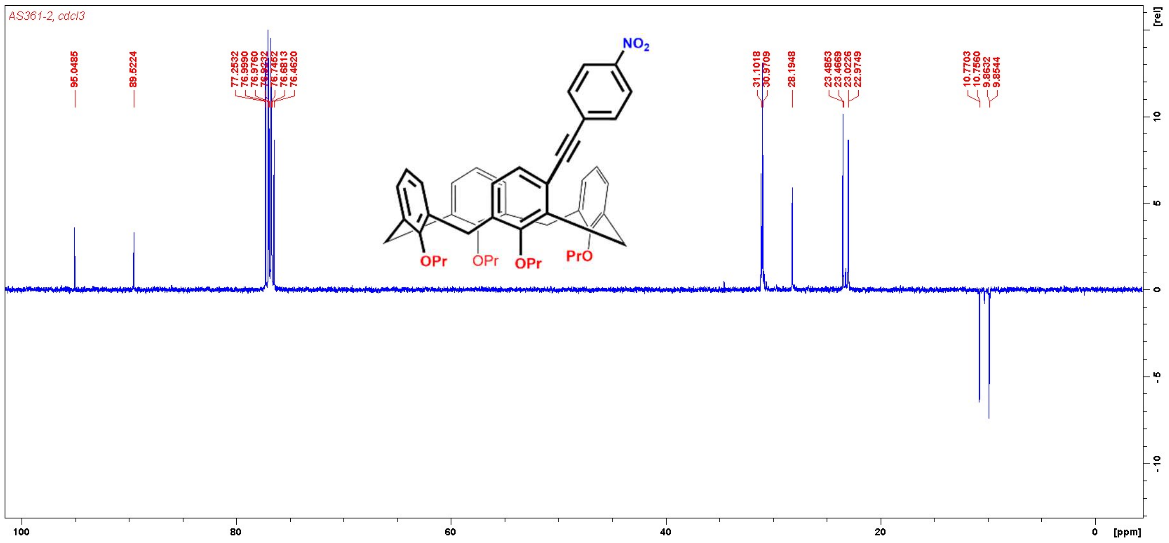

Figure S27: ^{13}C(APT) NMR of compound **5d,** aliphatic region ($CDCl_3$, 125 MHz, 298 K)

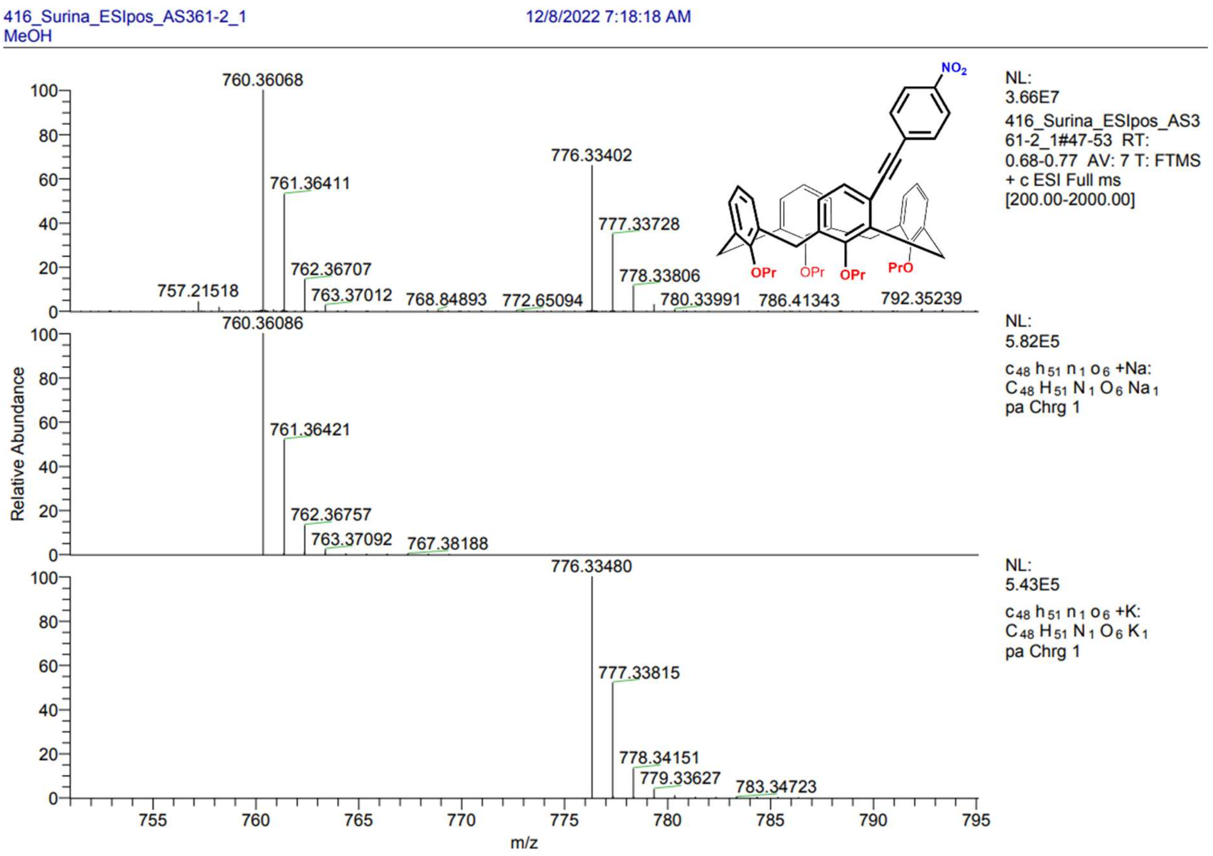

Figure S28: HRMS of compound **5d** (ESI$^+$)

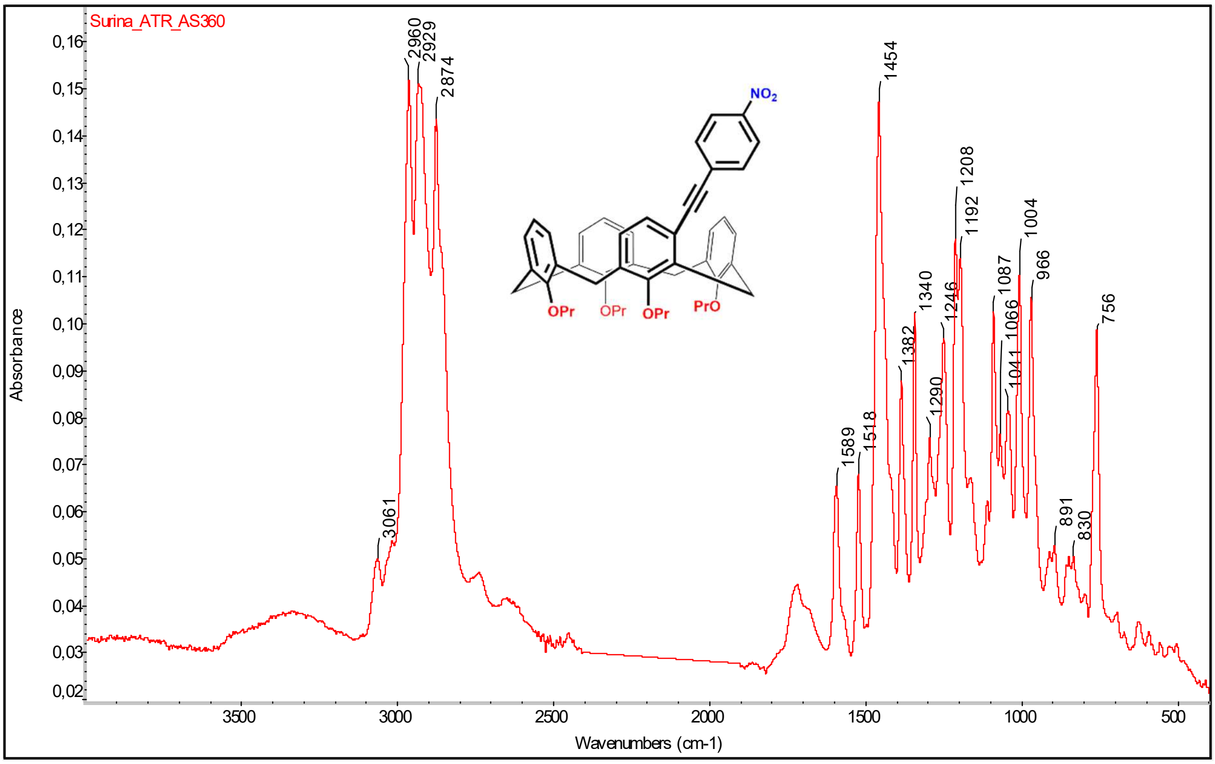

Figure S29: IR of compound **5d** (KBr)

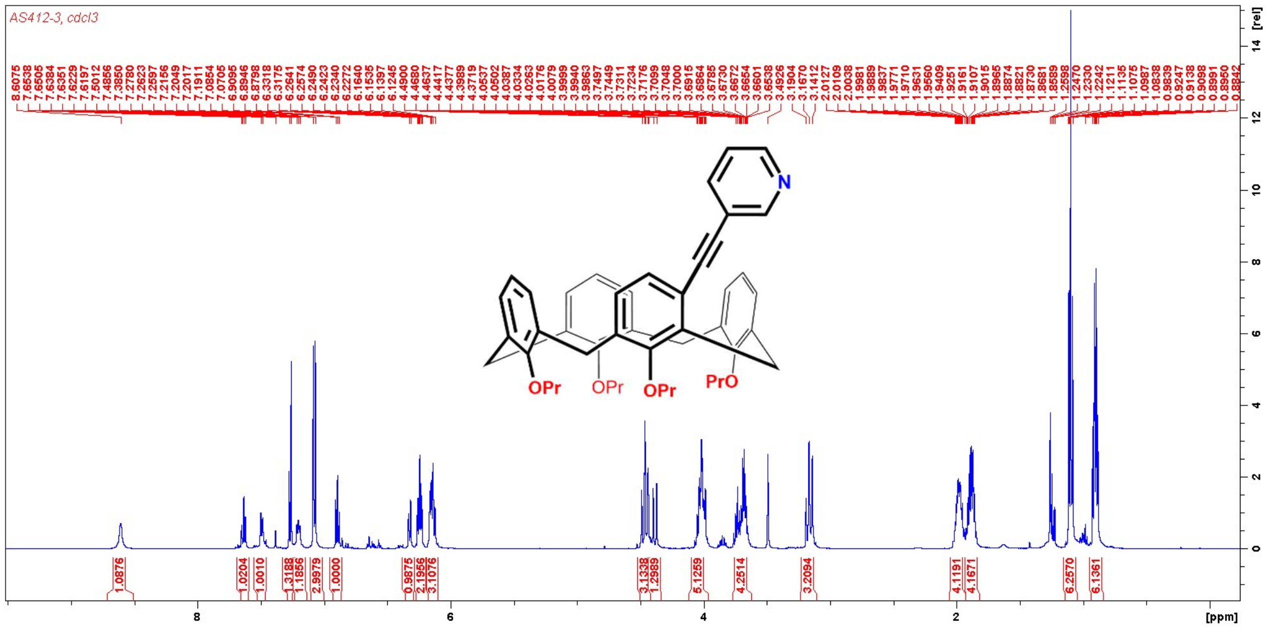

Figure S30: ^{1}H NMR of compound **5e** ($CDCl_3$, 500 MHz, 298 K)

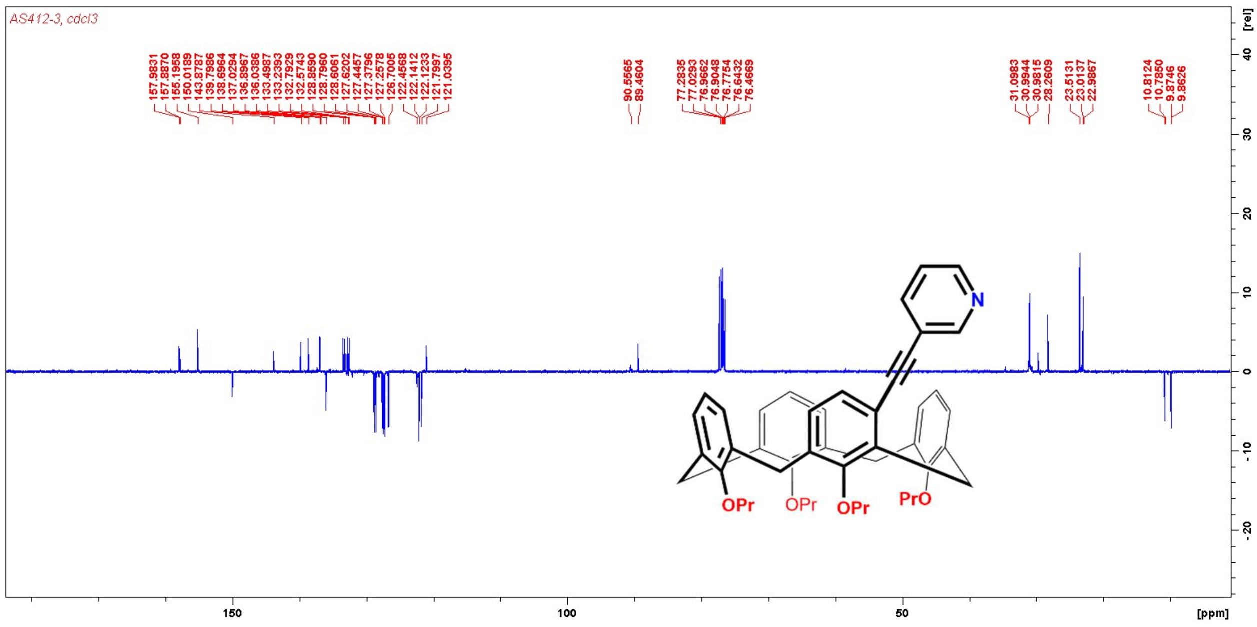

Figure S31: ^{13}C(APT) NMR of compound **5e** ($CDCl_3$, 125 MHz, 298 K)

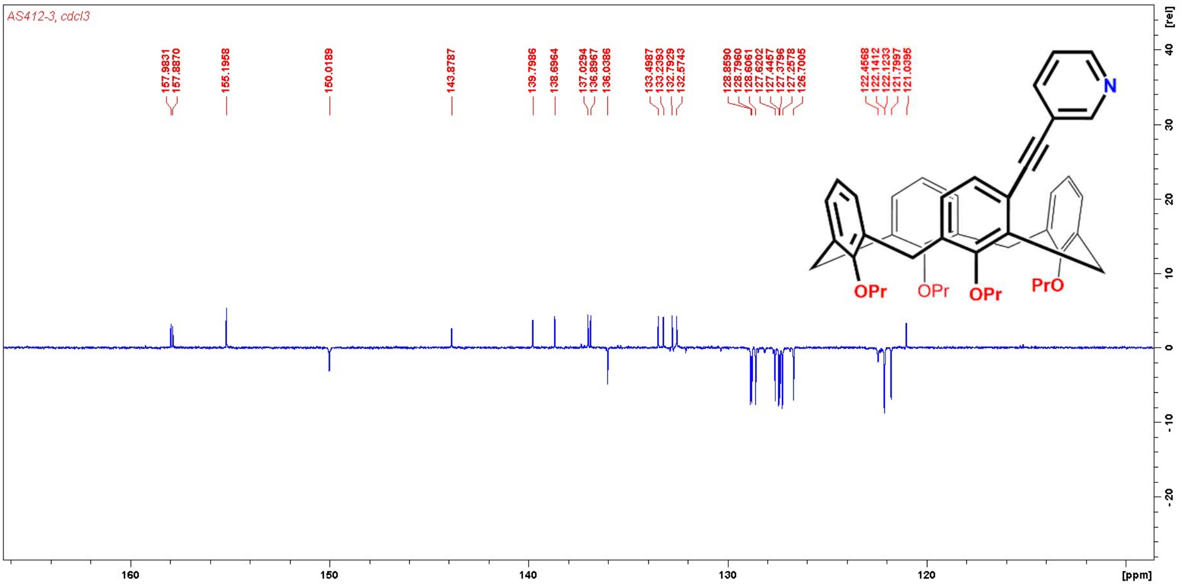

Figure S32: ^{13}C(APT) NMR of compound **5e,** aromatic region ($CDCl_3$, 125 MHz, 298 K)

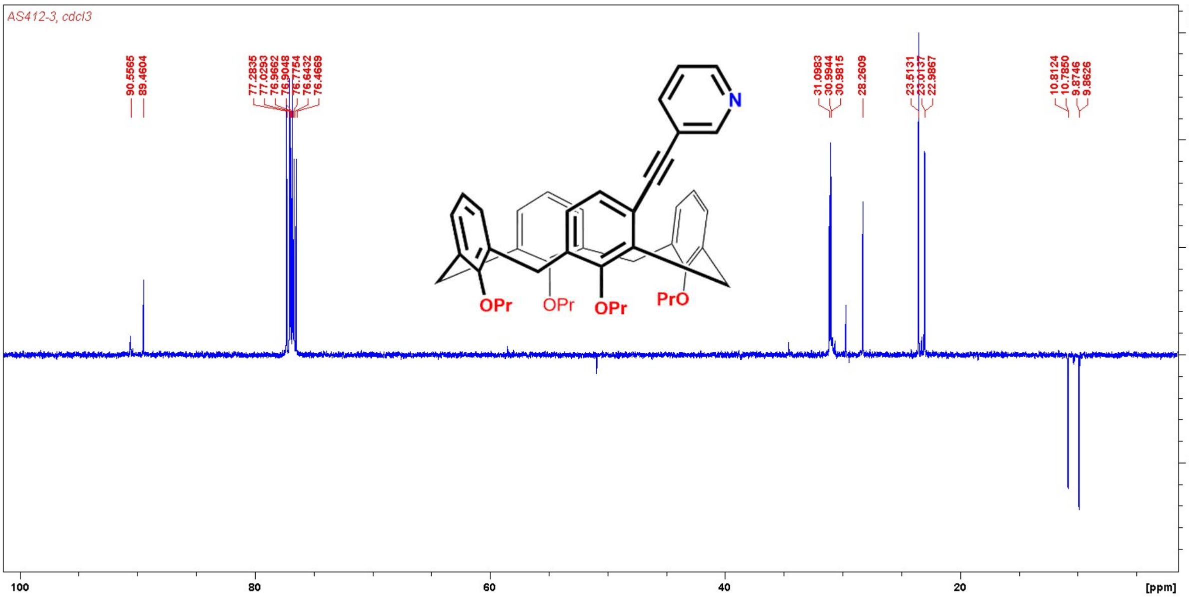

Figure S33: ^{13}C(APT) NMR of compound **5e,** aliphatic region ($CDCl_3$, 125 MHz, 298 K)

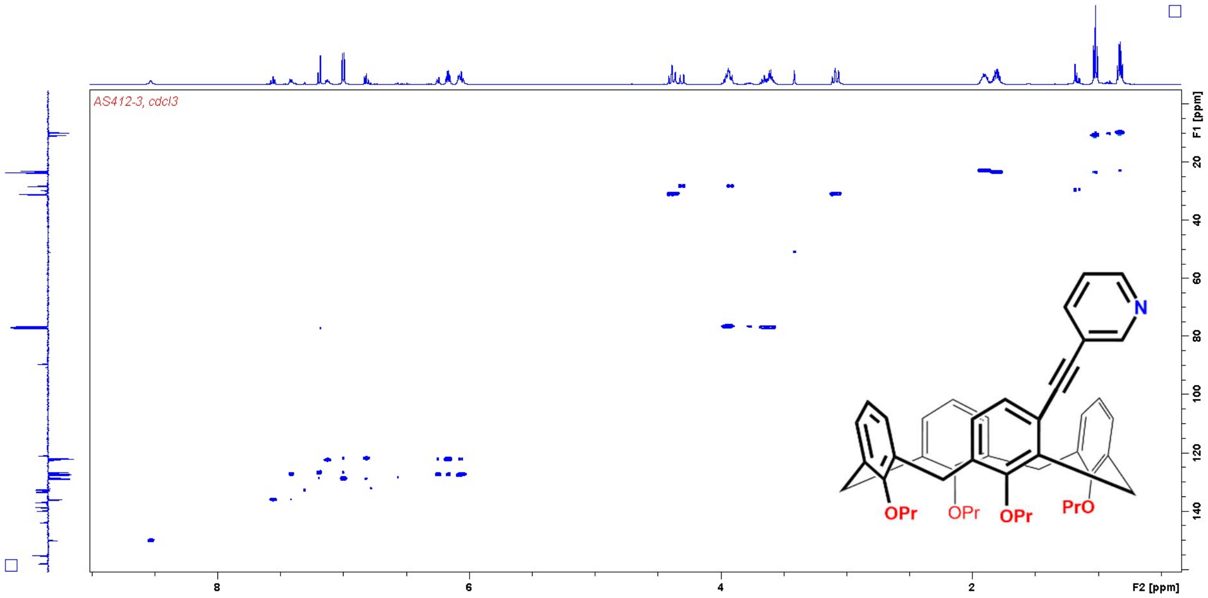

Figure S34: HSQC NMR of compound **5e** ($CDCl_3$, 125 MHz, 298 K)

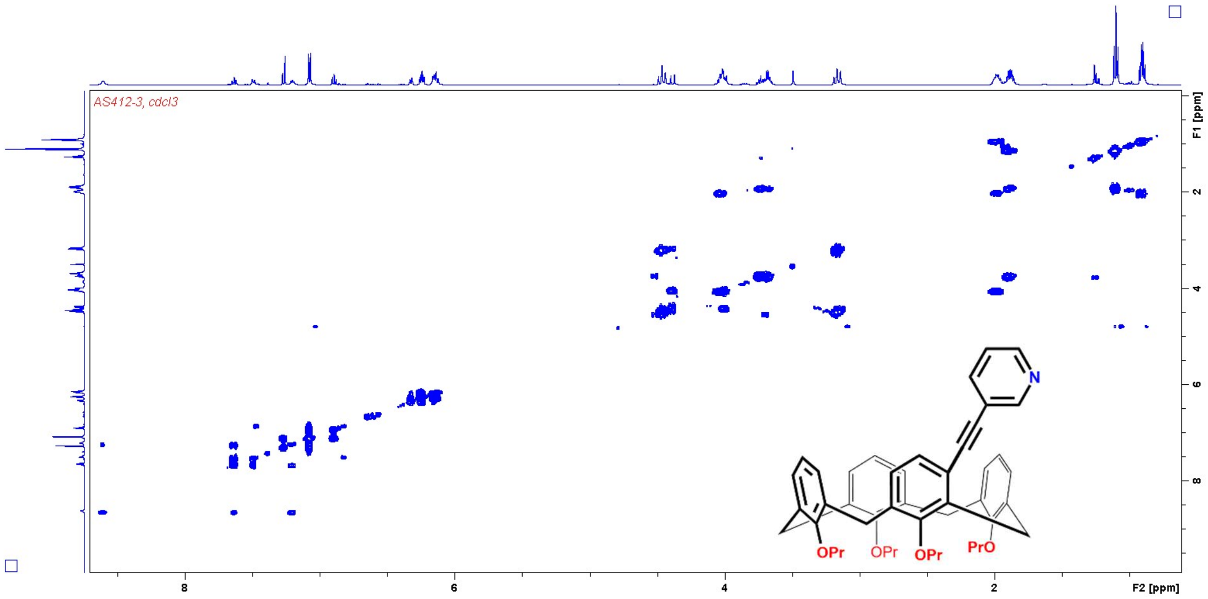

Figure S35: HMBC NMR of compound **5e** ($CDCl_3$, 125 MHz, 298 K)

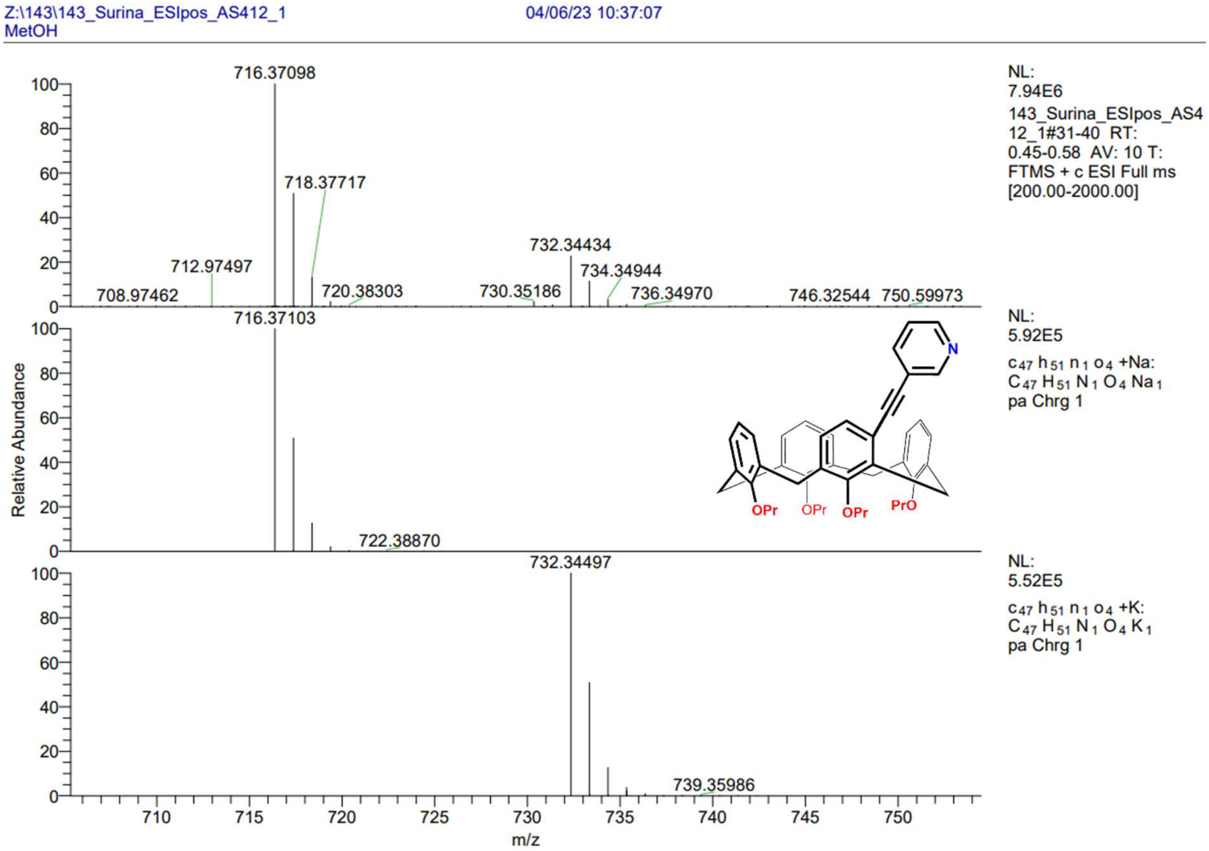

Figure S36: HRMS of compound **5e** (ESI⁺)

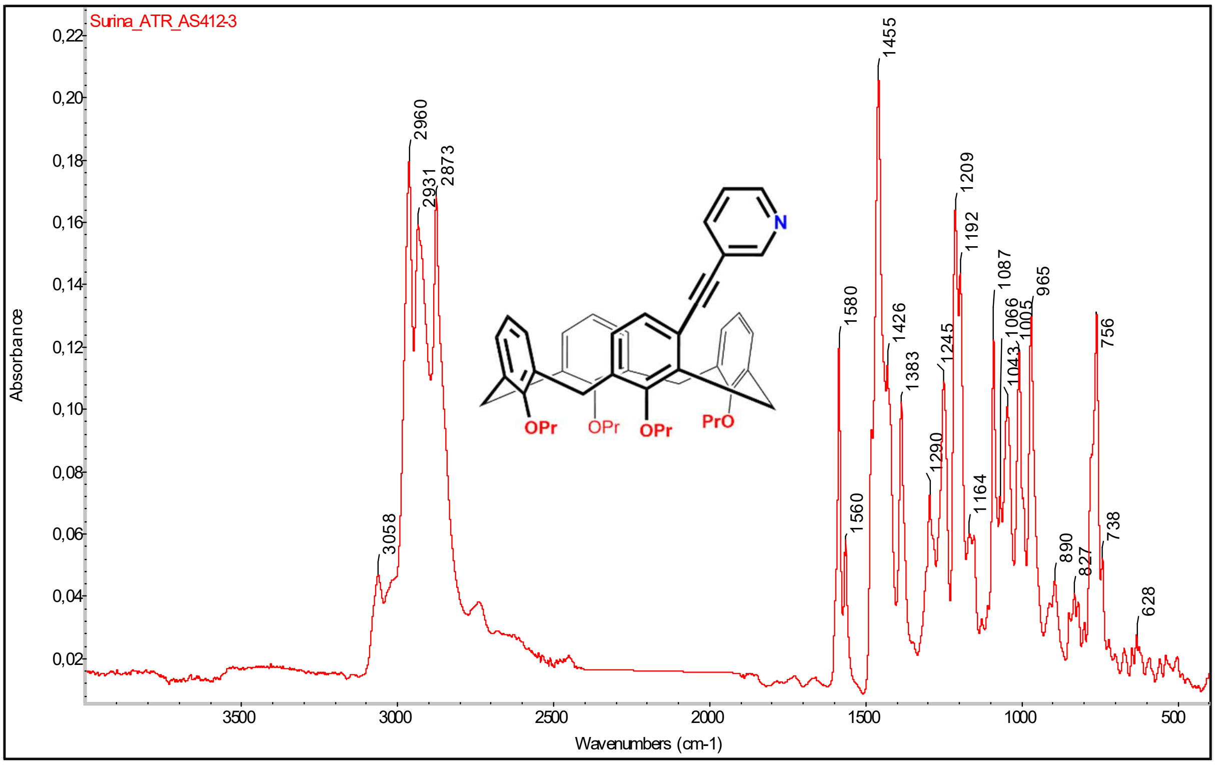

Figure S37: IR of compound **5e** (KBr)

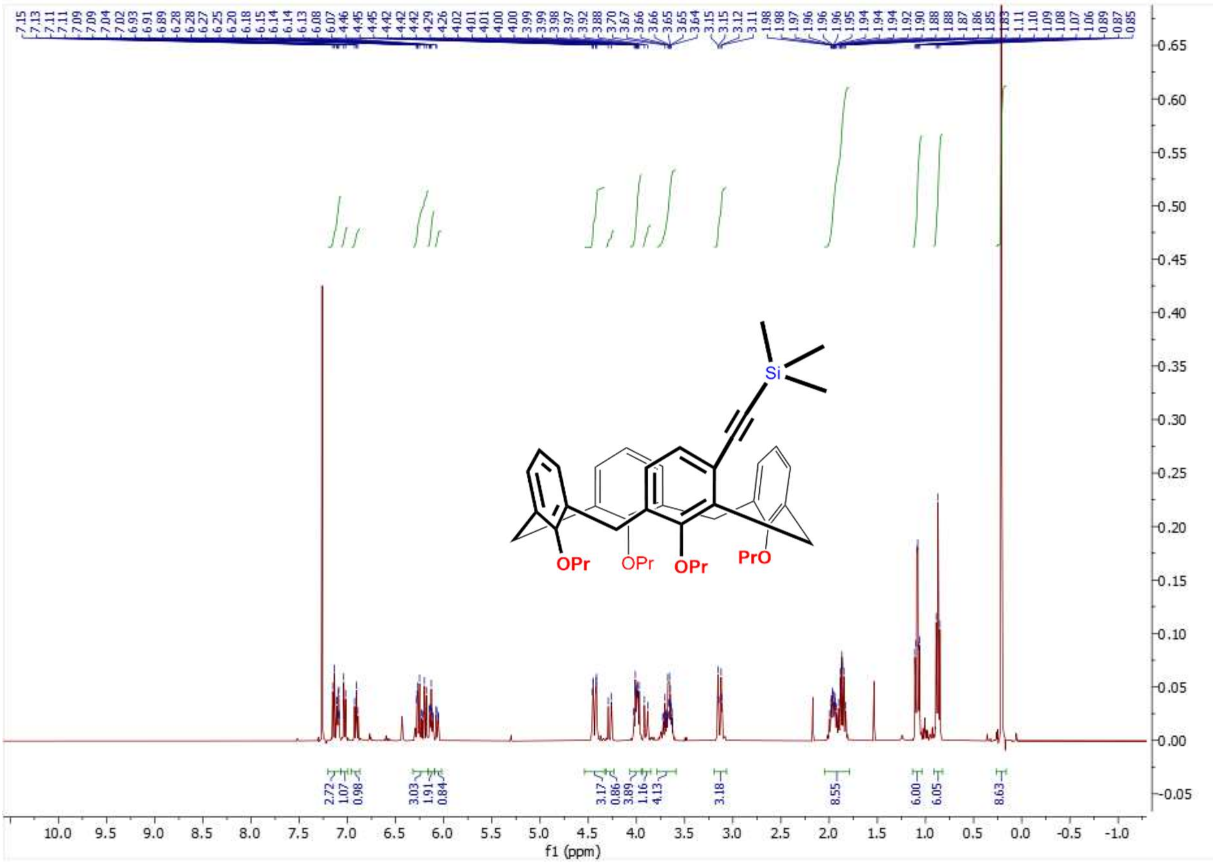

Figure S38: ^{1}H NMR of compound **5f** ($CDCl_3$, 500 MHz, 298 K)

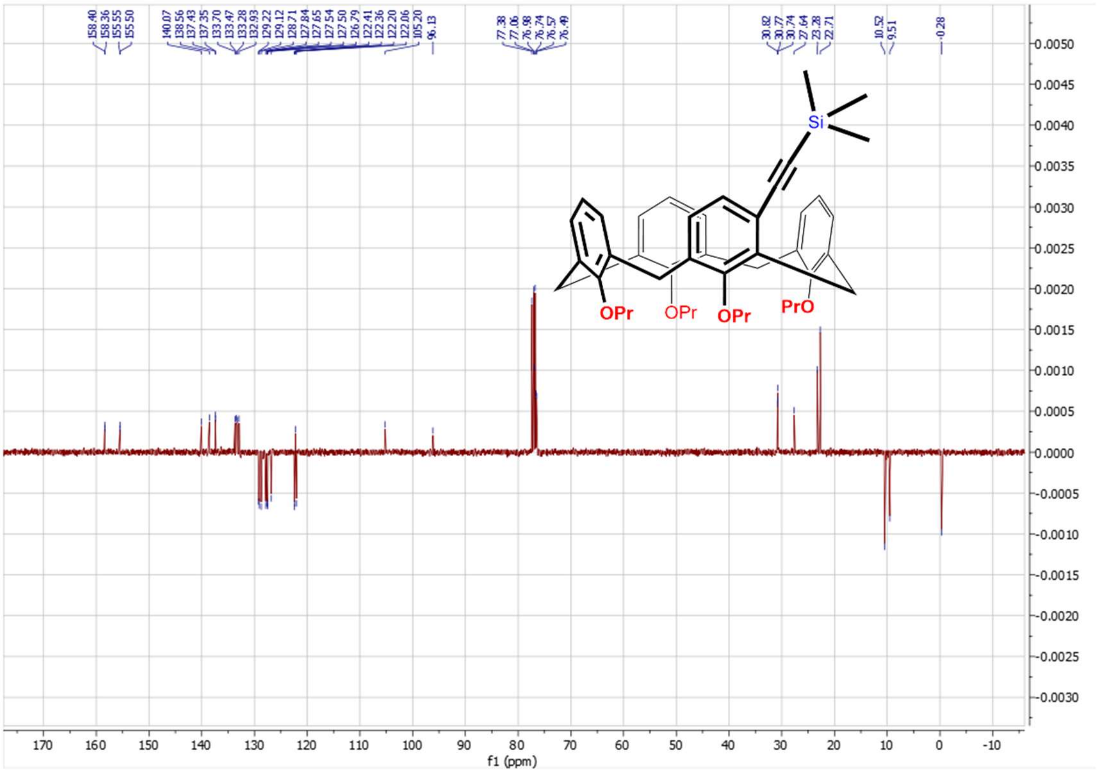

Figure S39: ^{13}C(APT) NMR of compound **5f** ($CDCl_3$, 100 MHz, 298 K)

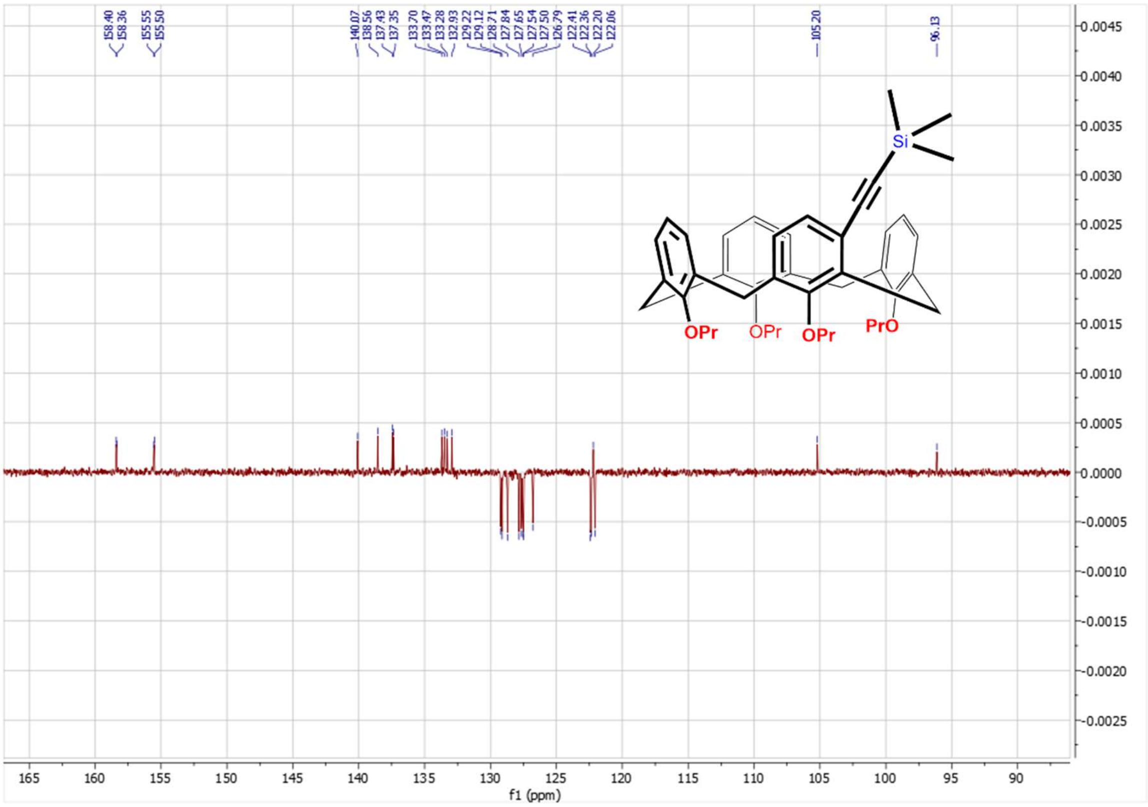

Figure S40: ^{13}C(APT) NMR of compound **5f,** aromatic region ($CDCl_3$, 100 MHz, 298 K)

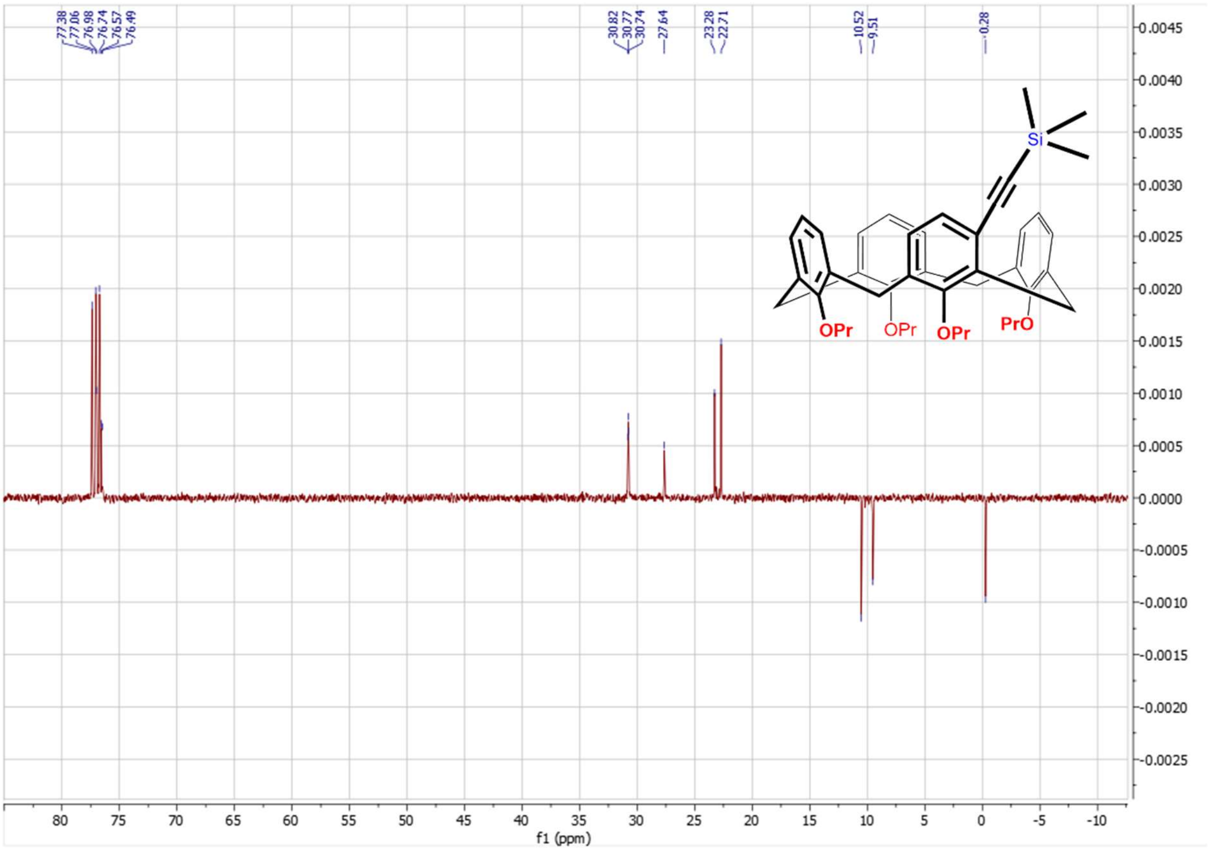

Figure S41: ^{13}C(APT) NMR of compound **5f,** aliphatic region ($CDCl_3$, 100 MHz, 298 K)

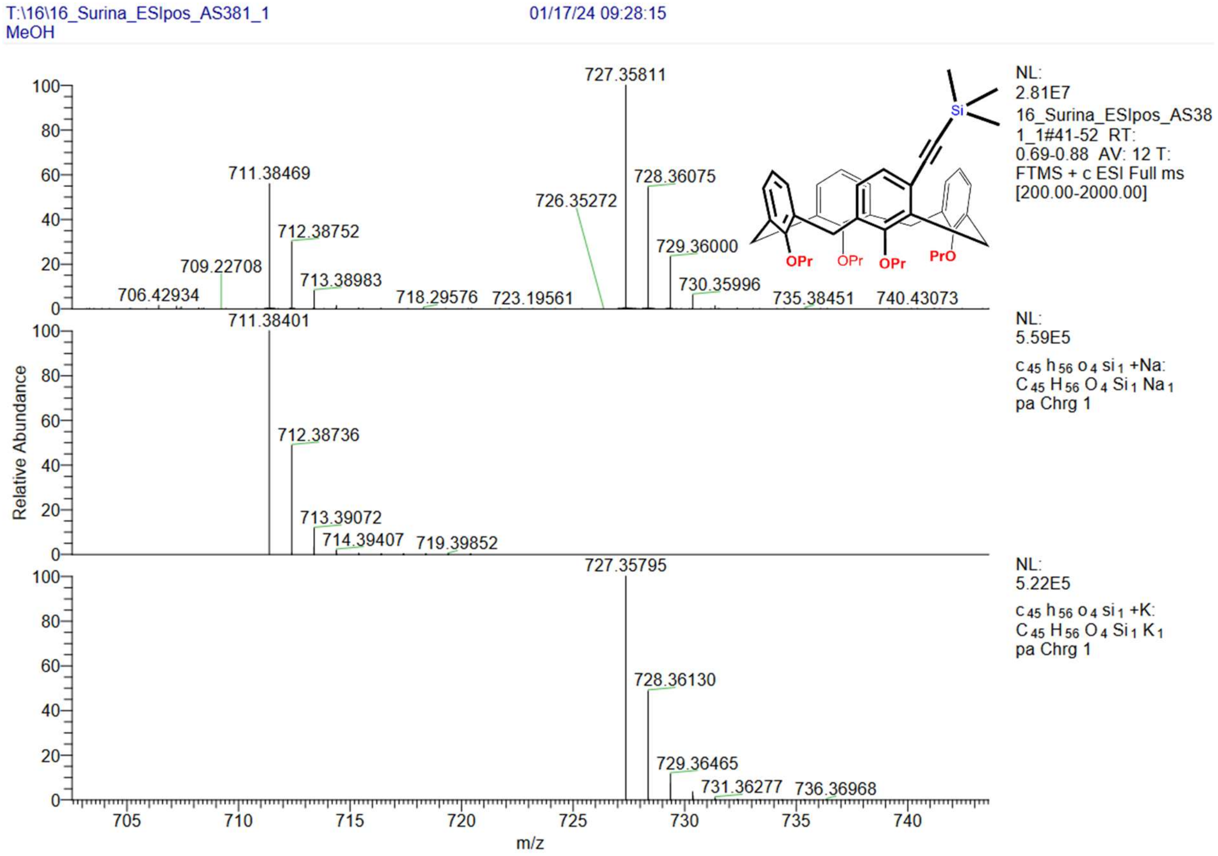

Figure S42: HRMS of compound **5f** (ESI+)

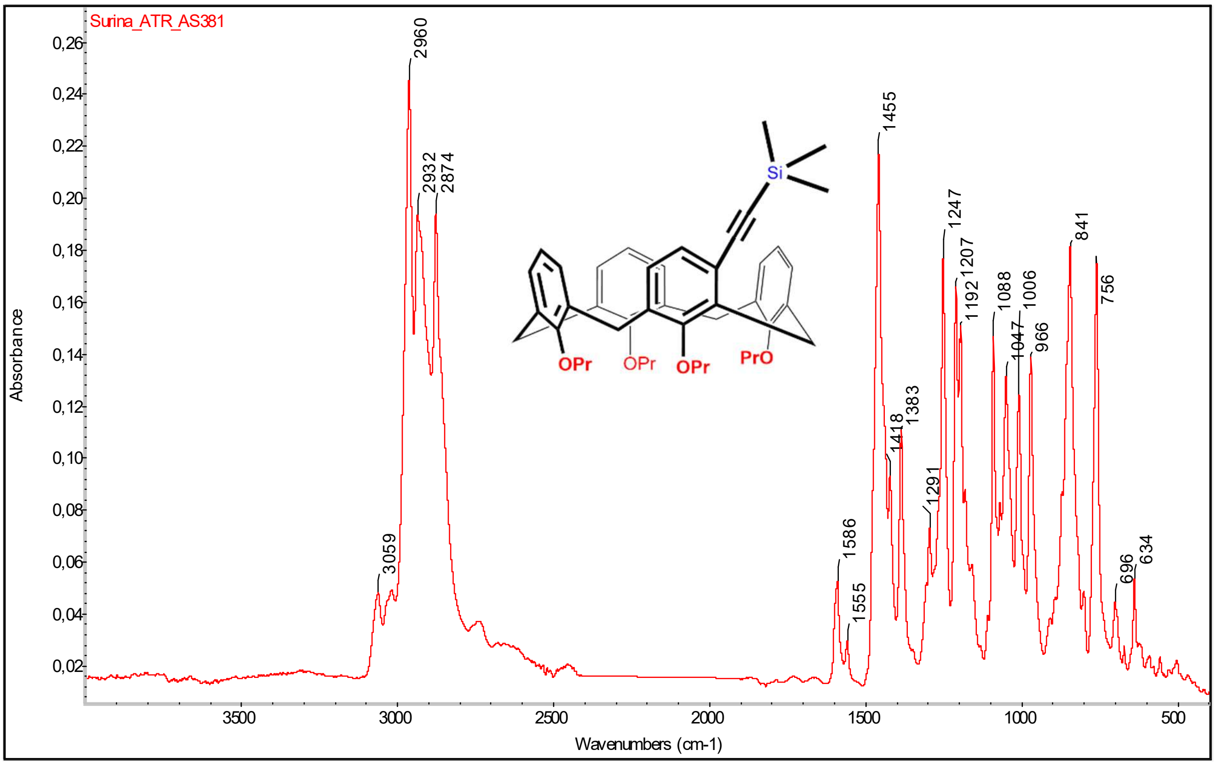

Figure S43: IR of compound **5f** (KBr)

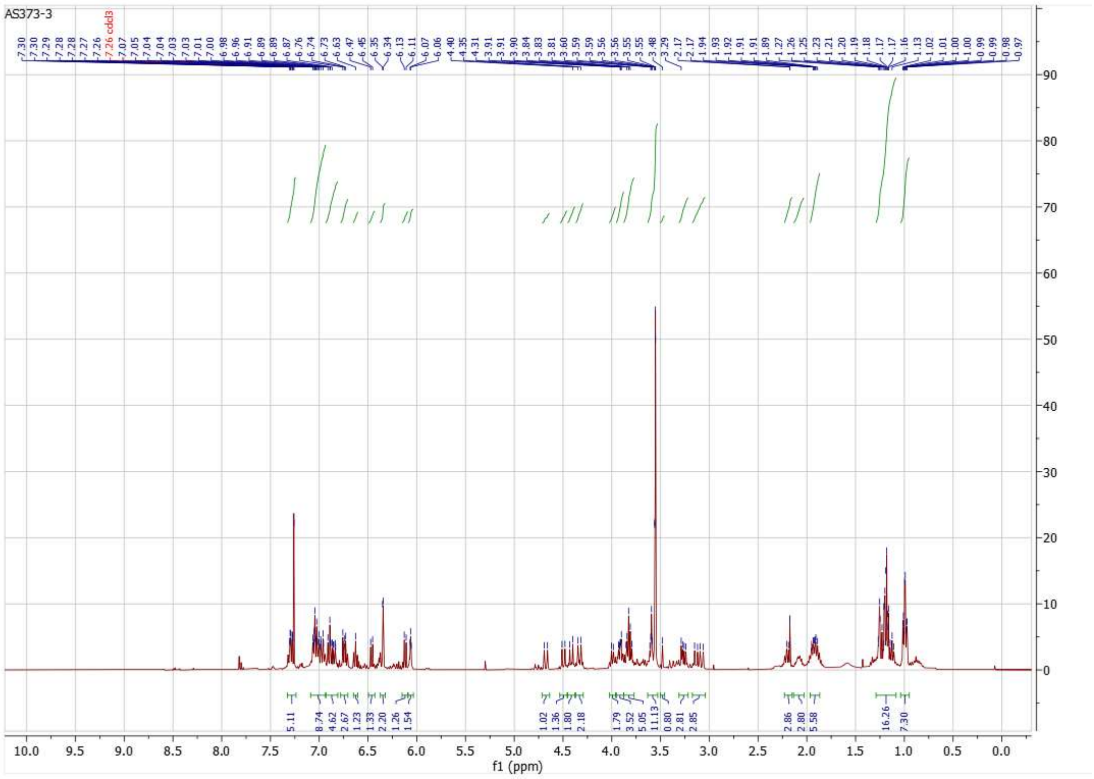

Figure S44: ^{1}H NMR of mixture **7a** with impurities. Integration cannot be used for determination of structure due to impurities. (CDCl3, 400 MHz, 298 K)

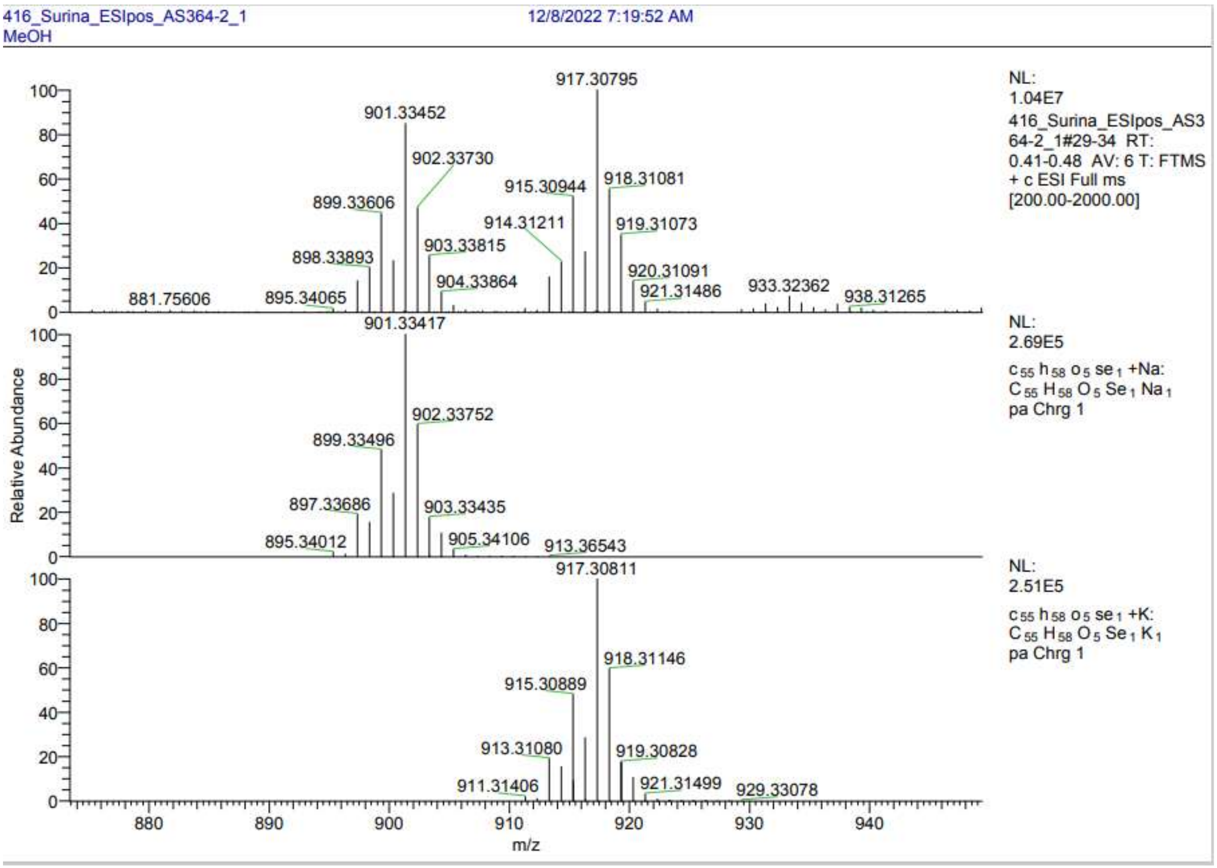

Figure S45: HRMS of compound **7a** (ESI+)

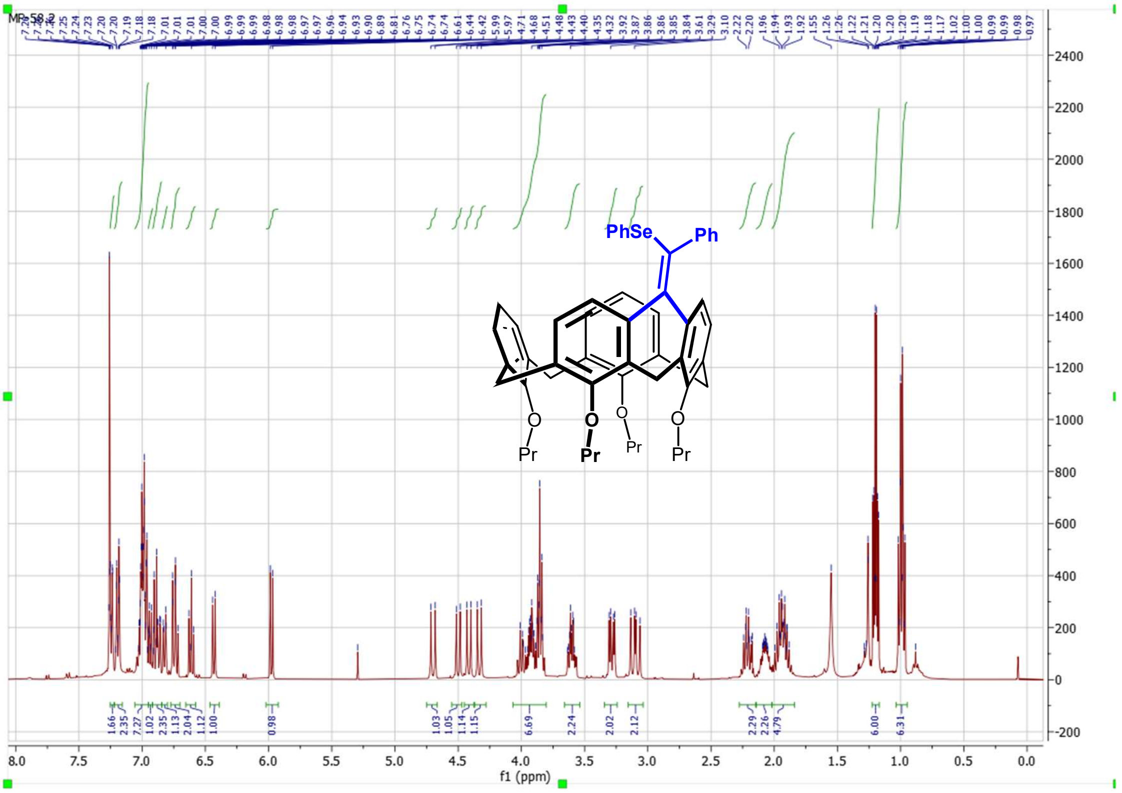

Figure S46: ^{1}H NMR of compound **7b** ($CDCl_3$, 400 MHz, 298 K)

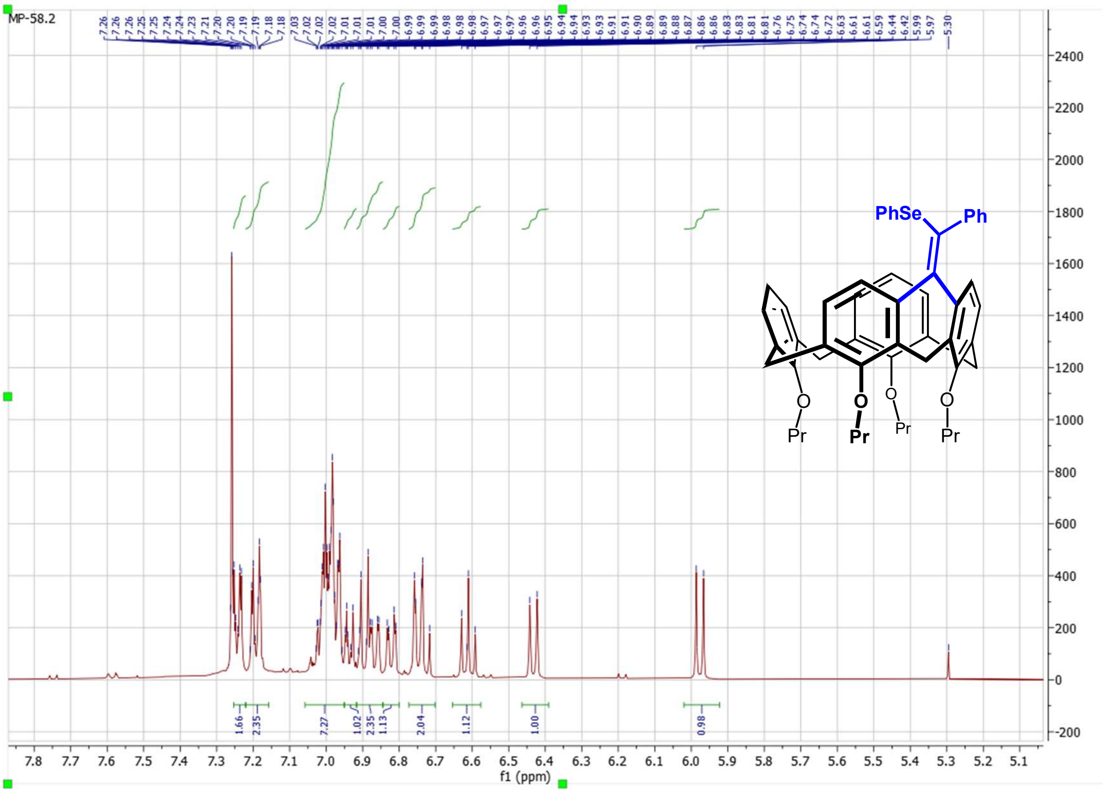

Figure S47: ^{1}H NMR of compound **7b,** aromatic region ($CDCl_3$, 400 MHz, 298 K)

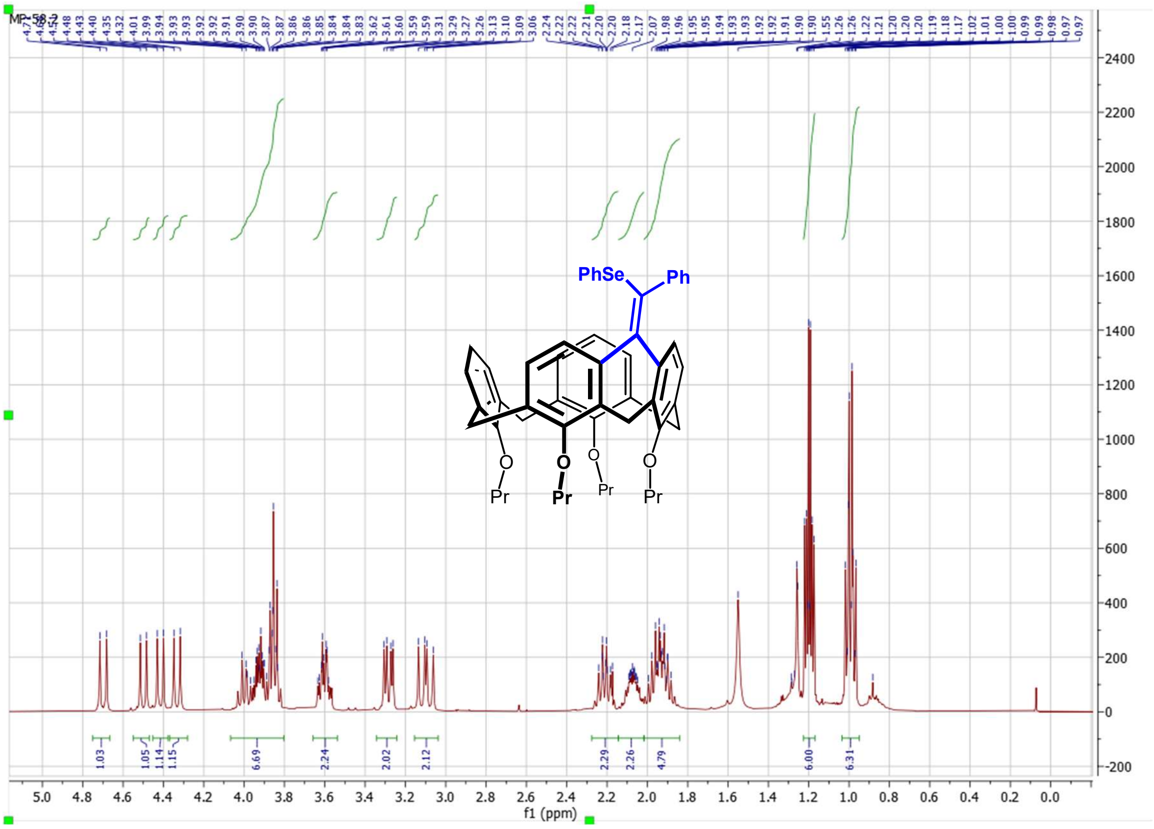

Figure S48: ^{1}H NMR of compound **7b,** aliphatic region ($CDCl_3$, 400 MHz, 298 K)

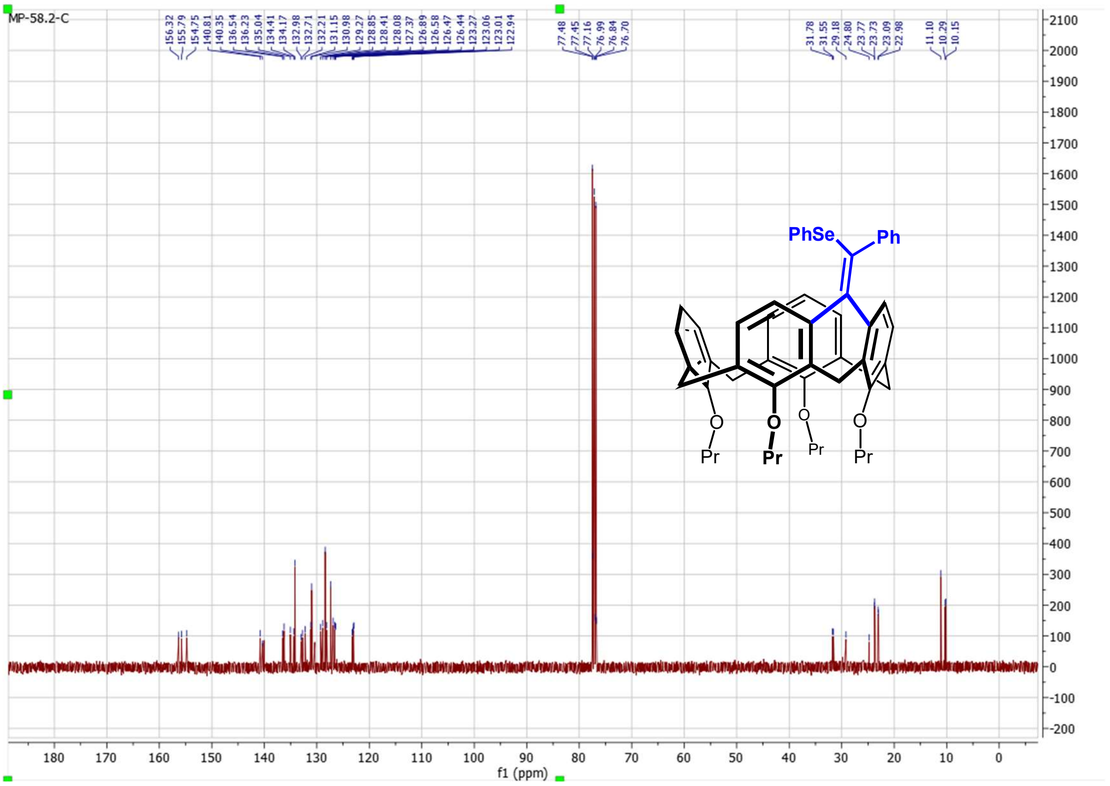

Figure S49: ^{13}C NMR of compound **7b** ($CDCl_3$, 100 MHz, 298 K)

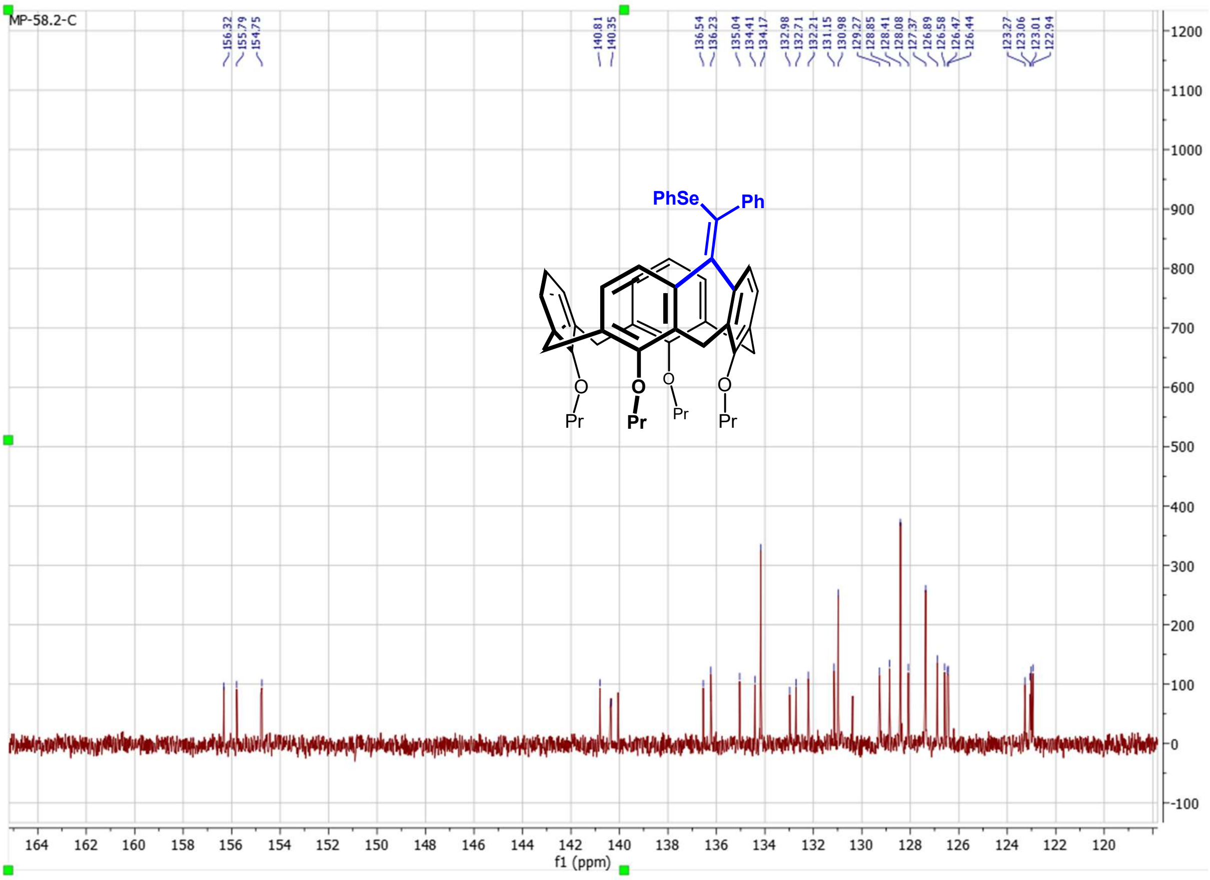

Figure S50: ^{13}C NMR of compound **7b,** aromatic region ($CDCl_3$, 100 MHz, 298 K)

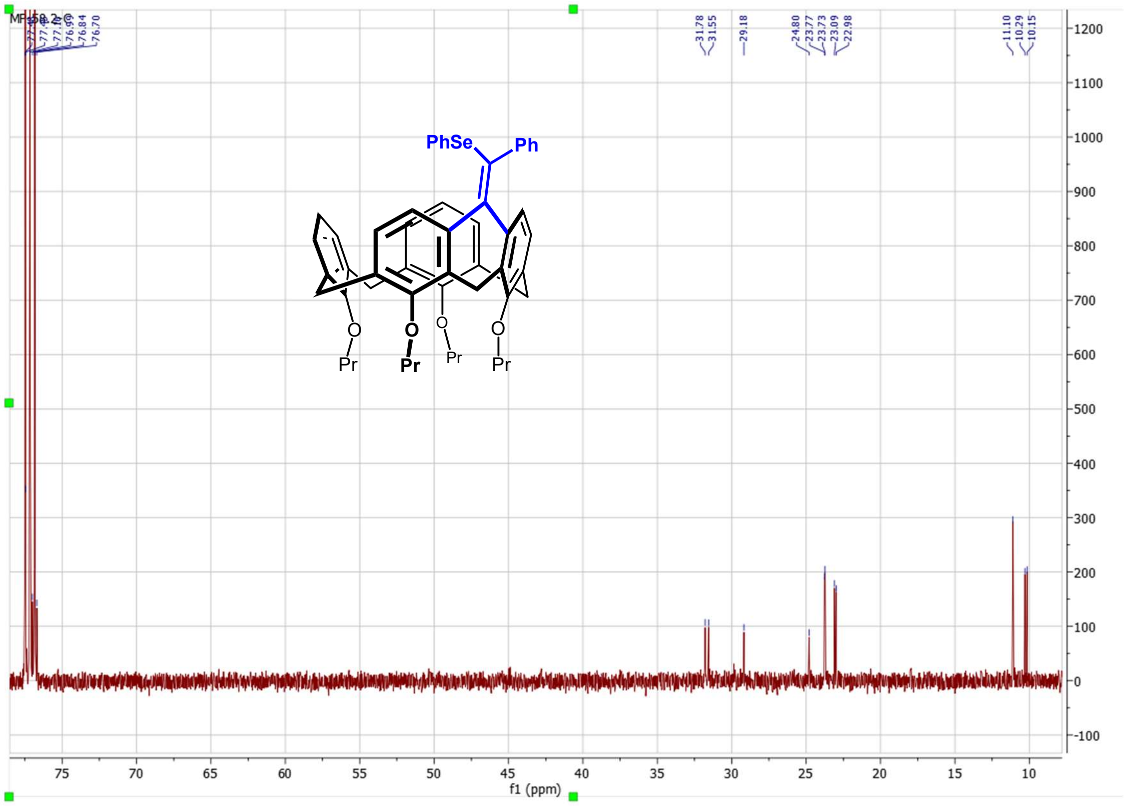

Figure S51: ^{13}C NMR of compound **7b,** aliphatic region ($CDCl_3$, 100 MHz, 298 K)

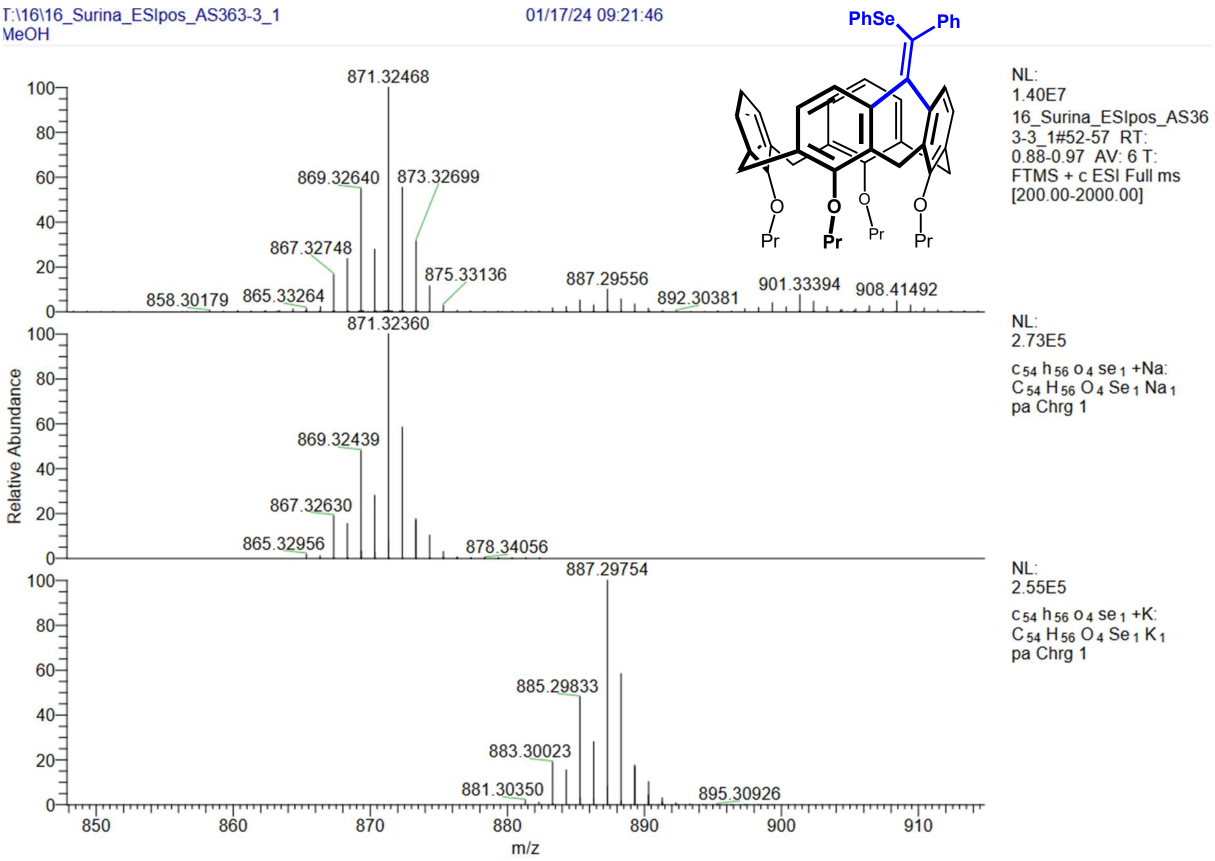

Figure S52: HRMS of compound **7b** (ESI+)

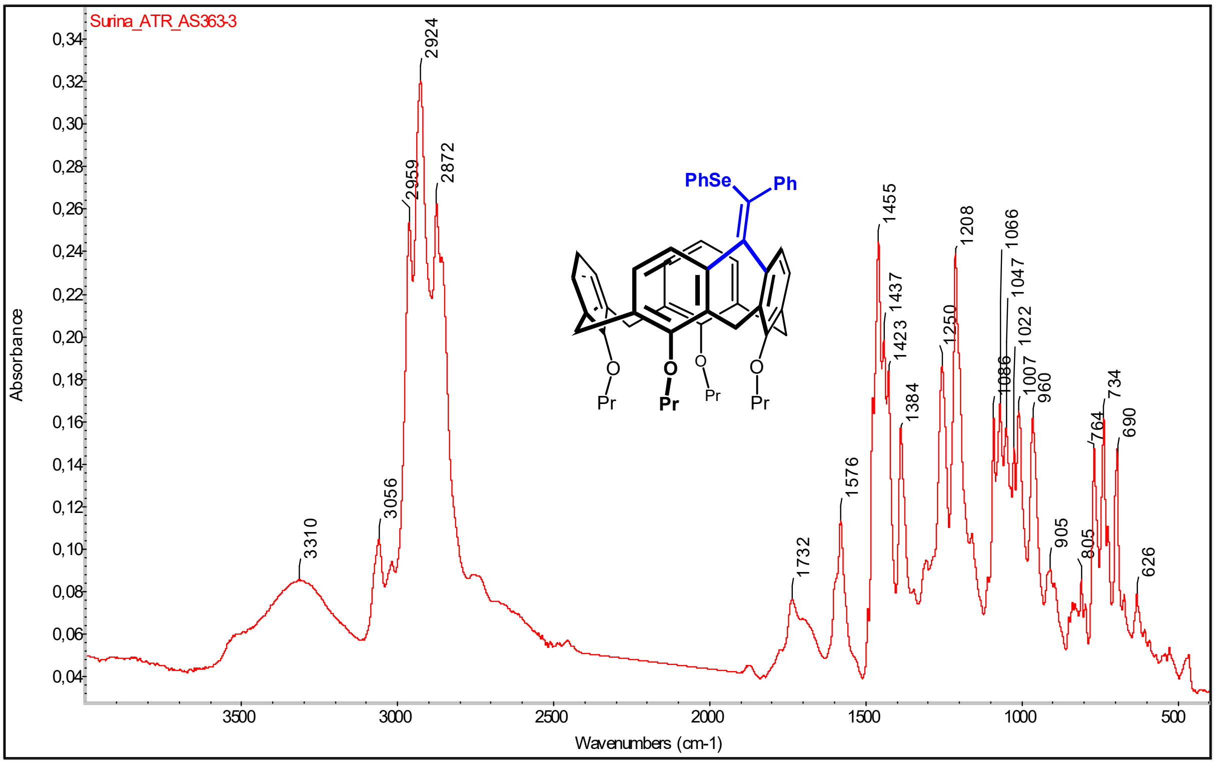

Figure S53: IR of compound **7b** (KBr)

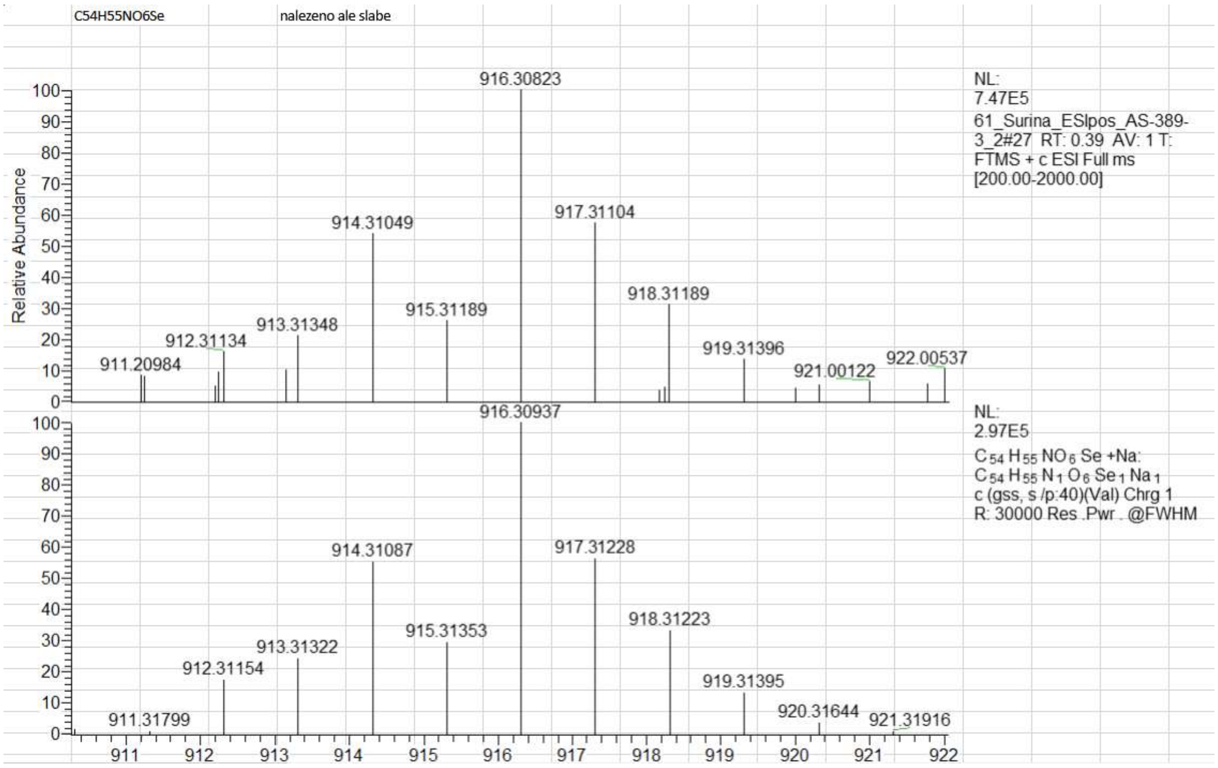

Figure S54: HRMS of compound **7d** (ESI+)

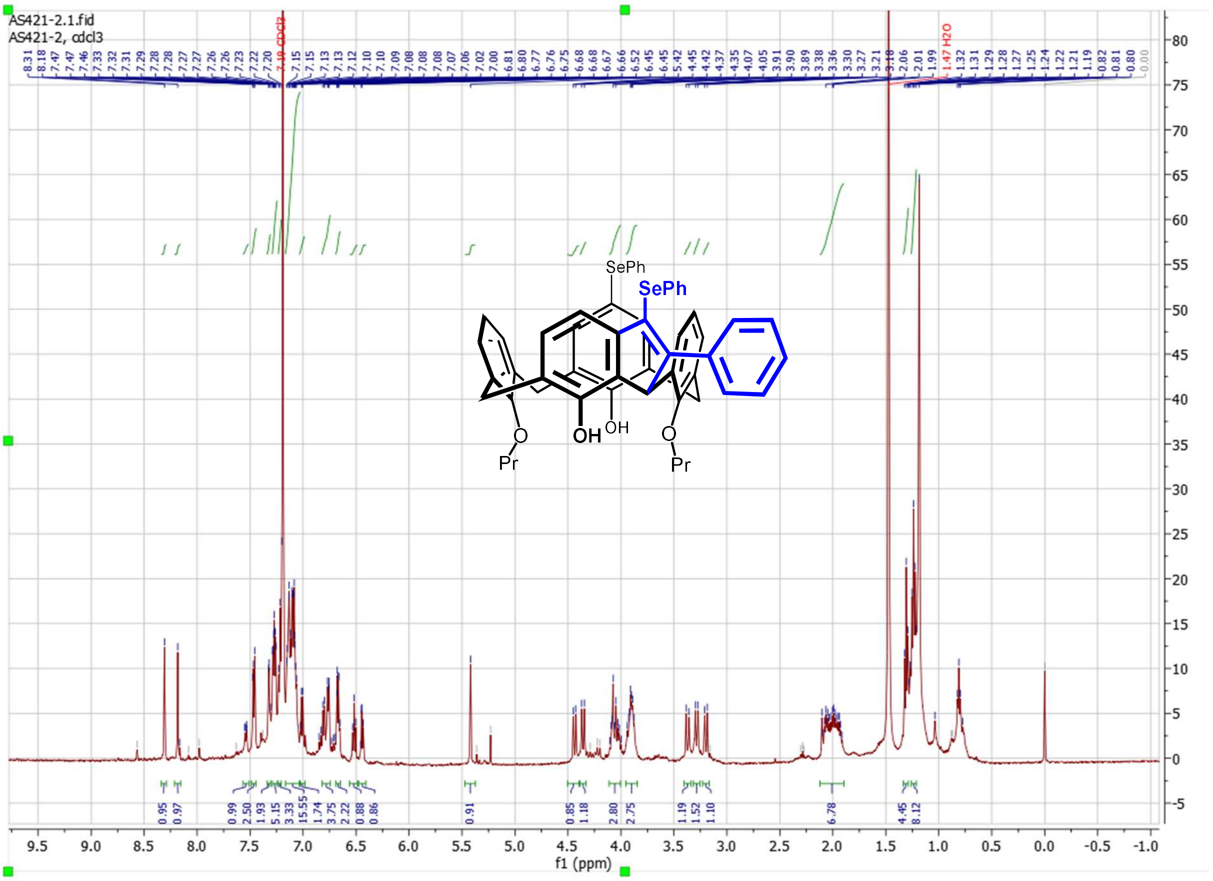

Figure S55: ^{1}H NMR of compound **8b** ($CDCl_3$, 500 MHz, 298 K)

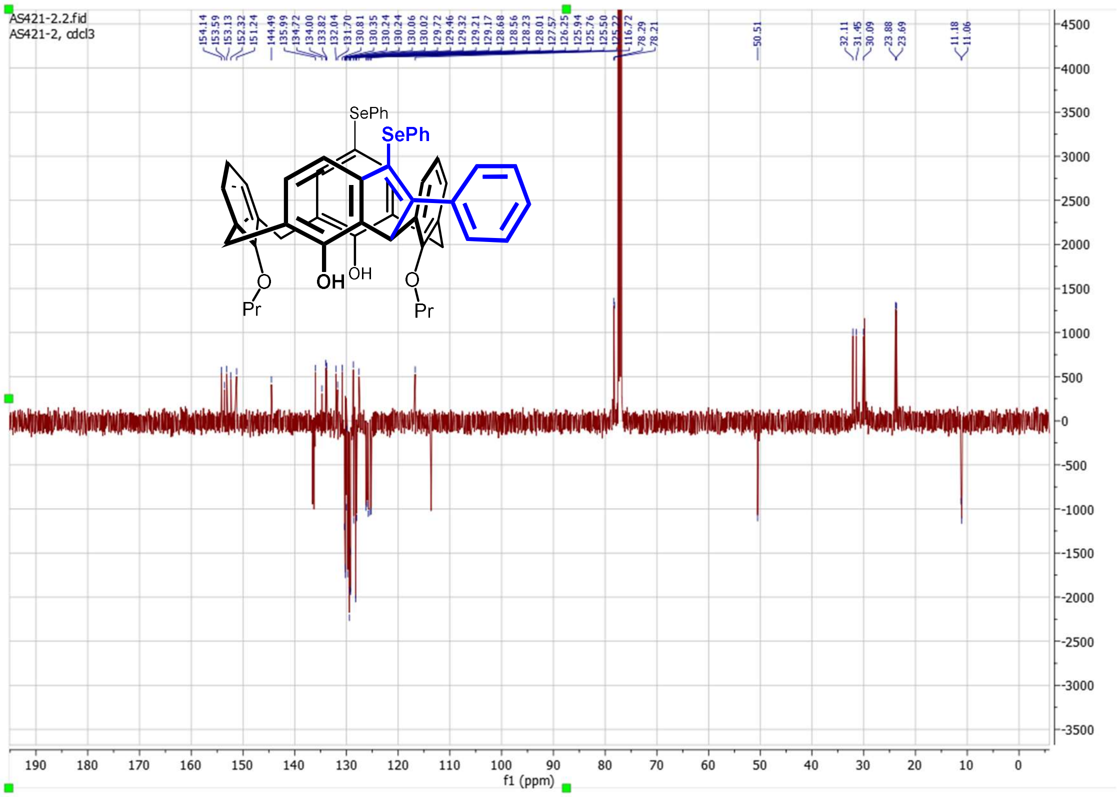

Figure S56: ^{13}C(APT) NMR of compound **8b** ($CDCl_3$, 125 MHz, 298 K)

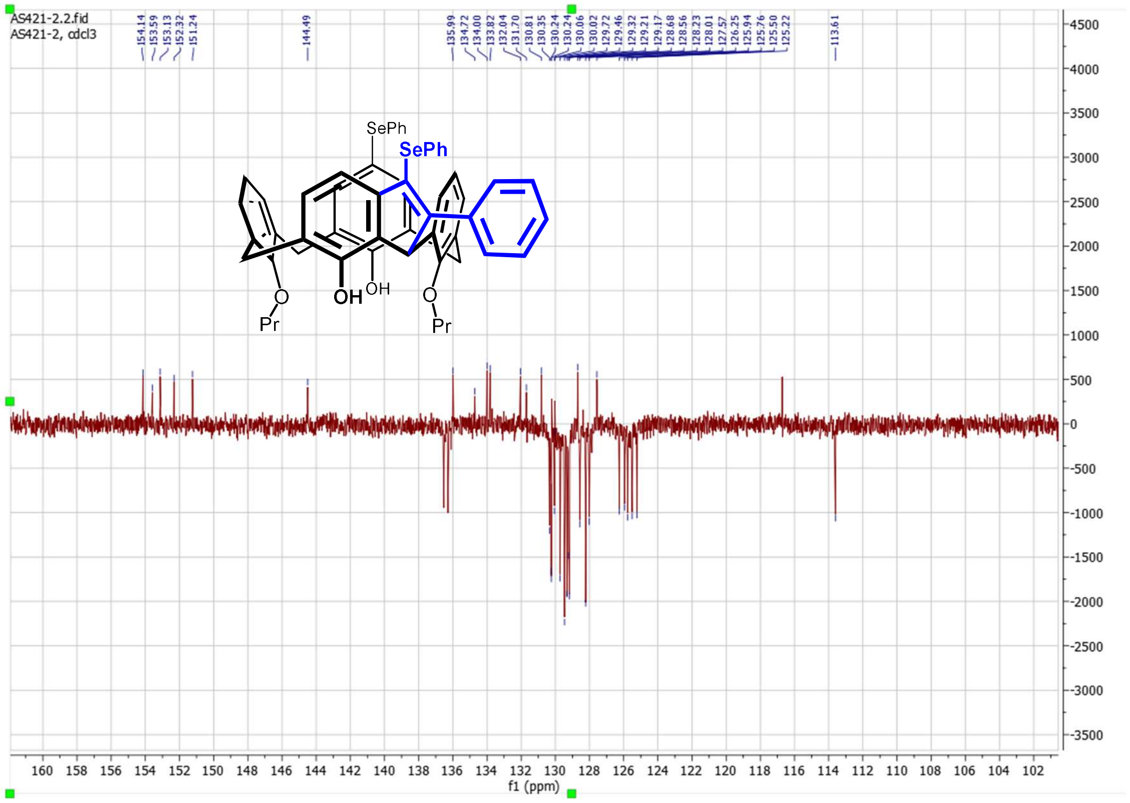

Figure S57: ^{13}C(APT) NMR of compound **8b,** aromatic region ($CDCl_3$, 125 MHz, 298 K)

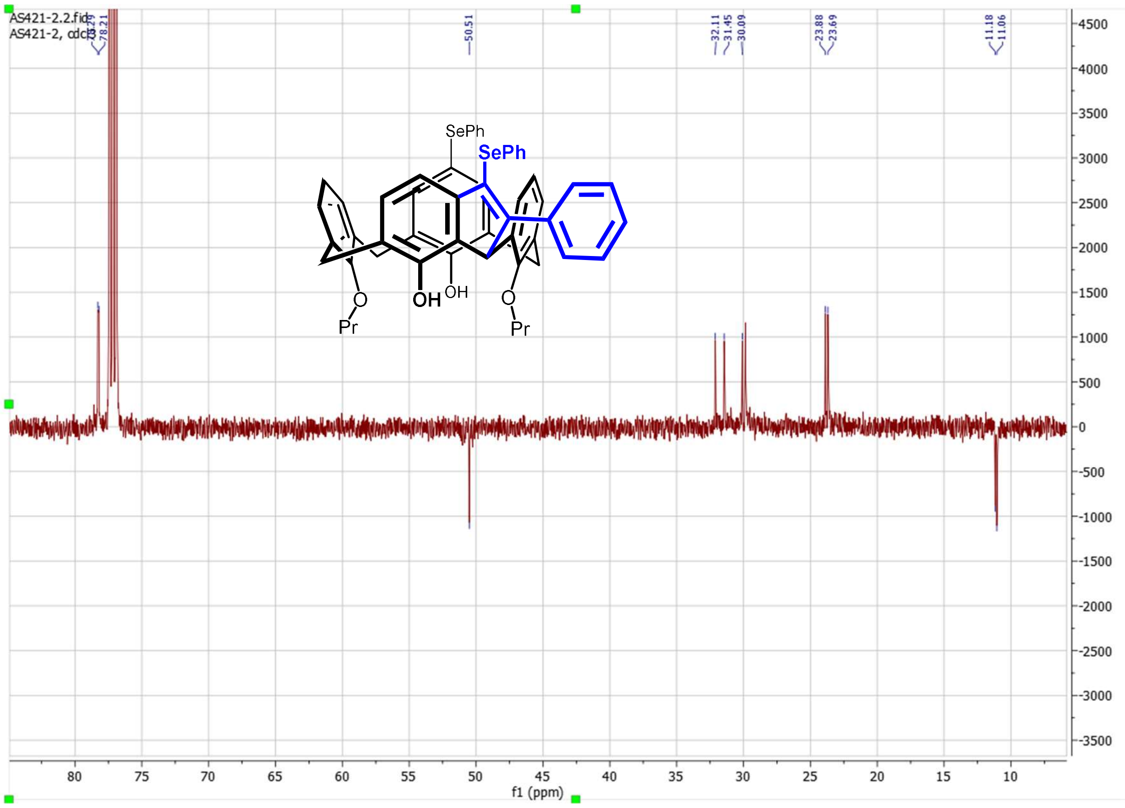

Figure S58: ^{13}C(APT) NMR of compound **8b,** aliphatic region ($CDCl_3$, 125 MHz, 298 K)

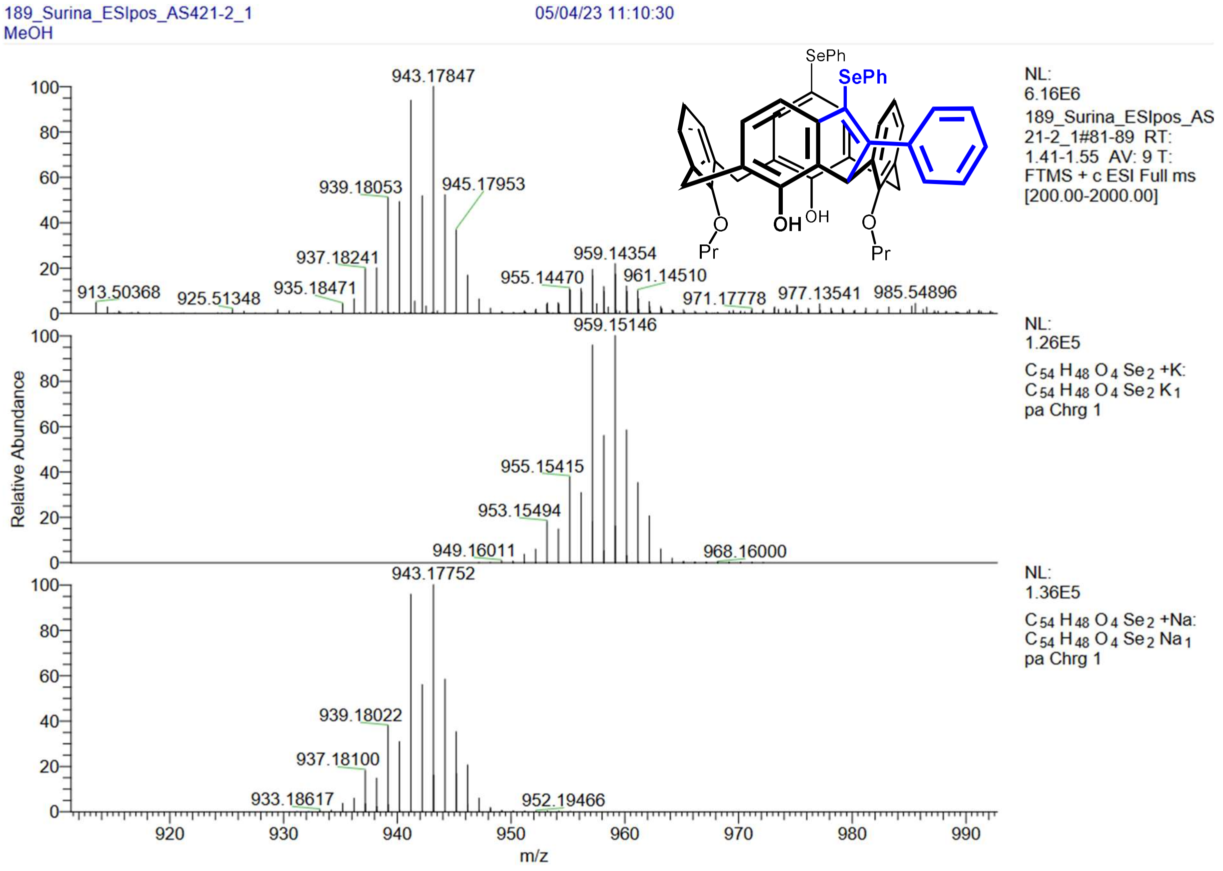

Figure S59: HRMS of compound **8b** (ESI$^+$)

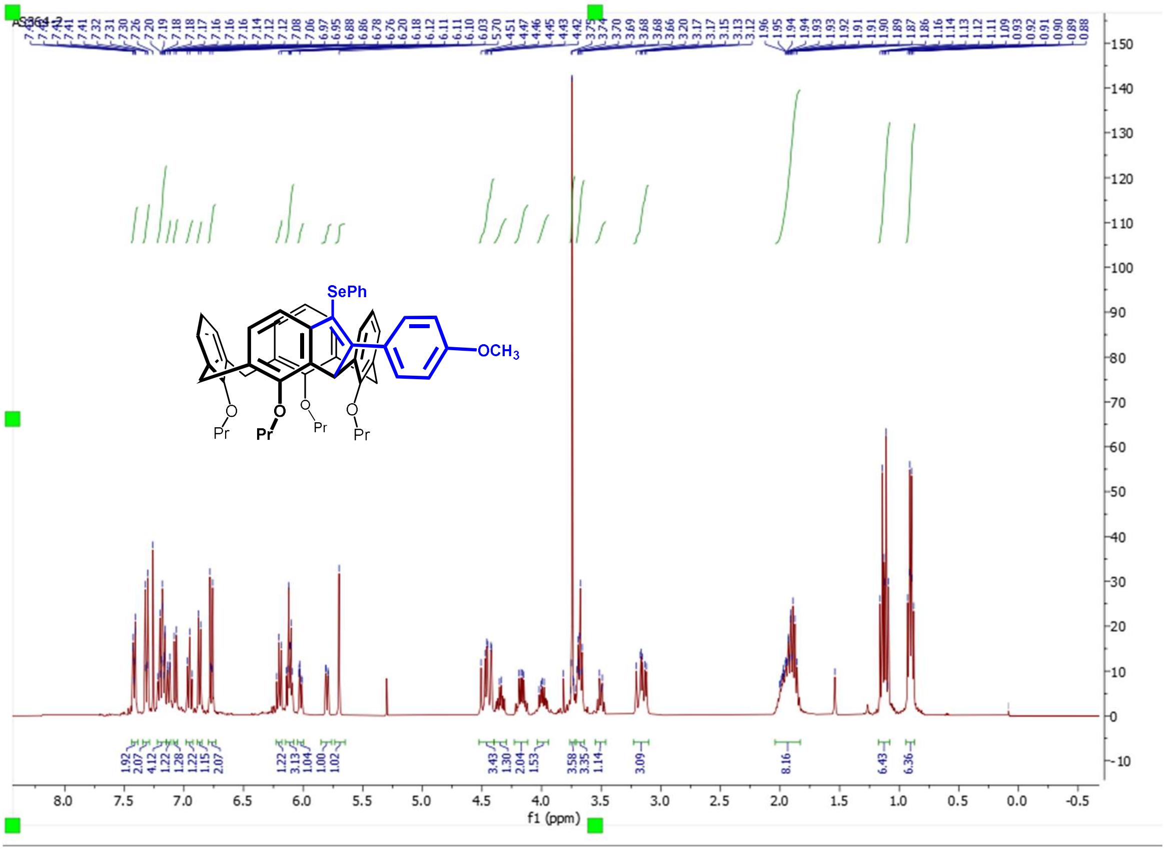

Figure S60: ^{1}H NMR of compound **9c** ($CDCl_3$, 400 MHz, 298 K)

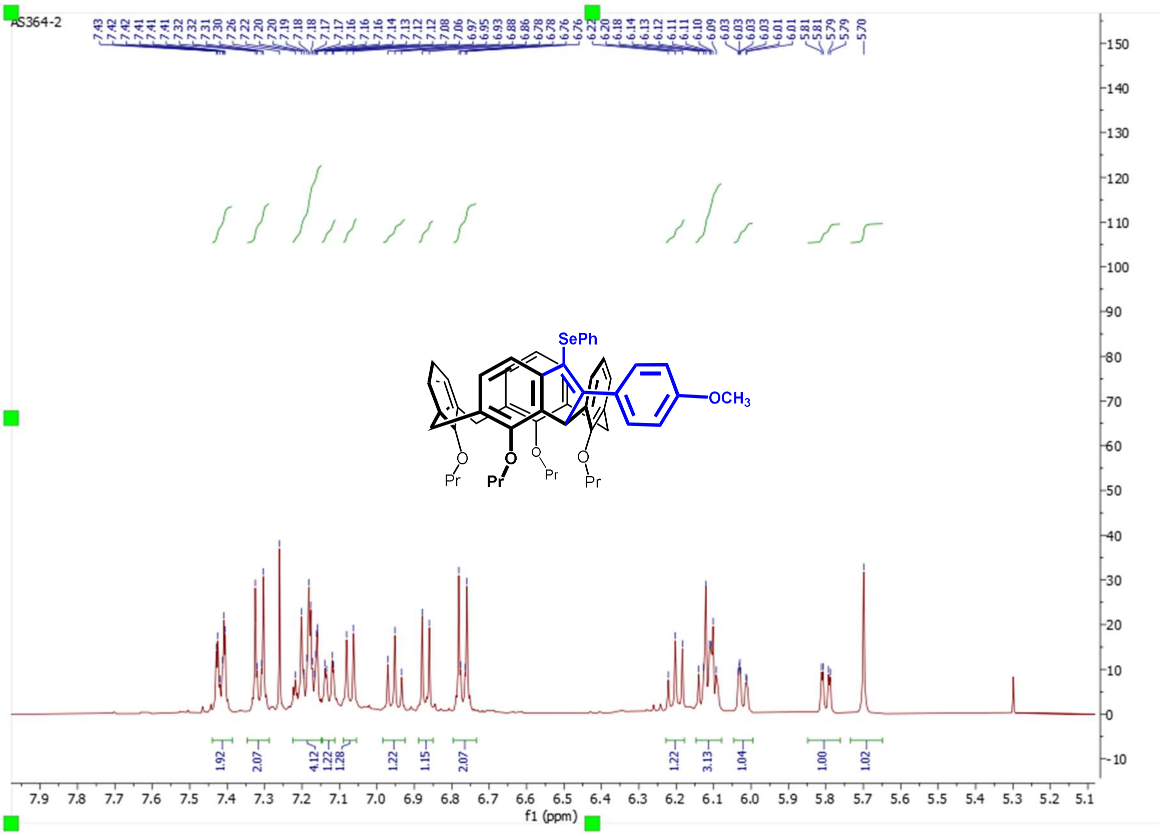

Figure S61: ^{1}H NMR of compound **9c,** aromatic region ($CDCl_3$, 400 MHz, 298 K)

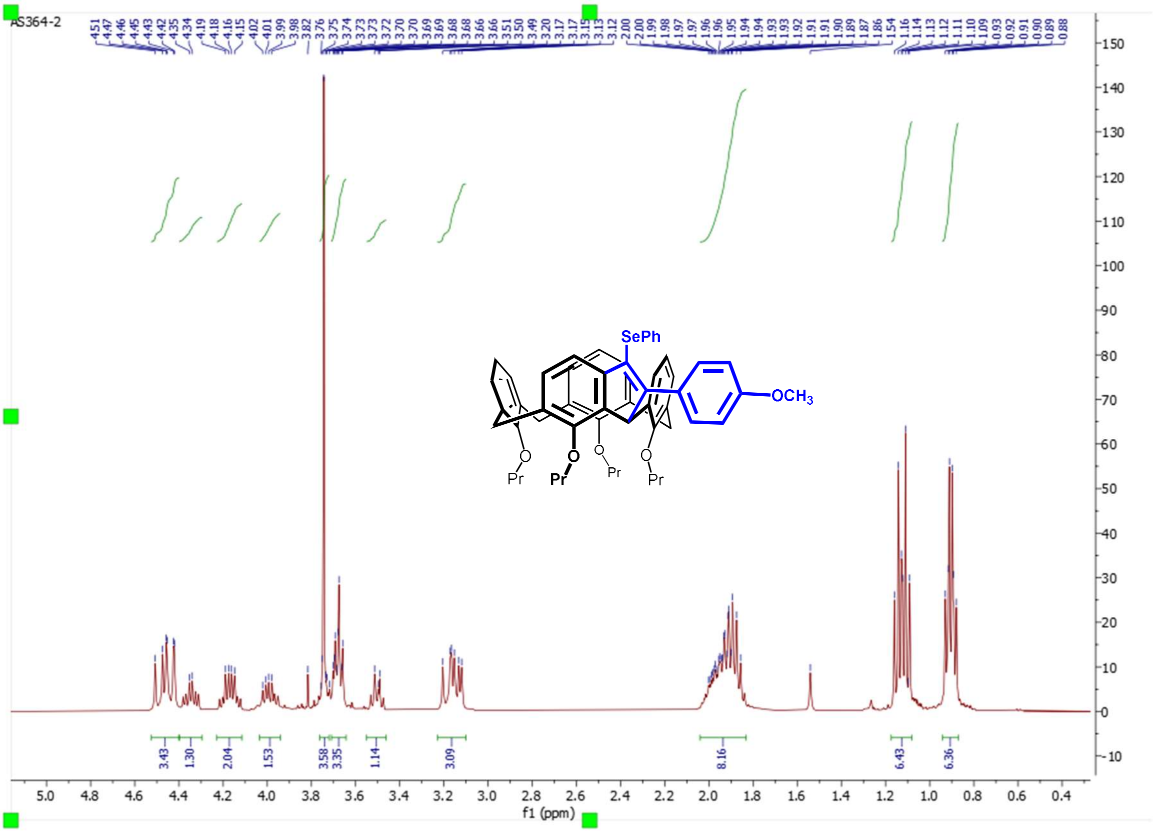

Figure S62: ^{1}H NMR of compound **9c,** aliphatic region ($CDCl_3$, 400 MHz, 298 K)

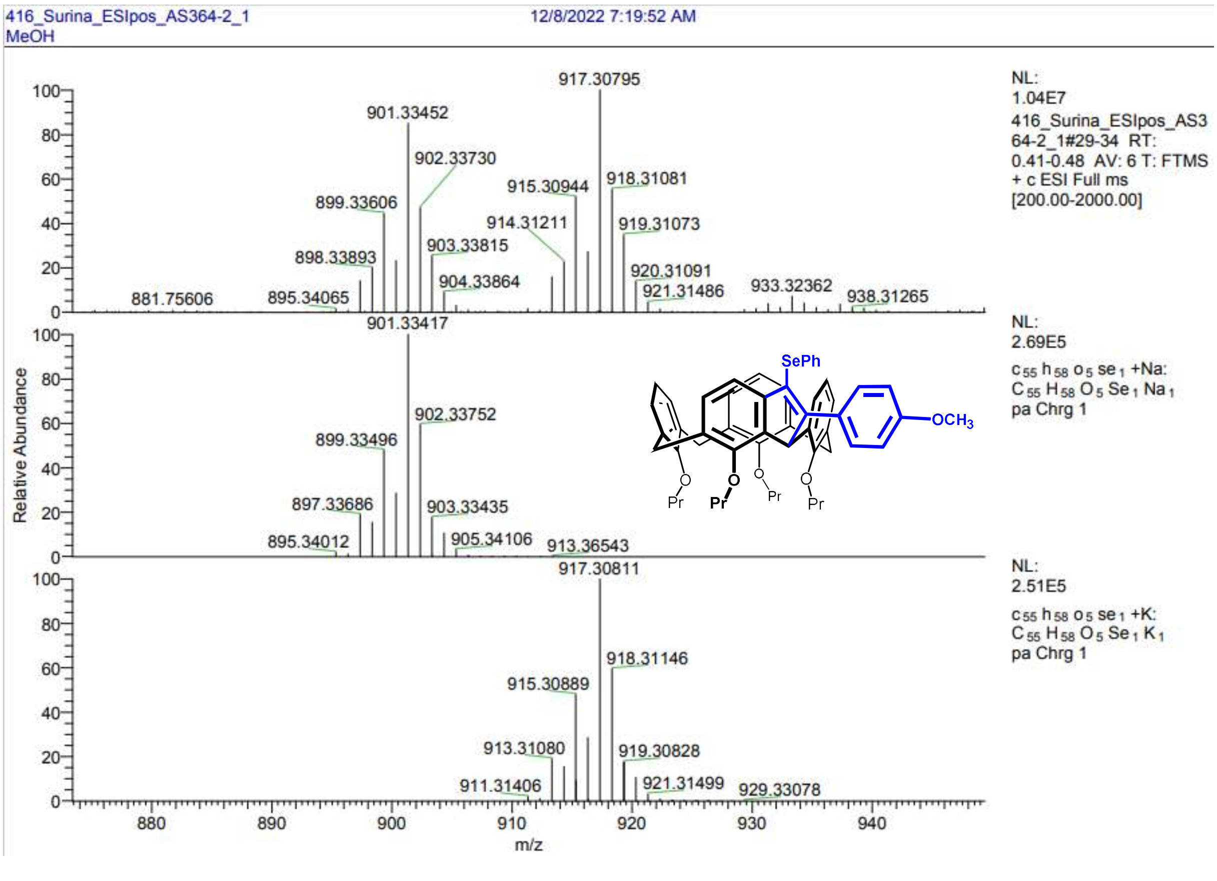

Figure S63: HRMS of compound **9c** (ESI^+)

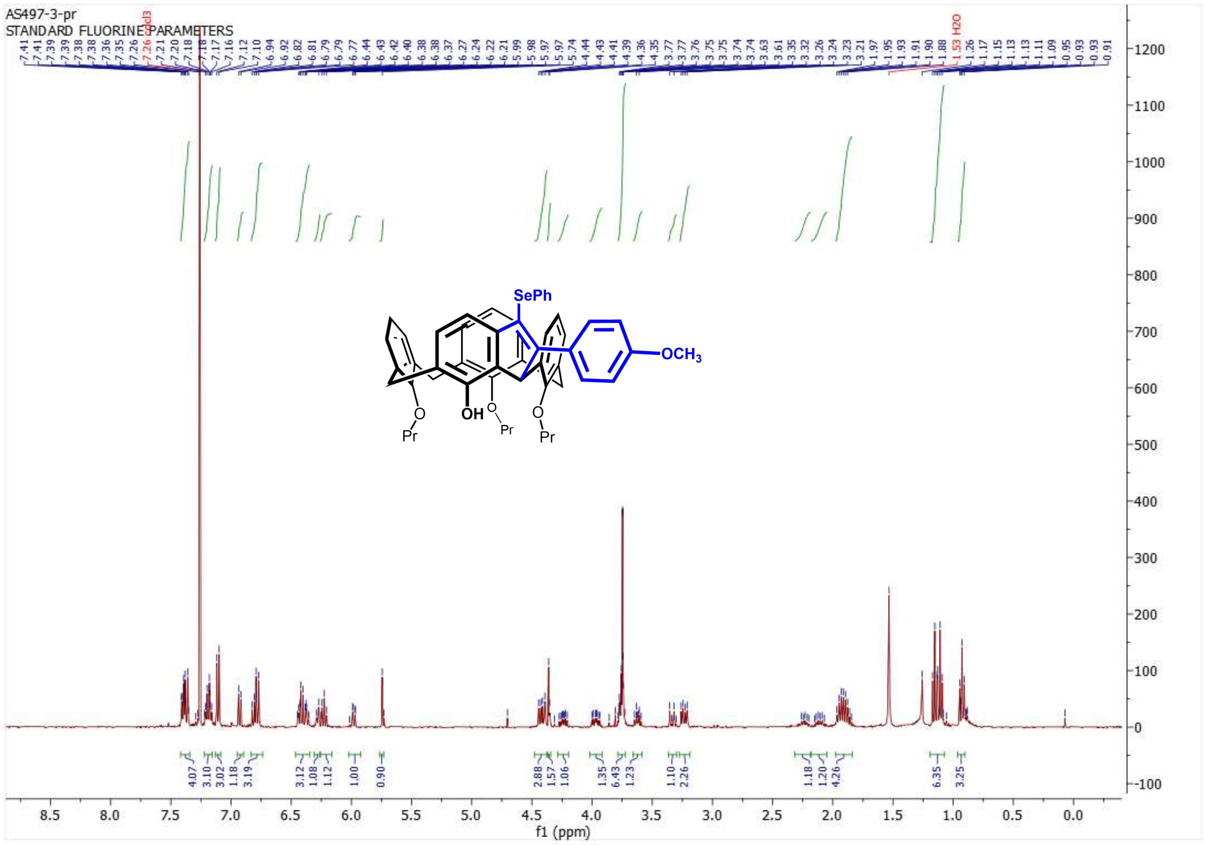

Figure S64: ^{1}H NMR of compound **10c** (CDCl3, 400 MHz, 298 K)

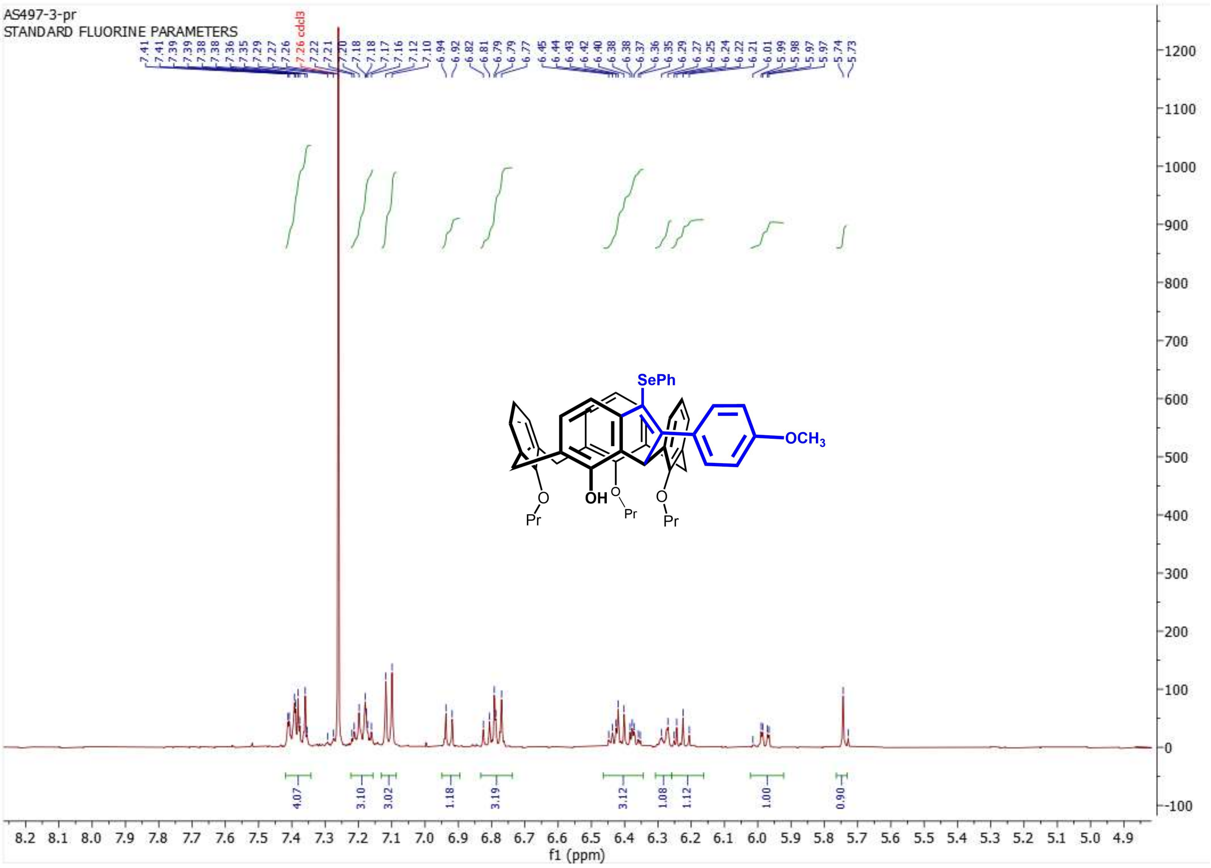

Figure S65: ^{1}H NMR of compound **10c,** aromatic region (CDCl3, 400 MHz, 298 K)

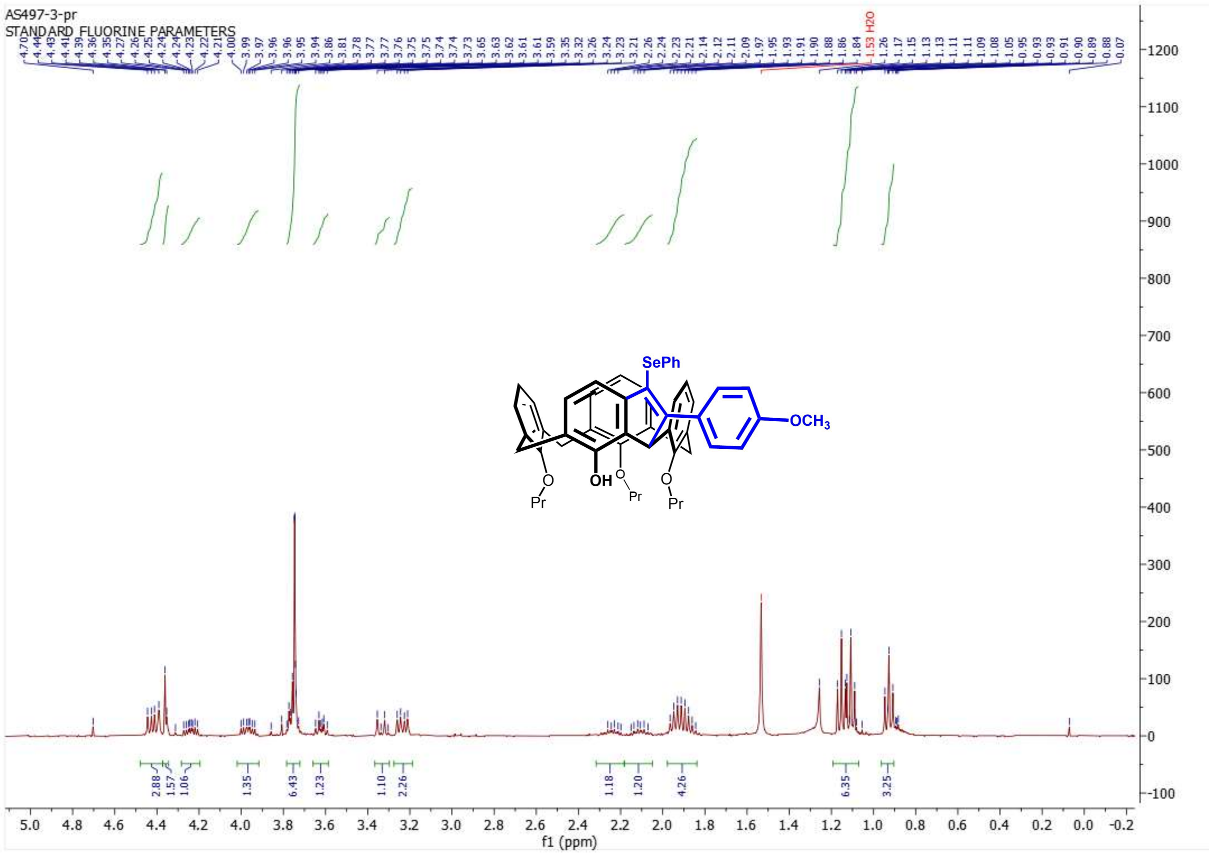

Figure S66: ^{1}H NMR of compound **10c,** aliphatic region ($CDCl_3$, 400 MHz, 298 K)

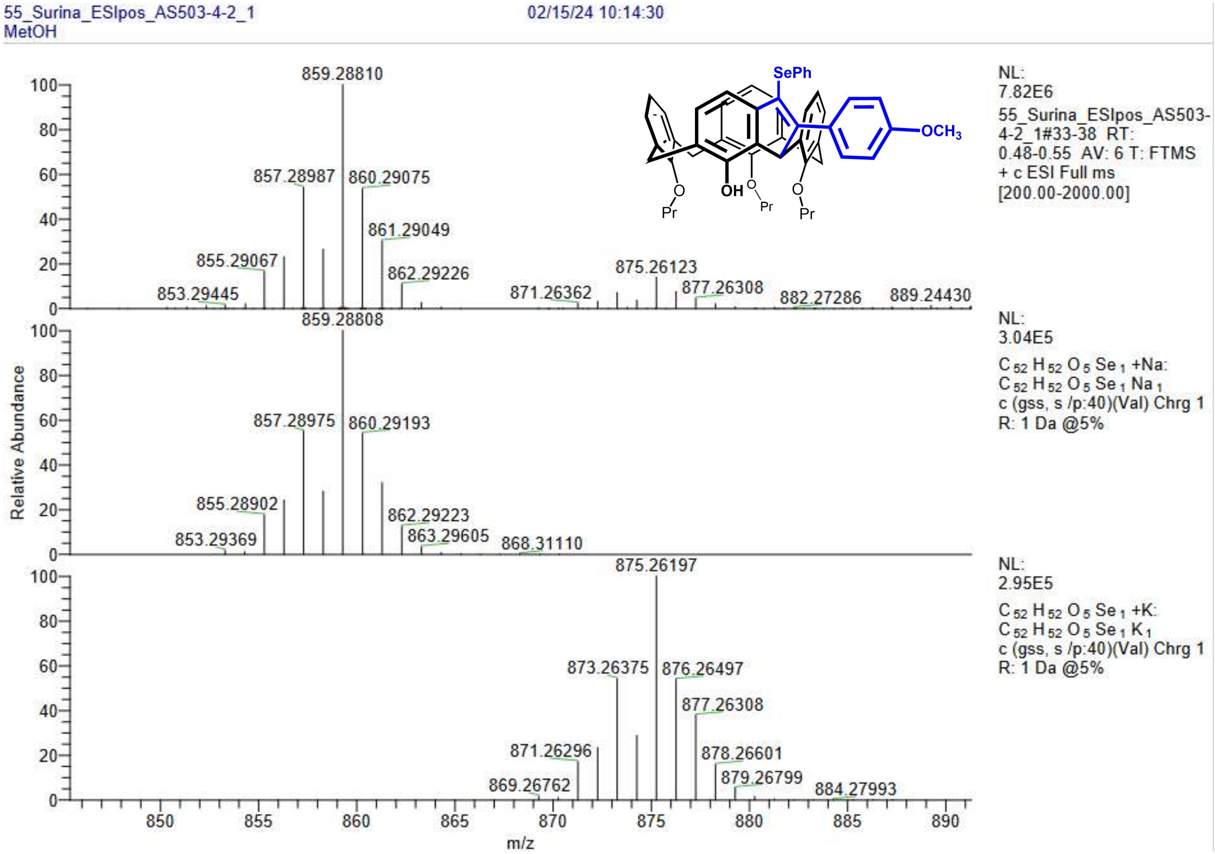

Figure S67: HRMS of compound **10c** (ESI[+])

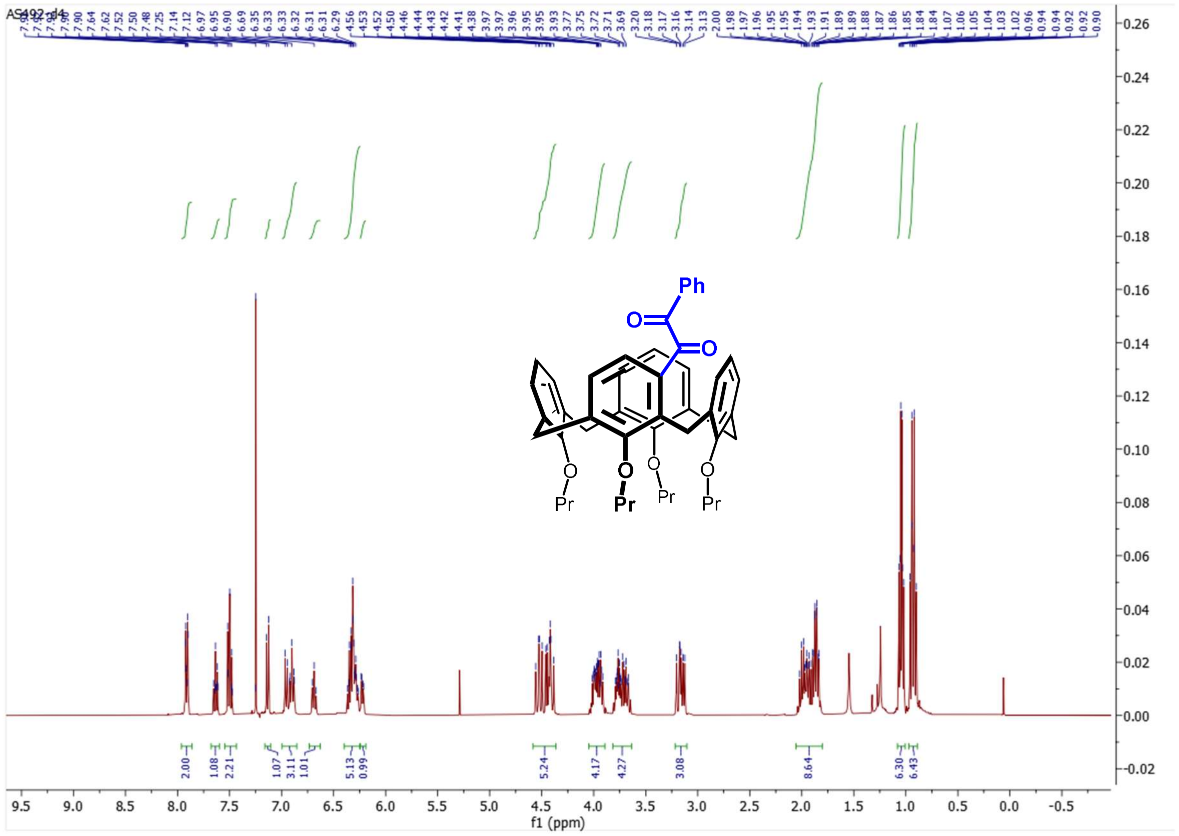

Figure S68: ^{1}H NMR of compound **11** ($CDCl_3$, 400 MHz, 298 K)

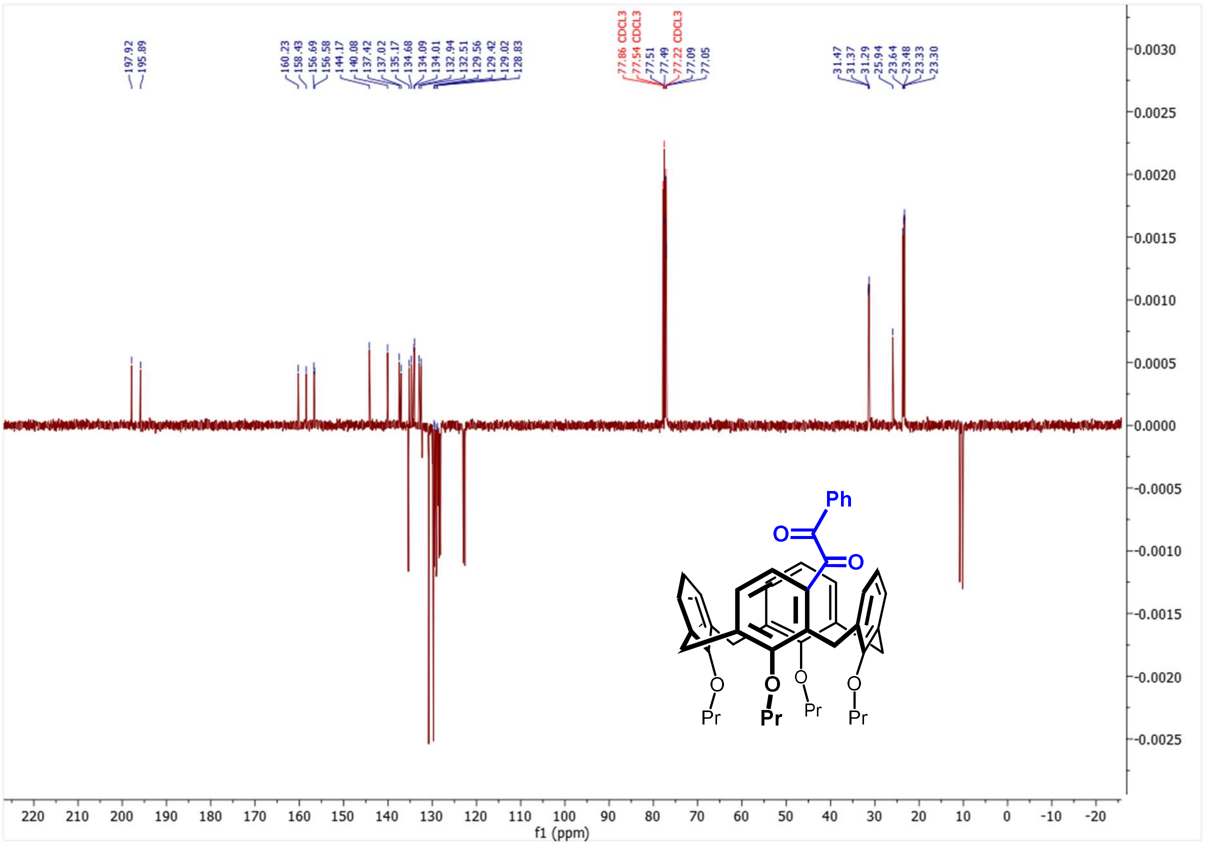

Figure S69: ^{13}C(APT) NMR of compound **11** ($CDCl_3$, 100 MHz, 298 K)

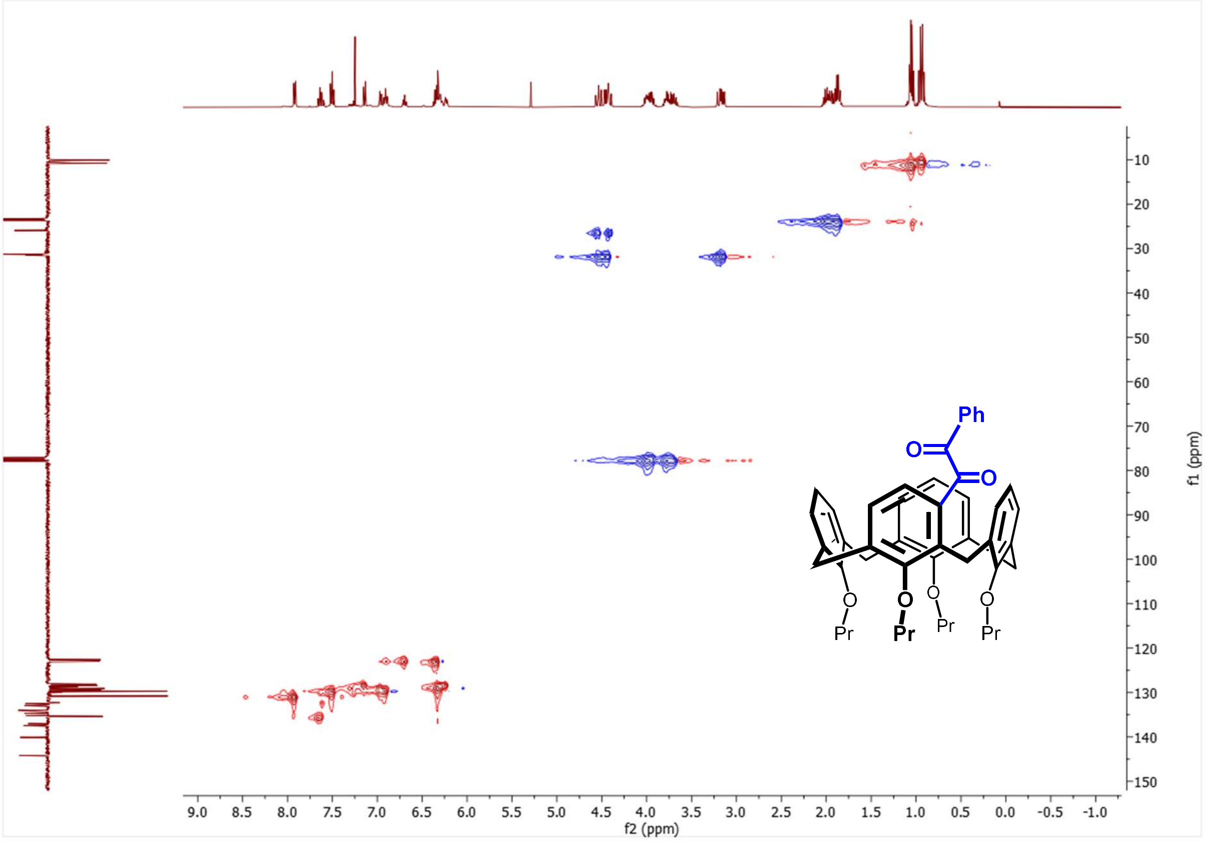

Figure S70: HSQC NMR of compound **11** ($CDCl_3$, 100 MHz, 298 K)

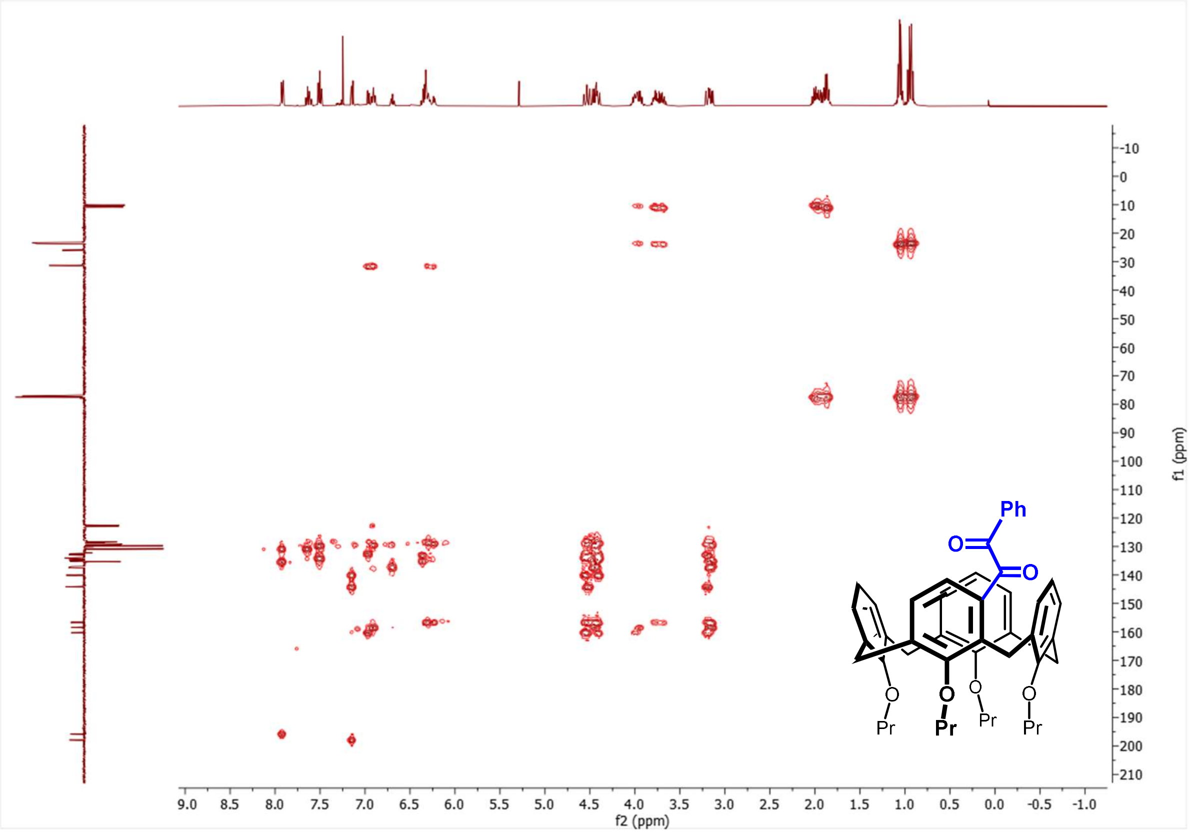

Figure S71: HMBC NMR of compound **11** ($CDCl_3$, 100 MHz, 298 K)

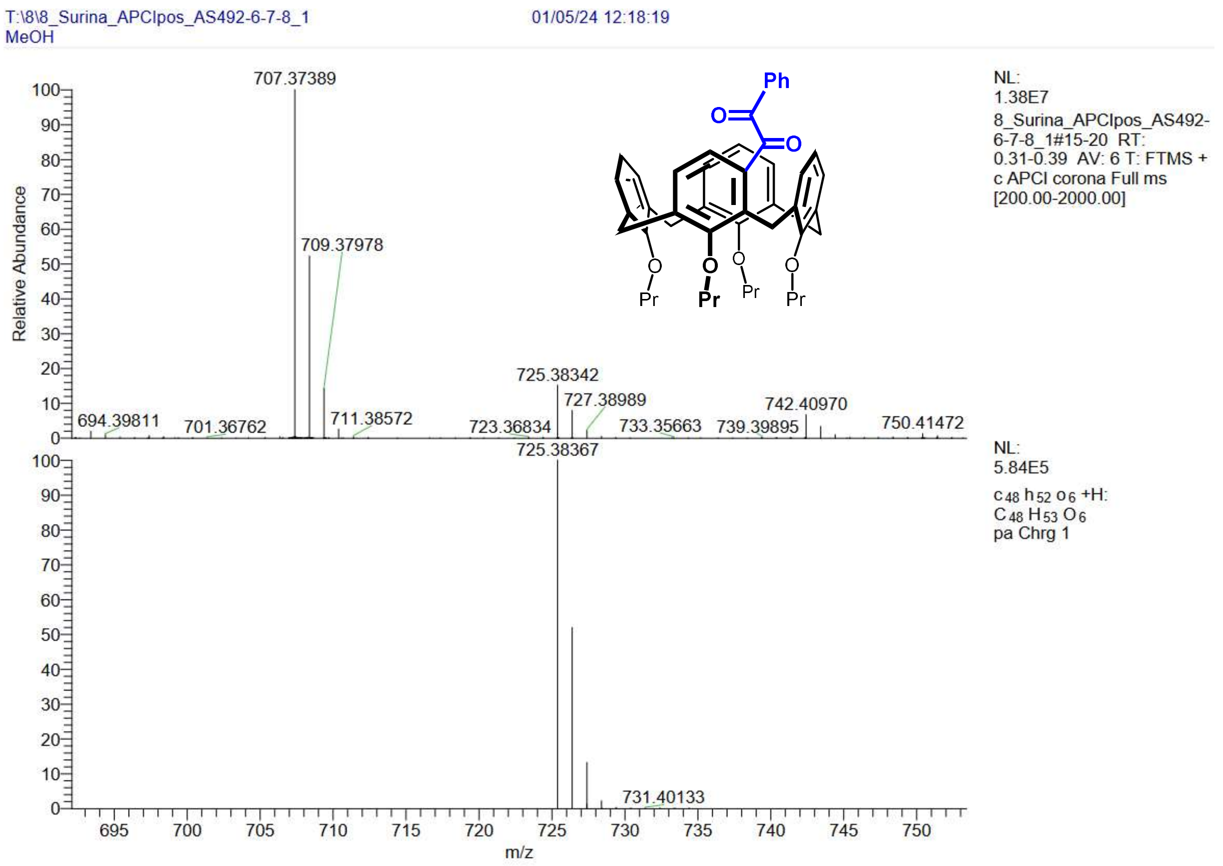

Figure S72: HRMS of compound **11** + H⁺ (ESI⁺)

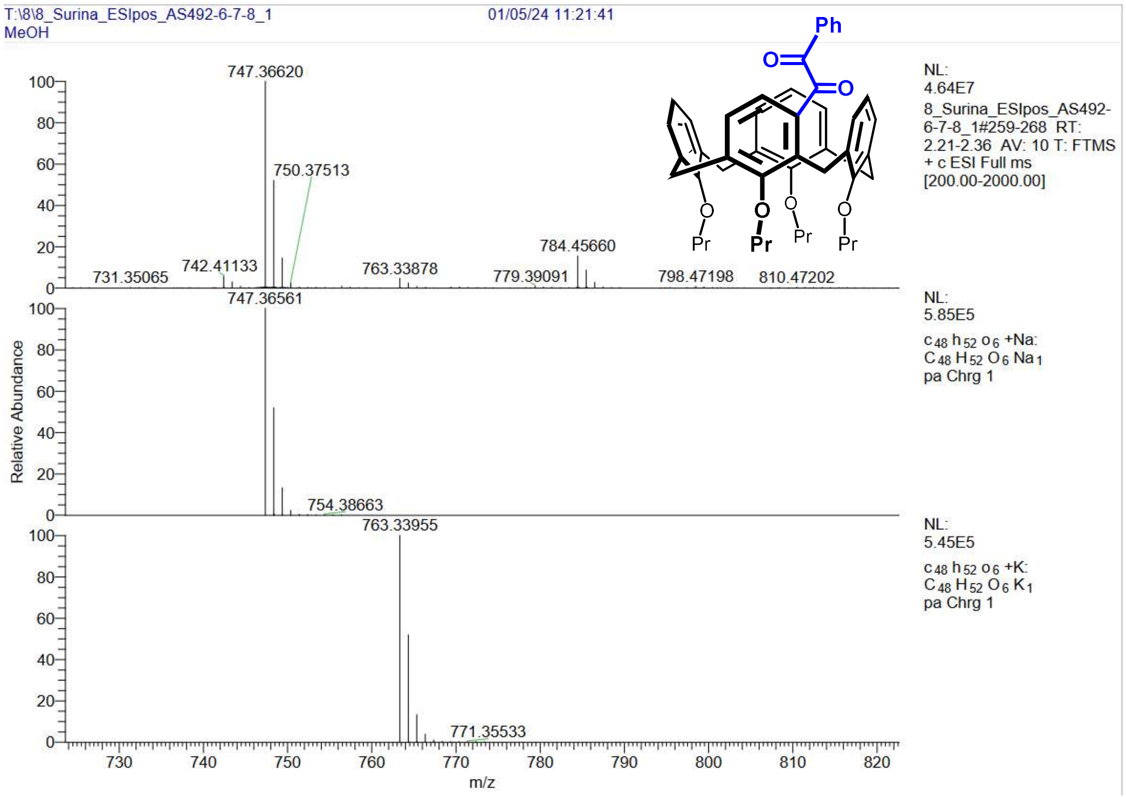

Figure S73: HRMS of compound **11** (ESI⁺)

2. Crystallographic data

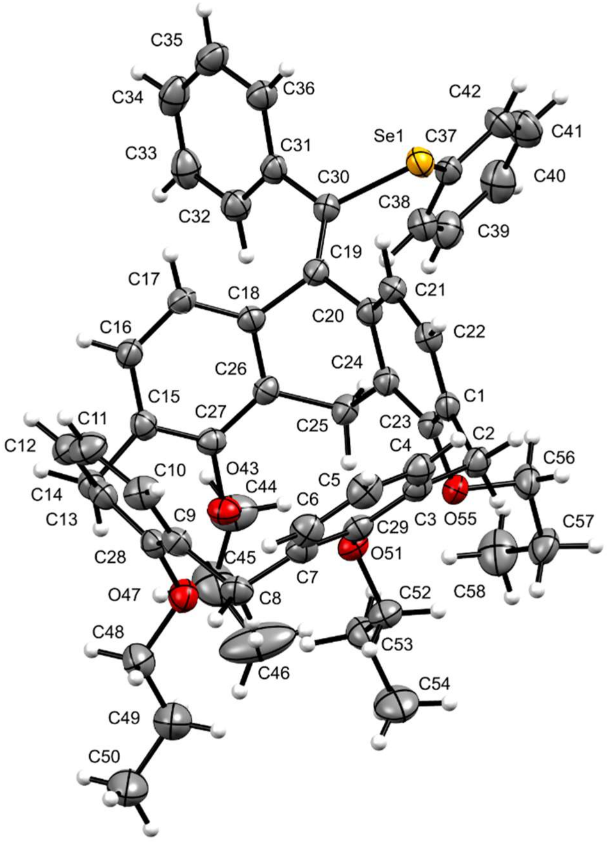

Figure S74: Numbering scheme of crystal structure of **7b**, the ORTEP diagram with 50% probability.

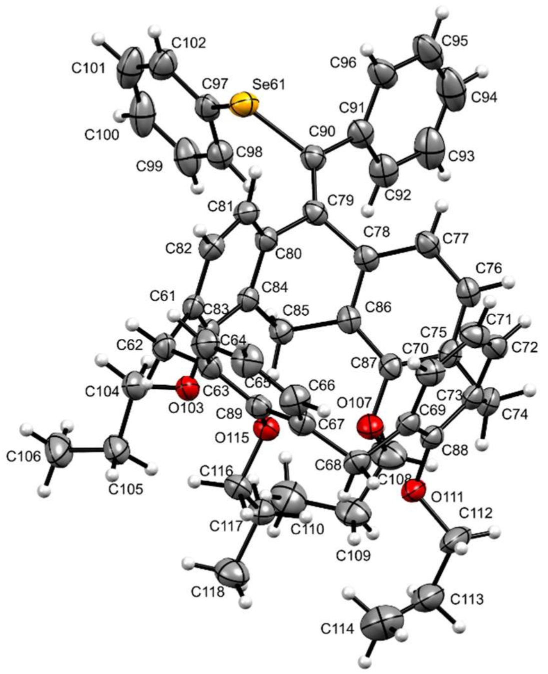

Figure S75: Numbering scheme of crystal structure of **7b** (different view), the ORTEP diagram with 50% probability.

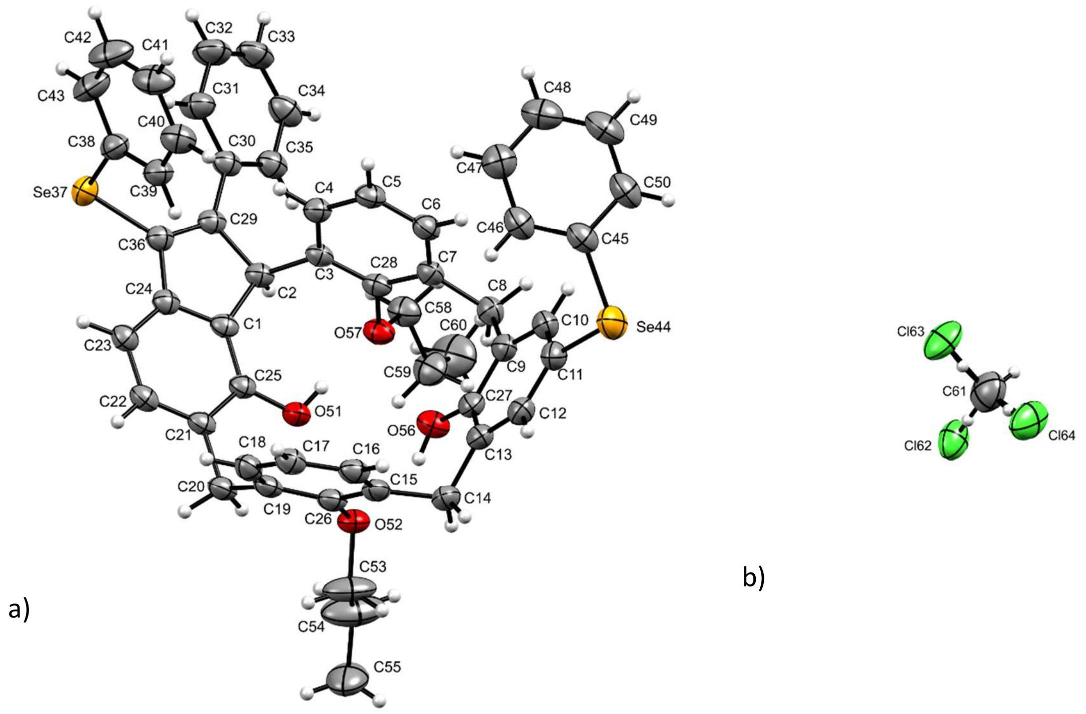

Figure S76: Numbering scheme of crystal structure of a) **8b** and b) solvate molecule, the ORTEP diagram with 50% probability.

3. Electrochemistry

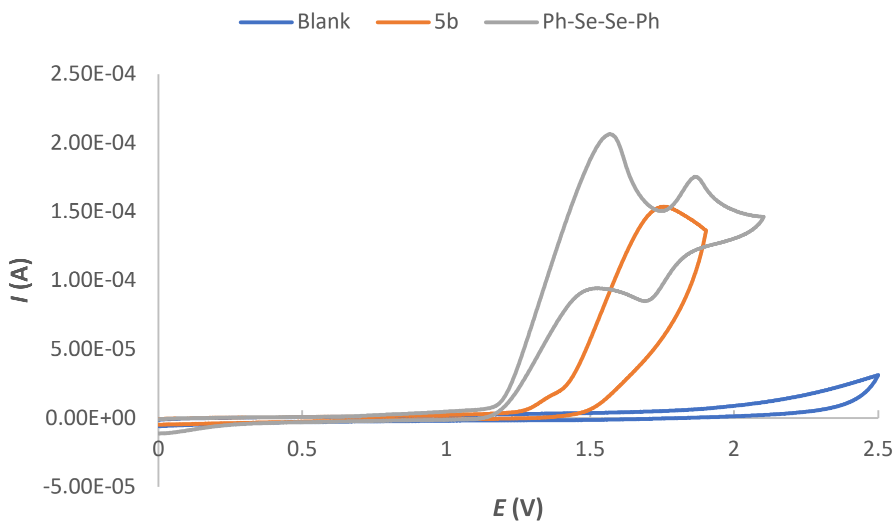

Figure S77: a) blue Cyclic voltammogram (W = GC, Ref = SCE, Aux = Pt) of blank solution (MeCN, 0.02 M $LiClO_4$) with scan rate 100 mV/s (IUPAC plotting convention). **b) orange** Cyclic voltammogram (W = GC, Ref = SCE, Aux = Pt) of compound **5b** (4.14 mM) in MeCN (0.02 M $LiClO_4$) with scan rate 100 mV/s (IUPAC plotting convention). **c) grey** Cyclic voltammogram (W = GC, Ref = SCE, Aux = Pt) of diphenyl diselenide (2.12 mM) in MeCN (0.02 M $LiClO_4$) with scan rate 100 mV/s (IUPAC plotting convention).

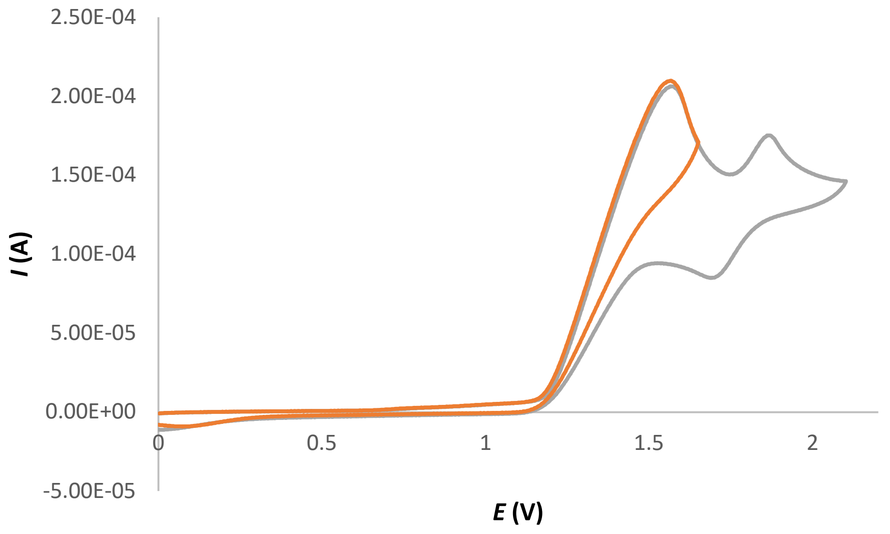

Fig. S78: Cyclic voltammograms (W = GC, Ref = SCE, Aux = Pt) of diphenyl diselenide (2.12 mM) in MeCN (0.02 M $LiClO_4$) with scan rate 100 mV/s (IUPAC plotting convention).

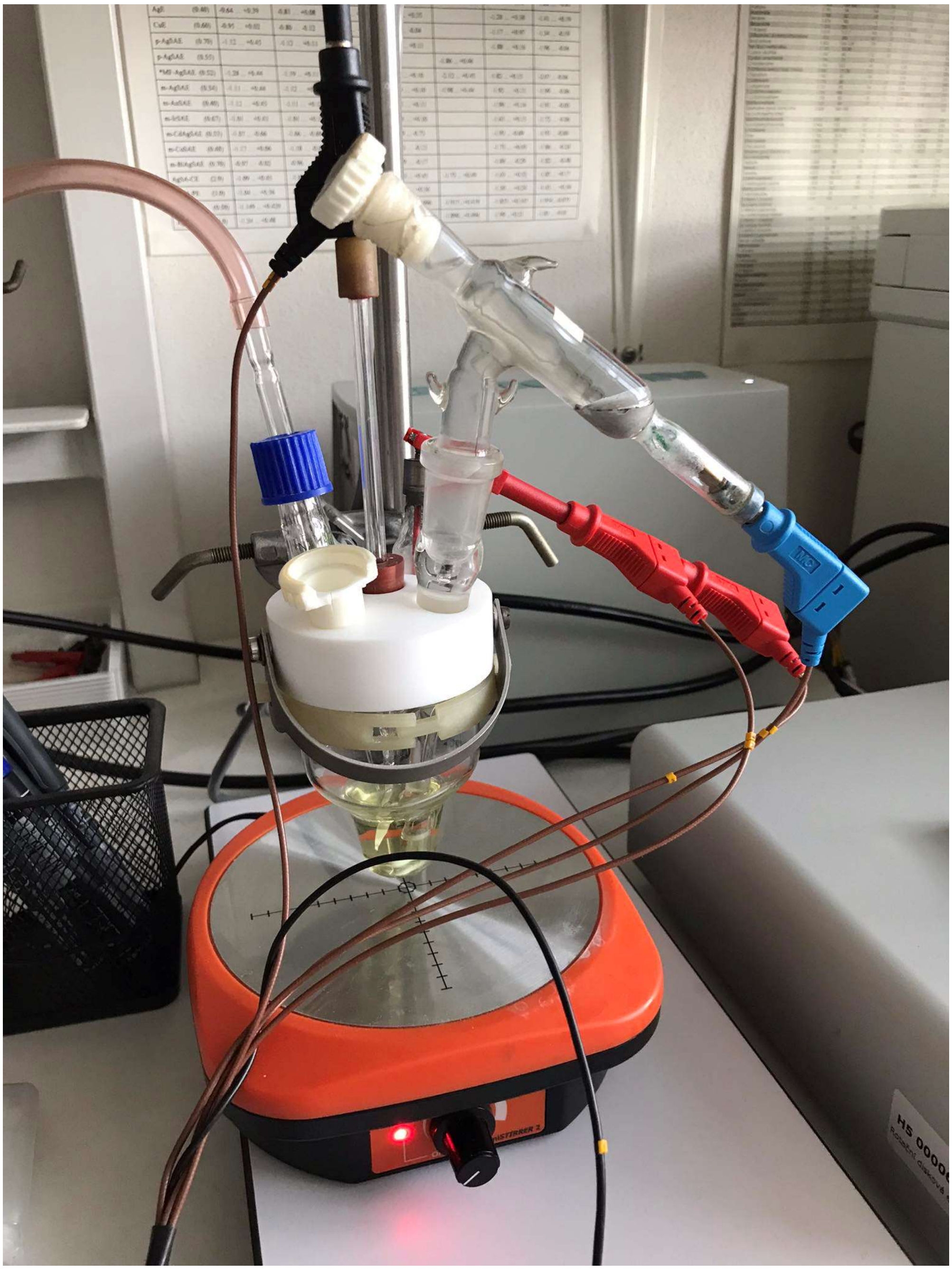

Figure S79: Illustrative picture for batch electrolysis of **5b** in undivided cell equipped with Pt plate used as a working electrode, and SCE utilized as reference electrode, and Pt plate counter electrode.
